# Macro and Trace Elements in Hemp (*Cannabis sativa* L.) Cultivated in Greece: Risk Assessment of Toxic Elements

**DOI:** 10.3389/fchem.2021.654308

**Published:** 2021-04-22

**Authors:** Effrosyni Zafeiraki, Konstantinos M. Kasiotis, Paul Nisianakis, Kyriaki Machera

**Affiliations:** ^1^ Laboratory of Pesticides’ Toxicology, Department of Pesticides Control and Phytopharmacy, Benaki Phytopathological Institute, Athens, Greece; ^2^ Chemical Laboratory, Athens Analysis Laboratories, Athens, Greece

**Keywords:** *Cannabis sativa* L., trace and macro elements, heavy metals, ICP-MS, THQ

## Abstract

The accumulation of hazardous contaminants in *Cannabis sativa* L. raises warning signs regarding possible adverse effects on human health due to the consumption of herbal medicines and/or other herbal edible products made from cannabis. Thus, there is an urge to investigate the levels of hazardous contaminants, such as heavy metals, in cannabis plant. In the present study, 29 macro and trace elements, including both beneficial and toxic elements (heavy metals and metalloids), were investigated in 90 samples of *Cannabis sativa* L*.* collected from Greece. According to the results, the detected concentrations of macro elements in the leaves/flowers of cannabis ranged between 28 and 138,378 ppm, and of trace elements between 0.002 and 1352.904 ppm. Although the concentrations of elements varied among the samples, their accumulation pattern was found to be similar, with the contribution of toxic elements to the total concentration of trace elements being below 1%. The detected levels of the most toxic elements were below the prescribed limits established by the WHO, while the calculated THQ and CR values showed no risk (non-carcinogenic and carcinogenic) for the population exposed to the current cannabis samples. Positive correlation between the concentration of elements and cannabis geographical origin and variety was observed. Cannabis leaves/flowers were more contaminated with trace and macro elements than seeds.

## Introduction

*Cannabis sativa* L*.* is one of the earliest and widely cultivated herbaceous plants. It contains more than 113 cannabinoids, among which cannabidiol (CBD) and tetrahydrocannabinol (Δ9-THC) are well known for their healing properties and medicinal use (Russo et al., 2007). Apart from its long-term use for the treatment of pain, spasms, asthma, insomnia, depression, and loss of appetite, nowadays, *Cannabis sativa* L. contributes also to the treatment of nausea and vomiting associated with cancer chemotherapy, spasticity in multiple sclerosis, and anorexia in HIV/AIDS (Colorado Department of Public Health and Environment (CDPHE), 2016). In addition, there is substantial evidence that cannabinoids are also effective in movement disorders and neuropathic pain (Grotenhermen and Muller-Vahl, 2012).

Besides its medicinal use, *Cannabis sativa* L. finds application in more than 25.000 products globally, including industrial cannabis (hemp) and numerous edible products (Salentijn et al., 2015). Cannabis seeds consist of 20–35% oil, which makes them appropriate for the production of cooking/seasonal oil, dietary supplements, plant-based superfoods, beverages, and also body care products, fuel, paint, *etc*., while the fiber of cannabis (stalk part) is useful for the production of textiles, insulators, ropes, paper, and biomaterials. Meanwhile, the increasing demand and use of its medicinal and food products has led to the harvest of mainly leaves and seeds in approximately 30 countries. Another viewpoint not to disregard is that the consumption of raw unprocessed cannabis is gaining ground as well, since it is believed that raw leaves and buds are also rich in nutrients (Żuk-Gołaszewska and Gołaszewski, 2018).

As cannabis can accumulate both natural and anthropogenic contaminants of high concern during its growth, it is considered a potential source of risk for human health (Fu et al., 2018; Craven et al., 2019; Atapattu and Johnson, 2020). In particular, in cannabis, trace and macro elements can build up, including also toxic ones, mainly *via* the soil and water in which it grows, or through the deposition of fertilizers, pesticides, and fungicides that are commonly applied to crops and contain such elements (Galic et al., 2019). The variety of the plant, harvesting time, geographical origin, topography, and duration of the exposure to the contaminants are factors playing an essential role in the accumulation of elements in the cannabis plant (Arpadjan et al., 2008; Nagajyoti et al., 2010).

The high applicability of *Cannabis sativa* L. in medicines and food products, combined with the ability of the plant to accumulate contaminants, has raised concerns regarding the safety of the product (Ware and Tawfik, 2005). According to the WHO, “This risk can be confined by ensuring that herbal medicines possessing harmful contaminants and residues do not reach the public, by evaluating the quality of the medicinal plants, herbal materials, and final herbal products before they reach the market” (World Health Organization, 2007).

To this end, the aim of the current study was to investigate the occurrence of trace and macro elements, including both beneficial and toxic elements (heavy metals and metalloids), in the leaves/flowers of 90 hemp samples collected from Greece (2018–2019), a representative Mediterranean country. Although the specific samples were intended for industrial purpose, their mineral composition can be used as a surrogate to potentially reflect the respective content of cannabis intended for human consumption. It is noteworthy that the main difference between hemp and other cannabis focuses on the differentiation of THC and CBD levels. In addition, the provisions for the production of medical cannabis in Greece are governed by the Greek Law 4523/2018 (OGG, 2018) that is in place from March 2018; hence, such samples were not relevant to this study. Consequently, the human health risk assessment of the most toxic elements is attempted and presented. Considering Greece’s geology and the diversity of the composition of the soil around the country, potential differentiations between elements’ accumulation and sampling location of cannabis are also investigated. In the same context, the variety of the plants is also investigated as another potential factor influencing the accumulation of elements in cannabis. In order to further examine the distribution of the elements among the different parts of cannabis, the leaves/flowers and the seeds of 21 samples are separated and further analyzed. For the analysis of all the cannabis samples and the detection of trace and macro elements in them, an inductively coupled plasma mass spectrometry (ICP-MS) is used.

Overall, the current study was conducted in an effort to fill knowledge and literature gaps of the particular scientific field being explored. It is important to stress that the novelty of the study lies both in terms of addressing the problem of metals’ occurrence in cannabis, which although of local character informs one of the very few relevant studies in the field, and in terms of establishing a multi-analyte method for the quantification of several minerals. What is more? To our knowledge, this is the first study presenting information on several trace and macro elements in such a large number of cannabis samples cultivated in Greece, by using an ICP-MS.

## Materials and Methods

### Sample Collection

**FIGURE 1:**
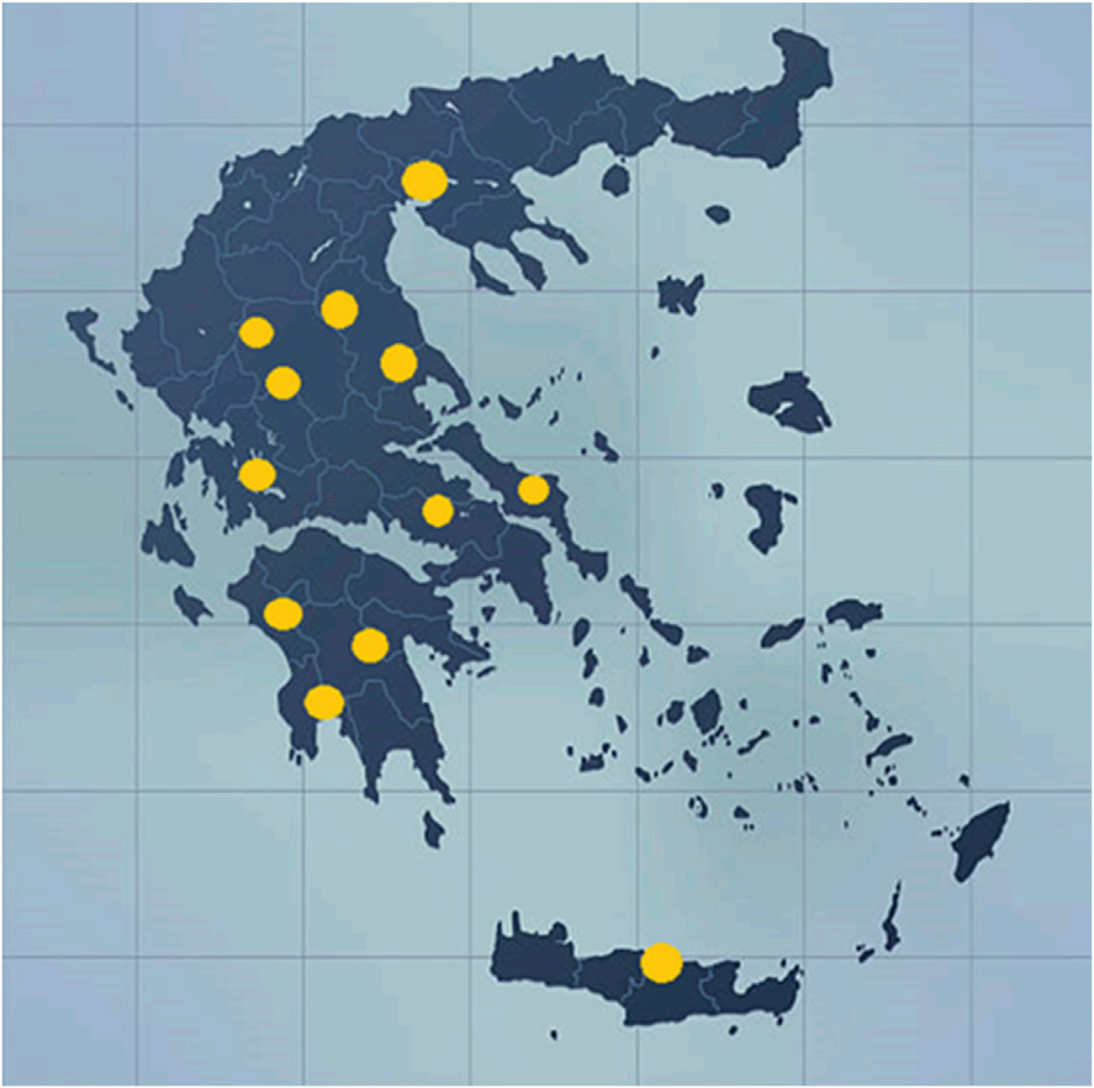
Map indicating the locations where the samples of *Cannabis sativa* L. were collected during 2018 and 2019.

Sampling was carried out during a 2-year period, that is, 2018–2019. Ninety samples of 9 different varieties of cannabis plants cultivated in 13 regions in Greece were collected by several producers and delivered to the Benaki Phytopathological Institute (Figure 1 and Supplementary Table S1). Upon arrival of the samples at the lab, 5 or more individual branches collected from each crop were pooled and further treated for their analysis. The varieties of cannabis collected were the following: Finola, Futura 75, Fedora 17, Gamagnola, Fellina 32, Dora, CS, Fibror 79, and Compolti.

### Chemicals

The analytical method applied in the current study is suitable for the quantification of 29 chemical elements: boron (B), sodium (Na), magnesium (Mg), aluminum (Al), phosphorus (P), potassium (K), calcium (Ca), titanium (Ti), vanadium (V), chromium (Cr), manganese (Mn), iron (Fe), cobalt (Co), nickel (Ni), copper (Cu), zinc (Zn), arsenic (As), selenium (Se), strontium (Sr), molybdenum (Mo), silver (Ag), cadmium (Cd), tin (Sn), antimony (Sb), barium (Ba), mercury (Hg), thallium (Tl), lead (Pb), and uranium (U). For the quantification of the elements, inductively coupled plasma mass spectrometry (ICP-MS) and internal standards (IS) were used. The standard solution/mixture of 25 components (Al, Ba, B, Cu, Fe, Sr, Zn, Be, Cr, Co, Li, Mn, Mo, Ni, Ti, V, Sb, As, Cd, Pb, Se, Ag, Tl, U, and Sn) and the standard solutions of individual elements (Hg, Na, Mg, Ca, P, and K) used for the preparation of two calibration curves, and the solution of elements (lithium (^6^Li), scandium (Sc), germanium (Ge), ytrium (Y), indium (In), terbium (Tb), and iridium (Ir)) used as internal standard, were all provided by CPAchem (Stara Zagora, Bulgaria). The standard solution/mixture of 25 components (Al, Be, Co, Li, SeL, Sn, Zn, Sb, B, Cu, Mn, Ag, Ti, As, Cd, Fe, Mo, Sr, U, Ba, Cr, Pb, Ni, Tl, and V) used for the preparation of quality assurance (QC) standard was also purchased from CPAchem (Stara Zagora, Bulgaria). The two CRMs, BCR-191 brown bread and BCR-679 white cabbage, were provided by the European Commission. Nitric acid (HNO_3_) 67–69%, hydrochloric acid 37% (HCl), and hydrogen peroxide (H_2_O_2_) for trace elements analysis/trace metal grade were all purchased from Seastar Chemicals Inc. (Sydney, Canada). Finally, ultrapure Milli-Q water was also used for the dilution of all the aforementioned solutions, when needed.

### Sample Preparation

All samples were dried overnight in the oven at 60°C, for the reduction of moisture content. To separate the parts of the plant, the dried cannabis samples were sieved, and the leaves/flowers were further selected. The seeds of 21 samples were further separated and subjected to the same treatment as leaves. The selected part of cannabis samples was then grounded by an electronic blender, and the final powder was kept into polyethylene plastic bags for the avoidance of contamination/adsorption of elements until their digestion.

The digestion of all the samples was performed in a microwave oven (MARS 5, CEM). In particular, 0.25 g of each sample were weighted and placed into a vessel. 5 ml HNO_3_, 3 ml ultrapure water, 2 ml HCl, and 2 ml H_2_O_2_ were then added in each vessel, and the samples were further digested into the microwave digestion system. The duration of the digestion process for each sample was 40 min, with a ramp time of 25 min. The maximum microwave power and temperature applied were 800 W and 180°C, respectively. After the end of the digestion process, the vessels were cooled to less than 40°C into a clean hood, while excess pressure was vented slowly. The digestion solution was quantitatively transferred to a clean container, and ultrapure water was added until a final volume of 100 ml was reached.

### Instrumental Analysis

All the cannabis samples were analyzed by using inductively coupled plasma mass spectrometry (ICP-MS)-Thermo iCAP-RQ, equipped with an ASX-280 autosampler. Analysis was performed by applying collision cell mode (kinetic energy discrimination (KED)), using He (collision gas flow: approximately 4.78 ml/min (autotune dependent)), to selectively attenuate all polyatomic interferences based on their size. The instrument used Ni sample and skimmer cones, MicroMist U-Series Nebulizer (0.4 ml/min with PEEK connector), and a Quartz cyclonic spray chamber. Plasma power was equal to 1550 W, while the flow of the nebulizer gas and the cool gas was approximately 1 L/min (autotune dependent) and 14 L/min, respectively. The preferred isotopes and the corresponding internal standard for each isotope used are presented in Supplementary Table S2.

Prior to the analysis of the samples, the ICP-MS system was allowed to equilibrate for 30 min, and then, the sensitivity and the stability of the instrument were checked in KED mode by using tune solution containing 1 μg/L (each) of Ba, Bi, Ce, Co, In, Li, and U in 2% HNO_3_ and 0.5% HCl. Then, a performance test in KED mode was performed using the same tune solution. When it was necessary, autotune and calibration mass tests were also performed, in order for the equipment to be optimized. To this end, the analysis of the samples was further conducted with high sensitivity, stability of signal, and low levels of doubly charged ions and cluster ions.

### Quantification and Quality Assurance

Calibration curves covering concentrations from 0.1 to 1,000 μg/kg and from 0.1 to 200 mg/kg were prepared to be matched with the expected concentration ranges of trace elements (B, Al, Ti, V, Cr, Mn, Fe, Co, Ni, Cu, Zn, As, Se, Sr, Mo, Ag, Cd, Sn, Sb, Ba, Hg, Tl, Pb, and U) and macro elements (Na, Mg, P, K, and Ca) in the samples, respectively. The coefficient of determination (r^2^) was greater than 0.999 for all the calibration curves. Gold (Au) was added in all the calibration standards for the stabilization of mercury, while internal standards (^6^Li, Sc, Ge, Y, In, Tb, and Ir) were added at a constant rate and concentration to all unknown samples and calibration standards.

The limit of detection (LOD) was the concentration value corresponding to three times the standard deviation obtained from the consecutive measurements of 10 reagent blanks, while the limit of quantification (LOQ) was equal to ten times the standard deviation of the latter. Verification of LOQ values was also made by the analysis of 12 replicates of spiked aqueous solutions. The final LOQ values and the estimated uncertainty (u’) for each element are presented in Supplementary Table S2.

BCR-CRM 191 brown bread and BCR-CRM 679 white cabbage were measured in the same batch with the unknown samples in order to monitor and assure the accuracy of the measurement. Bread and cabbage were chosen as reference materials due to their similarity to the matrix of cannabis plant. For further verification of the accuracy of the method, the certified reference materials and two samples were analyzed by two different laboratories, both applying microwave digestion and ICP-MS for the analysis of the samples. No significant differences were observed between the obtained results of the two labs. In addition, two QC standard solutions of macro and trace elements, respectively, were measured in every sequence. The recovery of the two CRMSs, the two QCs, and the internal standards ranged between 80 and 120% in all the samples, verifying the sufficient ionization of the elements and the absence of matrix effect. For the avoidance of spectral interferences, one additional isotope was measured when possible, while reagent blanks were prepared under the same conditions as the samples and measured in every batch.

### Statistical Analysis

Statistical analysis was performed by using the SPSS software (IBM SPSS statistics for Windows). The Shapiro–Wilk test was performed in order to investigate the normality of variance between the element concentration and the sampling location, and between the former and the variety of cannabis samples. The nonparametric Kruskal–Wallis test was also applied in order to examine statistically significant differences between the concentration of each individual element and the sampling locations or the variety of the samples. The statistical significance level was acceptable at *p* < 0.05. Possible correlation of each element concentration between the leaves and the seeds of the same cannabis plants was also investigated by applying the Pearson correlation coefficient test.

### Human Health Risk Assessment

Although there are several pathways through which humans can be exposed to heavy metals and trace elements, the main route is the dietary consumption (ingestion), representing a 90% of the overall health risk (Doabi et al., 2018; Guo et al., 2020). Hence, in the presented work, calculations for risk assessment were regarded only by this pathway.

In the current study, the estimated daily intake (EDI) of the most toxic elements was calculated for both non-carcinogenic and carcinogenic risks, based on the following equation (Eq. 1):

$$EDI=\frac{FIR\times C\times EF\times ED\times CF}{BW\times AT},$$ (1)

where C is the concentration of the element (mg/kg), FIR (food intake rate): 3 g per person per day, EF (exposure frequency): 365 days/year, ED (exposure duration): 27 and 70 years for non-carcinogenic and carcinogenic risk, respectively, AT (average exposure time (crop)) for noncarcinogens: 365 days*ED, BW (average body weight): 70 kg for adults, and CF: unit conversion factor.

#### Non-carcinogenic Risk

The target hazard quotient (THQ) was further estimated, for the investigation of the potential non-carcinogenic risk of cannabis samples. The calculation of THQ was based on the following equation (Eq. 2):

$$THQ=\frac{EDI}{RfD},$$ (2)

where RfD is the oral reference dose (mg/kg/day).

Cumulative health risk was assessed by calculating the hazard index (HI). The latter was derived from the summation of all separate THQs (k = number of heavy metals regarded), considering the ingestion pathway using the equation below (Eq. 3):

$$HI=\sum_{k=1}^{n}THQ_{k}.$$ (3)

#### Carcinogenic Risk

The carcinogenic risk accounts for the cumulative probability of developing cancer in an individual’s lifetime as a result of the exposure to a potential carcinogen. Consequently, two endpoints were considered: the EDI of each toxic element and the oral cancer potency (or slope) factor (CPF_o_). To determine the EDI for carcinogenic risk, the same equation was used (Eq. 1), projecting AT to the average lifetime (365 days * 70 years = 25,550 days). CPF_o_ represents a metric of cancer risk, defined as the justifiable upper-bound estimate of the probability that an individual will develop cancer if exposed to a chemical for a lifetime of 70 years (Farris and Ray, 2014). Values were retrieved mainly from the US Environmental Protection Agency (US EPA, 2020), the Office of Environmental Health Hazards Assessment (OEHHA, 2020), and the Risk Assessment Information System (RAIS, 2020). In this regard, the cancer risk (CR) was assessed using the following equations (Eqs. 4, 5)

$$CR_{k}=EDI\times CPF_{o},$$ (4)

$$CR=\sum_{k=1}^{n}CR_{k}.$$ (5)

CR was considered negligible when it was below 1 × 10^–6^, and likely harmful when above 1 × 10^–4^. Values within the range 1 × 10^–6^ to 1 × 10^–4^ signify an acceptable or tolerable risk.

## Results and Discussion

### Levels of Trace and Macro elements in the Leaves/Flowers of *Cannabis Sativa* L

**FIGURE 2:**
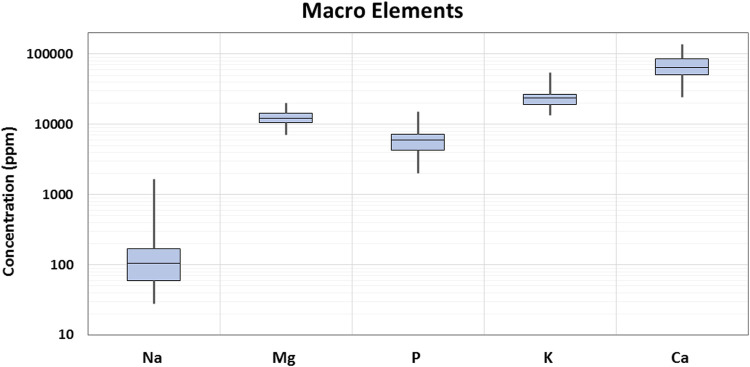
Box plot of macro elements’ concentration in cannabis samples.

**FIGURE 3:**
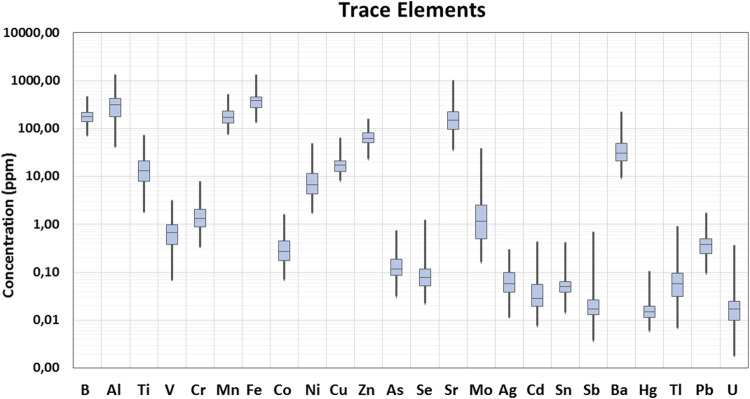
Box plot of trace elements’ concentration in cannabis samples.

In the current study, 29 elements were quantified in 90 samples of *Cannabis sativa* L. leaves/flowers. The distribution of the detected concentrations of the analyzed elements and the skewness are shown through displaying the data quartile and averages (Figures 2, 3), while the concentrations of individual elements for each of the cannabis sample are illustrated in Supplementary Table S3.

The elements quantified can be divided into two main categories based on their toxicity: macro elements (Mg, Na, K, P, and Ca) and trace elements (B, Al, Ti, V, Cr, Mn, Fe, Co, Ni, Cu, Zn, As, Se, Sr, Mo, Ag, Sn, Sb, Ba, Tl, and U), which are mainly essential for the body and thus for human health, and toxic heavy metals and metalloids (Pb, Cd, Hg, and As) that are trace elements characterized by high toxicity and also accused for the cause of severe health effects.

More specifically, macro elements are nutritionally important minerals and contribute to the normal growth and function of the human body. In the current study, Ca was the most predominant element in terms of detected concentration. The highest Ca concentration was found in a Fedora 17 sample from Hrakleio, and it was equal to 138,378 mg/kg. The next element with the highest concentrations observed was K. According to the results, K concentration ranged between 13,459 and 54,393 mg/kg. Mg minimum and highest concentrations detected in the current cannabis samples were 7,140 and 20,202 mg/kg, respectively. In the same context, the concentration of two other essential elements for the body, P and Na, ranged from 2,018 to 15,088 mg/kg, and between 28 and 1,672 mg/kg, respectively.

Apart from macro elements, there are also essential trace elements, such as Fe, Zn, Cr, Mn, Co, Cu, Mb, Ni, and Se (Prashanth et al., 2015). According to the current findings, Fe, Mn, and Zn had the highest concentrations among the trace elements quantified. Fe maximum concentration was found in a Fibror 79 sample from Trikala and was equal to 1,338 mg/kg, while its average concentration was 401 mg/kg. In cannabis samples, Zn concentration ranged between 23.1 and 158 mg/kg, with the latter being detected in a sample of CS variety collected from Messinia. The detected average concentration of Mn in the leaves of cannabis analyzed was equal to 195 mg/kg.

Ni is recognized as an essential nutrient for the proper growth of plants. However, depending on the way of its assumption into the organism of humans, and to the amount, duration of contact, and route of exposure, Ni can also be responsible for several adverse effects, such as asthma, dermatitis, gastrointestinal manifestations, cardiovascular diseases, lung fibrosis, respiratory track cancer, and nasal cancer (Genchi et al., 2020). The International Agency for Research on Cancer (IARC) has already classified soluble and insoluble Ni compounds as Group 1 (carcinogenic to humans) (IARC, 2012).

In the same context, although Cu is an essential trace element for the body and also a nutrient for plants, human exposure *via* digestion of high levels of copper, around 70 mg/day, can lead to serious adverse effects on health, including liver damage and gastrointestinal symptoms (National Research Council Committee on Copper in Drinking Water, 2000; World Health Organization, 2007). Considering that Cu is strongly bioaccumulated in nature, the likelihood of exposure to copper is heightened. Thus, the WHO recommends the control of copper levels in plants, like herbal ones, that are likely to persist Cu (World Health Organization, 2007).

Similar to Cu and Ni, Cr can be both essential and toxic. The toxicity of Cr depends on the oxidation state of the metal. In particular, Cr (VI) has been associated with increased incidents of lung cancer, DNA damage, chromosomal aberrations, and alterations in the epigenomic instability (Zhitkovich, 2005), while Cr (III) is an essential nutrient, playing an important role in glucose and lipid metabolism. Even though the use of large doses of Cr (III) supplements contributes to the improvement of glucose metabolism, there is a growing concern over the possible genotoxicity of these compounds (Stearns, 2000).

Cetain heavy metals (Hg, Pb, and Cd) and metalloids (As) are also bioaccumulated in nature and food commodities, provoking toxic effects even at low concentrations and regardless of their oxidation state. Indicatively, mean concentrations of Hg in several fish species (from United States, Canada) varied from 0.095 to 0.976 mg/kg; hence, in some cases, the maximum permissible concentration (or action level) of 1 mg/kg (or ppm) ascribed by the US Food and Drug Administration was approximated (Rice et al., 2014, and references therein). Biomonitoring studies showed for Hg a cord blood concentration of 0.085 mg/L to be linked to early neurodevelopmental effects (Centers for Disease Control and Prevention (CDC), 2019). Nevertheless, the reported mean levels of Hg in blood in the US population from 2003 to 2016 (National Biomonitoring Program) were lower than the aforementioned concentration (Centers for Disease Control and Prevention (CDC), 2019). In the same context, for Cd, the highest measured urine concentrations (mean levels did not surpass 0.06 μg/L) were in proximity, but did not supersede the levels associated with indications of kidney alterations (Centers for Disease Control and Prevention (CDC), 2019). Human exposure to Cd mainly occurs due to the consumption of contaminated food, inhalation of tobacco smoke, and inhalation by workers in a range of industries (Rahimzadeh et al., 2017). In particular, Cd predominantly accumulates in the kidney and liver, exerting toxic effects on these organs. Cd can cause oxidative stress (Cuypers et al., 2010; Patra et al., 2011), epigenetic changes in DNA expression (Wang et al., 2012), renal dysfunction, diabetes (Edwards and Prozialeck, 2009), hypertension (Gallagher and Meiker, 2010), and impair vitamin D metabolism (Joint FAO/WHO Food Standards Program, 2011). Regarding Pb, it has been blamed for a wide range of biological effects, including hematological, neurological, behavioral, renal, cardiovascular, and reproductive system effects (Flora et al., 2012). Depending on the level and duration of exposure, symptoms can vary, while children are more vulnerable to the effects of Pb than adults. According to the WHO, “lead exposure can have serious consequences for the health of children. At high levels of exposure, Pb attacks the brain and the central nervous system to cause coma, convulsions, and even death” (World Health Organization, 2019; Joint FAO/WHO Food Standards Program, 2011). Hg or methylmercury [MeHg]^+^ is toxic for the central and peripheral nervous system. The inhalation of Hg vapor can cause harmful effects in the nervous system, lungs, kidneys, and digestive and immune system, or even ends up to become fatal. Neurological and behavioral disorders, motor dysfunction, memory loss, and headaches have been already observed after the inhalation, ingestion, or dermal exposure to Hg (World Health Organization, 2017). Moreover, long-term exposure, of minimum five years, to As usually leads to skin lesions and cancer, while cancer in the bladder and lungs is also possible. Other adverse effects that maybe related to the long-term ingestion of As include diabetes, cardiovascular disease, developmental effects, adverse pregnancy outcomes, and infant mortality. On the other hand, the immediate symptoms of acute As poisoning, that is, the exposure to As occurring over a short period of time (often less than a day), include vomiting, abdominal pain, and diarrhea, followed by numbness of the extremities and muscle cramping, and maybe death (World Health Organization, 2018).

Considering that toxic metals are abundant on nature, they are likely to be present in many foods, and thus the ensurance of the safety of herbal products is of major importance. To this end, the WHO has already established guidelines in order to assess the quality of herbal medicines and products and prescribed maximum concentration limits for the toxic elements (As, Pb, Cd, Cr, and Hg) (World Health Organization, 2007). Recommendation levels for Cu and Ni in raw herbal materials are not established yet, and thus these elements are not included in Table 1.

**TABLE 1:** Concentration ranges of the most toxic elements compared with the maximum limits set by the WHO.

Element	Minimum concentration (ppm)	Maximum concentration (ppm)	Average of C (ppm)	WHO 2007 recommendation levels (ppm)^a^
Hg	0.006	0.107	0.020	0.2
Cd	0.007	0.431	0.049	0.3
Pb	0.095	1.752	0.433	10
As	0.031	0.742	0.159	5
Cr	0.337	7.886	1.686	2

^a^ Canadian values

By comparing the obtained levels of key toxic elements with the limits prescribed for raw herbal materials (intended for herbal medicines use) by the WHO, it was found that none of them exceeded the prescribed limits (Table 1). In particular, the concentrations of Hg, Pb, Cd, and As were found to be lower than the standards in all the samples, except for one sample where Cd concentration was 1.4 times higher than the recommended level. The obtained levels of Hg, Pb, Cd, and As are not believed to comprise a risk for human health, based on the mentioned established limits.

Apart from the elements above, Cr concentrations were also compared to the limits prescribed by the WHO, and it was observed that the levels of Cr exceeded the limit in approximately 25% of the analyzed samples. To this end, further investigation on the quality assurance of cannabis plants before their use in medicinal and edible products is strongly recommended, in order for humans to avoid chronic exposure to toxic elements, such as Cr.

The current results are in agreement with a previous study investigating heavy metals in medicinal plants, including *Cannabis sativa* L., in which Pb and Cr concentrations exceeded the limits set by the WHO, raising warning signs regarding the ingestion of herbal medicines (Kumar et al., 2018).

However, both in the present study and in Kumar’s study, Cr levels were higher than the standards of the WHO, and Cr current concentrations (range: 0.337–7.89 mg/kg) were lower than the ones detected in the Kumar’s study (range: 17.6–58.6 mg/kg). In the same context, other studies focusing on metals in *Cannabis sativa* L. reported higher levels of heavy metals than the ones of the present study (Zerihum et al., 2015). On the other hand, in another study, quantifying toxic elements in medicinal food homologous plants, the detected levels of As, Cd, Hg, and Pb in the fruit of *Cannabis sativa* L. were at the same range with the current ones (Fu et al., 2018). Similarly, in a previous study examining the levels of macro and microelements in various herbs, Cd detected concentration was close to the current findings (Moghaddam et al., 2020). In contrast, the mean Pb concentration in Moghaddam’s study was equal to 6.32 mg/kg, while in the present study, it was 0.430 mg/kg. Regarding the rest of the common elements analyzed in the two aforementioned studies, their concentration was in most of the cases lower than that in the present study.

Finally, it is worth mentioning that a more extended comparison of cannabis plants’ contamination with elements is hampered due to the insufficient available data. Thus, the collection and analysis of more cannabis samples would be necessary in order to reach a more solid conclusion.

### Element Pattern

**FIGURE 4:**
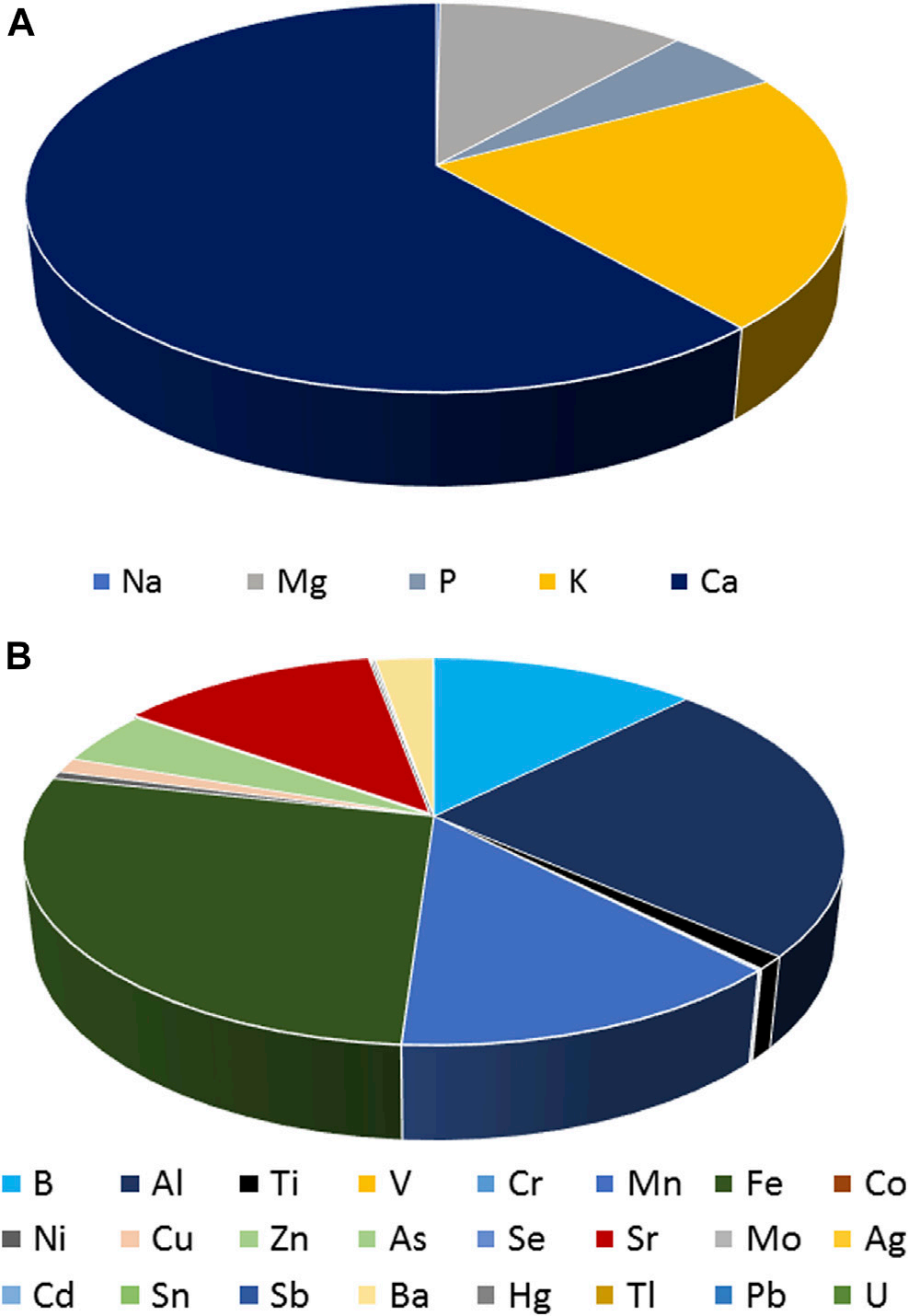
Average contribution (%) of macro and trace elements among all the cannabis samples.

Despite the difference in levels, the pattern of the elements was found to be similar among all the cannabis samples, with Ca and Fe being the most predominant elements in macro and trace elements, respectively. The contribution of each macro and trace element to the total concentration of elements was calculated based on the average concentration of each element and is presented in the following figures (Figure 4).

More specifically, Ca and K were found to contribute more than the other macro elements to the total concentration of the latter in all the cannabis samples. In particular, Ca contributed 61%, followed by K (21%) and Mg (11%). As far as trace elements are concerned, Fe and Al were detected in the highest concentrations, covering 27 and 24% of the total detected concentrations in all the cannabis samples.

At this point, it is worth mentioning that the content in toxic heavy metals and metalloids was very low in all the cannabis samples (below 1%).

### Geographical Origin and Variety of Cannabis Plants

The concentration of macro and trace elements accumulated by cannabis varies, depending on several factors, such as the type and the variety of the plant, the geographical origin (soil) where the plant grew, the application of pesticides and fertilizers, the drying methods of the hemp, and the storage conditions (Galic et al., 2019).

In the current study, the samples were collected from 13 different locations around Greece (Aitoloakarnania, Arkadia, Messinia, Magnisia, Evoia, Voiwtia, Thessaloniki, Hrakleio, Hleia, Trikala, Karditsa, Larisa, and Korinthia) and consisted of 9 different varieties of cannabis (Finola, Futura 75, Fedora 17, CS, Dora, Carmagnola, Compolti, Fibror 79, and Fellina 32) (Supplementary Table S1).

In order to investigate a possible correlation between the detected concentration of elements in each sample and the geographical origin of the samples, cannabis plants were grouped based on the location in which they grew, regardless of their variety. A Shapiro–Wilk test (*p* > 0.05) and a visual inspection of their normal Q–Q plots showed that element concentrations were not normally distributed among the sampling locations. To this end, the nonparametric Kruskal–Wallis test was further applied. According to the result, a statistically significant difference was observed between the concentration of Na, K, Ca, B, Mn, Ni, Cu, Zn, As, Sr, Mo, Cd, Ba, Tl, and U, and the geographical origin of the samples. For the rest of the elements, the *p* value was higher than 0.05, revealing that the difference between their detected levels and sampling points was insignificant. More specifically, the locations among which a statistically significant difference for the aforementioned elements was observed are illustrated in the Supplementary data (Supplementary Figure S1).

The current findings were in agreement with a previous study, showing that the sampling site influences the accumulation of most of the analyzed metals in *Cannabis sativa* L. (Zerihum et al., 2015). This could be attributed to the different soil composition of each area, and also to the pesticides or fertilizers used for the cultivation of *Cannabis sativa* L. in each field. However, since soil was not investigated in the current study, and the lists of pesticides/fertilizers applied in the fields were not available, further research on this topic is recommended.

Similar to geographical origin, the variety of cannabis was found to influence the accumulation of certain elements in the plant. Normal Q–Q plots and Shapiro–Wilk test were applied for the evaluation of the normality among the different cannabis varieties. According to the results, the samples were not normally distributed, and thus, a nonparametric Kruskal–Wallis test was further applied, showing statistically significant difference (*p* < 0.05) between the concentrations of Na, K, Ca, Ni, Cu, As, Sr, Mo, Cd, Ba, and U, and the varieties of the samples. More specifically, the varieties among which there was a statistically significant difference are presented in Supplementary Figure S2, for each individual element.

The current variation in metals’ accumulation among cannabis varieties could be attributed to different anatomical and chemical characteristics of the plant, such as soil type, stage of growth, and metals absorbed (Verma et al., 2007; Olowoyo et al., 2012). The current findings are in accordance with a previous study, presenting differentiation of heavy metals concentration among different medicinal plants, including *Cannabis sativa* L. (Kumar et al., 2018).

### Distribution of Elements Between Leaves/Flowers and Seeds of Cannabis Plant

In 21 out of the 90 samples, both leaves/flowers and seeds were separated and further analyzed for the investigation of elements distribution between the two parts of the plant. Leaves/flowers were found to accumulate more trace and macro elements than the seeds of the same sample, regardless of the cannabis variety and the location where the plant grew (Supplementary Table S4). The current results were in consistency with a previous study reporting that the levels of As, Cd, Cr, Fe, Ni, and Hg in leaves exceeded those of cannabis seeds (Eboh and Thomas, 2005). For the investigation of a possible correlation of the concentration of each element between leaves and seeds, Pearson correlation tests were applied. According to the results, the correlation between the two groups was found to be significant (*p* < 0.05) for the following elements: Na, Al, Mn, Co, Ni, Cu, Zn, As, Se, Sr, Mo, Cd, Ba, Tl, and U (Supplementary Figure S1). At this point, it is worth mentioning that among the most toxic elements, As and Cd were the only ones presenting significant correlation between leaves and seeds. In addition, further investigation on elements’ accumulation in the different parts of *Cannabis sativa* L. is strongly recommended since the obtained information could contribute to the assurance of the safety of edible cannabis products made of these parts of the plant.

### Human Health Risk Assessment

#### Estimated Daily Intake (EDI)

To our knowledge, there is limited information available on the consumption of edible cannabis products. Most of the studies reporting an intake rate of cannabis are mainly focused on the dosage of medical cannabis. Based on these studies, the daily food consumption rate of smoked or orally ingested cannabis for medical purposes was approximately 0.65–3 g of dried cannabis per person per day (Government of Canada, 2016).

To investigate the estimated daily intake (EDI) at the worst-case scenario, an assumption was made, and the maximum reported amount of cannabis (3 g) was used. Based on Eq. 1, the EDIs of As, Cd, Hg, Pb, Cr, and Ni were calculated based on the concentration of each element in each cannabis sample. Children and adolescents were not regarded in the health risk assessment since they are not considered major “consumers” of cannabis-containing products (particularly of raw cannabis). The calculated EDIs of each element are presented in Supplementary Table S5.

#### Non-carcinogenic Risk

Based on the previous calculations, the target hazard quotient (THQ) was next estimated (Eq. 2). The oral RfD values for each toxic element (As, Cd, Hg, Pb, Cr, and Ni) were retrieved from the European Food Safety Authority (European Food Safety Authority (EFSA), 2009; European Food Safety Authority (EFSA), 2012a; European Food Safety Authority (EFSA), 2012b) and US EPA and were equal to 0.3, 1, 0.1, 2, 3, and 20 μg/kg b.w. per day for As, Cd, Hg, Pb, Cr, and Ni, respectively.

According to the results, all the THQ values were far below 1. Consequently, the HI values, as a metric of the quantified risk, were also below 1, indicating that there is no significant risk of non-carcinogenic effects for the population exposed to the current cannabis samples, and thus to their products (Supplementary Table S5). Similar to this outcome, a previous study displayed that the intake of various plants and spices could not cause significant health hazard to adults (Moghaddam et al., 2020).

#### Carcinogenic Risk

Pb, Cd, Cr, Hg, Ni, and As are classified as carcinogenic, while based on the International Agency for Research on Cancer (IARC, 2012), Cd, Cr, Ni, and As belong to category 1 of heavy metals inducing cancer. For Cd and Hg, CPF_o_ is not yet assigned. Consequently, the carcinogenic risk was calculated only for Pb, Cr, Ni, and As, using the CPF_o_ values depicted in Supplementary Table S7. The results of CR values (Supplementary Table S6) showed a negligible risk, since these values were far lower than the threshold value of 1 × 10^–6^ which US EPA (US EPA, 2020) suggests. Similar conclusion was derived after summing all individual CR values.

## Conclusion

To our knowledge, the current study is the first study presenting insights to the occurrence of macro and trace elements in a substantial number of *Cannabis sativa* L. (hemp) samples collected from different locations around Greece. The analysis of 90 samples of 9 varieties of cannabis showed that all cannabis samples analyzed contained both macro and trace elements in their leaves/flowers. Even though the detected concentrations of elements varied among the samples, the levels of the most toxic heavy metals and metalloids were below the maximum limits established by the WHO in all of the analyzed samples, indicating that no human health risk can be provoked at the first tier due to the consumption of medicines and edible products based on the current cannabis samples. Since cannabis is consumed raw and its products appear on the market for human consumption either as medicine or as food products, it is important to understand what is the resulting exposure to elements due to the consumption of these products, and the subsequent human health risk. In this context, the EDI, THQ, HI, and CR were further calculated addressing the most toxic elements. According to the results, there is no risk (non-carcinogenic and carcinogenic) for the population exposed to the current cannabis samples, and consequently to their products. Furthermore, an investigation of elements’ accumulation among the leaves/flowers and seeds of the same samples was made in order to provide information regarding the contamination (and its distribution) of the raw material and further contribute to the safety assurance of the edible cannabis products made from cannabis leaves and seeds. In addition, the present study showed positive correlation between elements detected concentration and cannabis geographical origin and variety. However, more research is needed to further deepen the knowledge on elements accumulation in cannabis plants, and as a consequence to their entrance into the food chain and then to the human organism.

## Data Availability

The original contributions presented in the study are included in the article/Supplementary Material; further inquiries can be directed to the corresponding authors.
