# Supplementary material for: Macro and Trace Elements in Hemp (Cannabis sativa L.) Cultivated in Greece: Risk Assessment of Toxic Elements
Source: Front Chem. 2021 Apr 22;9:654308. doi: 10.3389/fchem.2021.654308 (PMC8100522; doi:10.3389/fchem.2021.654308)
Supplement: Supplementary file 1 [file DataSheet1.docx]

**SUPPLEMENTARY DATA to the paper entitled:**

**Macro and trace elements in hemp *(Cannabis sativa L.)* cultivated in Greece – Risk assessment of toxic elements**

**Effrosyni Zafeiraki^1,*^, Konstantinos M. Kasiotis^1^, Paul Nisianakis^2^, and Kyriaki Machera^1,*^**

^1^ Laboratory of Pesticides’ Toxicology, Department of Pesticides Control and Phytopharmacy, Benaki Phytopathological Institute, Athens, Greece

^2^ Chemical Laboratory, Athens Analysis Laboratories, Athens, Greece

To whom correspondence should be addressed. E-mail: [e.zafeiraki@bpi.gr](mailto:e.zafeiraki@bpi.gr), k.machera@bpi.gr

This document provides more detailed information to the main paper mentioned above. The following information includes:

| **Content** |  | **Page** |
| --- | --- | --- |
| Table S-1 | Cannabis sativa L. varieties and geographical origin. | S2 |
| Table S-2 | Quantification parameters of trace and macro elements. | S3 |
| Table S-3 | Elements detected concentrations in the leaves/flowers of all the cannabis samples. | S5 |
| Figure S-1 | Box plots of each element concentration among the sampling areas. | S10 |
| Figure S-2 | Box plots of each element concentration among the different varieties of cannabis. | S13 |
| Table S-4 | Concentrations of elements in leaves/flowers and seeds of cannabis samples. | S15 |
| Figure S-3 | Accumulation of elements in leaves and seeds. <LOQ values are set at 0 ppm. | S17 |
| Table S-5 | Calculated EDI and HQ for the toxic elements. | S22 |
| Table S-6 | Calculated EDI and CR for the toxic elements. | S25 |
| Table S-7 | Oral Cancer Potency Factors (CPFo) | S27 |

**Table S1:** Cannabis sativa L. varieties and geographical origin.

| **2018** |  |  | **2019** |  |  |  |  |  |
| --- | --- | --- | --- | --- | --- | --- | --- | --- |
| **No of sample** | **Geographical origin** | **Variety** | **No of sample** | **Geographical origin** | **Variety** | **No of sample** | **Geographical origin** | **Variety** |
| **1** | Voiwtia | Finola | **29** | Larisa | Futura 75 | **60** | Korinthia | Finola |
| **2** | Thessaloniki | Finola | **30** | Trikala | Fellina 32 | **61** | Karditsa | CS |
| **3** | Voiwtia | Finola | **31** | Trikala | Fellina 32 | **62** | Korinthia | Finola |
| **4** | Messinia | Finola | **32** | Trikala | Futura 75 | **63** | Evoia | Carmagnola |
| **5** | Voiwtia | Finola | **33** | Trikala | Fibror 79 | **64** | Evoia | CS |
| **6** | Messinia | Futura 75 | **34** | Larisa | Futura 75 | **65** | Magnisia | Carmagnola |
| **7** | Magnisia | Fedora 17 | **35** | Larisa | Futura 75 | **66** | Magnisia | Compolti |
| **8** | Aitoloakarnania | Finola | **36** | Larisa | Fututra 75 | **67** | Magnisia | CS |
| **9** | Aitoloakarnania | Finola | **37** | Larisa | Fedora 17 | **68** | Trikala | Futura 75 |
| **10** | Aitoloakarnania | Finola | **38** | Messinia | Futura 75 | **69** | Trikala | Fututra 75 |
| **11** | Aitoloakarnania | Finola | **39** | Karditsa | Fututra 75 | **70** | Trikala | Fututra 75 |
| **12** | Aitoloakarnania | Finola | **40** | Karditsa | Fututra 75 | **71** | Trikala | Fibror 79 |
| **13** | Aitoloakarnania | Finola | **41** | Karditsa | Dora | **72** | Trikala | Fibror 79 |
| **14** | Thessaloniki | Finola | **42** | Larisa | Futura 75 | **73** | Trikala | Fibror 79 |
| **15** | Hleia | CS | **43** | Larisa | Futura 75 | **74** | Larisa | Futura 75 |
| **16** | Hleia | Futura 75 | **44** | Trikala | Futura 75 | **75** | Larisa | Dora |
| **17** | Korinthia | Carmagnola | **45** | Trikala | Futura 75 | **76** | Αrkadia | Compolti |
| **18** | Hleia | Futura 75 | **46** | Larisa | Futura 75 | **77** | Αrkadia | Carmagnola |
| **19** | Evoia | Carmagnola | **47** | Larisa | Dora | **78** | Magnisia | Fedora 17 |
| **20** | Trikala | Futura 75 | **48** | Larisa | Fututra 75 | **79** | Αrkadia | Compolti |
| **21** | Trikala | Fedora 17 | **49** | Larisa | Fututra 75 | **80** | Αrkadia | Carmagnola |
| **22** | Magnisia | Futura 75 | **50** | Larisa | Futura 75 | **81** | Messinia | Finola |
| **23** | Magnisia | Futura 75 | **51** | Larisa | Carmagnola | **82** | Larisa | Dora |
| **24** | Evoia | Futura 75 | **52** | Hleia | Carmagnola | **83** | Larisa | Fututra 75 |
| **25** | Magnisia | Futura 75 | **53** | Trikala | Fututra 75 | **84** | Messinia | CS |
| **26** | Hrakleio | Fedora 17 | **54** | Trikala | Futura 75 | **85** | Αrkadia | Compolti |
| **27** | Trikala | Futura 75 | **55** | Hrakleio | Finola | **86** | Magnisia | Finola |
| **28** | Trikala | Fedora 17 | **56** | Trikala | Futura 75 | **87** | Magnisia | Fedora 17 |
|  |  |  | **57** | Messinia | Finola | **88** | Magnisia | Finola |
|  |  |  | **58** | Magnisia | Futura 75 | **89** | Karditsa | CS |
|  |  |  | **59** | Magnisia | Fedora 17 | **90** | Hrakleio | Finola |

**Table S2**: Quantification parameters of trace and macro elements.

| **Element** | **Symbol** | **Monitored isotopes** | **Reporting isotope** | **Interference correction** | **ISTD** | **Analysis mode** | **LOQ**  **(mg/kg)** | **Uncertainty, u’ (%)** |
| --- | --- | --- | --- | --- | --- | --- | --- | --- |
| Boron | *Β* | *11* | *11* |  | *^6^Li* | KED | 2.5 * 10^-3^ | 5.74 |
| Sodium | *Na* | *23* | *23* |  | *^45^Sc* | KED | 0.5 | 5.97 |
| Magnesium | *Mg* | *24* | *24* |  | *^45^Sc* | KED | 0.5 | 4.47 |
| Aluminium | *Al* | *27* | *27* |  | *^45^Sc* | KED | 1.25* 10^-3^ | 5.23 |
| Phosphorus | *P* | *31* | *31* |  | *^45^Sc* | KED | 0.005 | 4.96 |
| Potassium | *K* | *39* | *39* |  | *^45^Sc* | KED | 0.005 | 3.50 |
| Calcium | *Ca* | *43, 44* | *44* |  | *^45^Sc* | KED | 0.5 | 4.17 |
| Titanium | *Ti* | *47,49* | *47* |  | *^45^Sc* | KED | 0.125 * 10^-3^ | 7.08 |
| Vanadium | *V* | *51* | *51* |  | *^45^Sc* | KED | 0.125 * 10^-3^ | 5.55 |
| Chromium | *Cr* | *52, 53* | *52* |  | *^72^Ge* | KED | 0.125 * 10^-3^ | 7.17 |
| Manganese | *Mn* | *55* | *55* |  | *^72^Ge* | KED | 0.5 * 10^-3^ | 7.55 |
| Ferrum | *Fe* | *56, 57* | *56* |  | *^72^Ge* | KED | 1.25* 10^-3^ | 2.98 |
| Cobalt | *Co* | *59* | *59* |  | *^72^Ge* | KED | 0.125 * 10^-3^ | 6.56 |
| Nickel | *Ni* | *60* | *60* |  | *^72^Ge* | KED | 0.125 * 10^-3^ | 5.02 |
| Copper | *Cu* | *63, 65* | *63* |  | *^72^Ge* | KED | 1.25 * 10^-3^ | 2.72 |
| Zinc | *Zn* | *64, 66, 67* | *66* | *-0,0348659*60Ni (KED)* | *^72^Ge* | KED | 1.25 * 10^-3^ | 9.36 |
| Arsenic | *As* | *75* | *75* |  | *^72^Ge* | KED | 0.05 * 10^-3^ | 4.27 |
| Selenium | *Se* | *77, 78* | *78* | *-0304348*83Kr (KED)* | *^72^Ge* | KED | 0.05 * 10^-3^ | 4.41 |
| Strontium | *Sr* | *86, 88* | *88* | *-1,50435*83Kr (KED)* | *^89^Υ* | KED | 2.5* 10^-3^ | 8.64 |
| Molybdenum | *Mo* | *95* | *95* |  | *^89^Υ* | KED | 0.125 * 10^-3^ | 6.09 |
| Silver | *Ag* | *107, 109* | *107* |  | *^115^In* | KED | 0.05 * 10^-3^ | 5.32 |
| Cadmium | *Cd* | *111, 114* | *111* | *-0,0268373*118Sn (KED)* | *^115^In* | KED | 0.05 * 10^-3^ | 4.96 |
| Tin | *Sn* | *118* | *118* |  | *^115^In* | KED | 0.005 * 10^-3^ | 5.06 |
| Antimony | *Sb* | *121* | *121* |  | *^159^Tb* | KED | 0.005 * 10^-3^ | 9.68 |
| Barium | *Ba* | *137* | *137* |  | *^159^Tb* | KED | 1.25 * 10^-3^ | 9.13 |
| Mercury | *Hg* | *200, 201, 202* | *202* |  | *^191^Ir* | KED | 0.01 * 10^-3^ | 3.38 |
| Thallium | *Tl* | *203, 205* | *205* |  | *^191^Ir* | KED | 0.05 * 10^-3^ | 4.26 |
| Lead | *Pb* | *206, 207, 208* | *208* | *1*207Pb (KED) + 1*206Pb (KED)* | *^191^Ir* | KED | 0.05 * 10^-3^ | 5.12 |
| Uranium | *U* | *238* | *238* |  | *^191^Ir* | KED | 0.05 * 10^-3^ | 5.06 |
| **Internal standards** | **Symbol** | **Monitored isotopes** | **Reporting isotope** | **Interference correction** |  |  |  |  |
|  | *Li* | *6* | *6* |  |  |  |  |  |
|  | *Sc* | *45* | *45* |  |  |  |  |  |
|  | *Ge* | *72,73* | *73* |  |  |  |  |  |
|  | *Y* | *89* | *89* |  |  |  |  |  |
|  | *In* | *115* | *115* | *-0.0148637*118Sn (KED)* |  |  |  |  |
|  | *Tb* | *159* | *159* |  |  |  |  |  |
|  | *Ir* | *191,193* | *191* |  |  |  |  |  |

**Table S3.** Elements detected concentrations (mg/kg) in the leaves/flowers of all the cannabis samples.

| **macro elements*** | | | | | | **trace elements**** | | | | | | | | | | |
| --- | --- | --- | --- | --- | --- | --- | --- | --- | --- | --- | --- | --- | --- | --- | --- | --- |
| **No of sample** | **Na** | **Mg** | **P** | **K** | **Ca** | **B** | **Al** | **Ti** | **V** | **Cr** | **Mn** | **Fe** | **Co** | **Ni** | **Cu** |  |
| **1** | 112 | 13510 | 5366 | 23944 | 97351 | 221.568 | 469.658 | 16.058 | 0.840 | 2.275 | 80.418 | 423.758 | 0.343 | 13.276 | 14.843 |  |
| **2** | 99 | 13518 | 10148 | 26521 | 55205 | 119.653 | 413.111 | 25.542 | 0.978 | 1.503 | 226.745 | 559.937 | 0.405 | 2.447 | 35.254 |  |
| **3** | 86 | 18541 | 6138 | 25231 | 108104 | 316.216 | 834.183 | 22.365 | 1.581 | 4.268 | 103.752 | 710.193 | 0.637 | 12.356 | 16.443 |  |
| **4** | 391 | 17434 | 9174 | 28068 | 105853 | 178.164 | 326.815 | 8.725 | 0.543 | 0.726 | 320.130 | 399.733 | 0.473 | 7.076 | 24.492 |  |
| **5** | 85 | 15848 | 6566 | 26411 | 101595 | 244.936 | 733.729 | 25.653 | 1.433 | 4.098 | 95.806 | 658.272 | 1.035 | 9.657 | 19.882 |  |
| **6** | 114 | 10264 | 2652 | 24187 | 102841 | 156.728 | 669.553 | 21.273 | 1.165 | 1.052 | 184.063 | 466.113 | 0.257 | 3.604 | 12.873 |  |
| **7** | 55 | 8758 | 6266 | 30766 | 69748 | 137.746 | 183.798 | 9.407 | 0.337 | 0.710 | 152.505 | 318.415 | 0.149 | 4.269 | 15.716 |  |
| **8** | 232 | 11801 | 4552 | 21948 | 78313 | 126.227 | 294.689 | 8.250 | 0.589 | 0.868 | 196.644 | 275.879 | 0.261 | 5.790 | 10.921 |  |
| **9** | 174 | 19847 | 9194 | 37024 | 121172 | 313.601 | 306.779 | 7.569 | 0.562 | 0.906 | 518.761 | 412.338 | 0.599 | 17.095 | 20.851 |  |
| **10** | 127 | 9636 | 3346 | 26619 | 100453 | 139.842 | 782.559 | 18.712 | 1.500 | 2.586 | 251.164 | 553.925 | 0.583 | 13.475 | 13.711 |  |
| **11** | 536 | 14948 | 7797 | 26776 | 126868 | 178.647 | 198.861 | 6.460 | 0.402 | 2.008 | 174.647 | 275.400 | 0.164 | 4.665 | 18.982 |  |
| **12** | 184 | 9720 | 6574 | 38183 | 88333 | 175.906 | 301.555 | 11.545 | 0.521 | 0.982 | 194.267 | 404.709 | 0.201 | 5.279 | 15.069 |  |
| **13** | 31 | 131722 | 7229 | 15281 | 24031 | 71.865 | 131.448 | 5.730 | 0.317 | 0.824 | 181.653 | 239.291 | 0.248 | 4.106 | 19.471 |  |
| **14** | 123 | 13424 | 6079 | 32456 | 119159 | 189.181 | 400.542 | 13.857 | 0.763 | 1.278 | 198.762 | 395.723 | 0.284 | 11.591 | 20.268 |  |
| **15** | 116 | 11408 | 2018 | 19623 | 52637 | 142.275 | 291.751 | 8.380 | 0.487 | 0.603 | 391.206 | 270.394 | 0.120 | 7.024 | 11.477 |  |
| **16** | 390 | 12809 | 6749 | 28088 | 74944 | 239.395 | 175.767 | 5.690 | 0.349 | 0.549 | 143.296 | 233.757 | 0.106 | 2.231 | 11.547 |  |
| **17** | 372 | 12944 | 6478 | 25732 | 75365 | 183.431 | 291.423 | 7.407 | 0.616 | 1.117 | 127.302 | 412.236 | 0.257 | 6.691 | 24.467 |  |
| **18** | 324 | 13038 | 3711 | 22426 | 88396 | 303.613 | 230.189 | 6.186 | 0.449 | 0.757 | 132.297 | 247.854 | 0.147 | 4.858 | 10.678 |  |
| **19** | 59 | 16838 | 8114 | 26113 | 85252 | 196.351 | 217.163 | 11.418 | 0.478 | 1.403 | 168.613 | 330.174 | 0.251 | 10.622 | 13.273 |  |
| **20** | 36 | 14575 | 4772 | 16350 | 62588 | 173.271 | 133.700 | 7.258 | 0.274 | 0.869 | 116.089 | 209.376 | 0.174 | 12.472 | 9.835 |  |
| **21** | 165 | 13130 | 7016 | 27284 | 72600 | 177.577 | 637.630 | 31.993 | 1.492 | 4.206 | 125.360 | 691.021 | 0.537 | 11.939 | 26.837 |  |
| **22** | 136 | 10505 | 3594 | 13459 | 68467 | 108.027 | 1179.160 | 61.154 | 2.699 | 5.078 | 311.079 | 919.371 | 0.546 | 10.654 | 8.193 |  |
| **23** | 755 | 11563 | 3984 | 18379 | 55573 | 131.416 | 196.293 | 11.397 | 0.425 | 1.990 | 146.262 | 361.031 | 0.366 | 12.048 | 12.091 |  |
| **24** | 310 | 10838 | 8822 | 17072 | 56074 | 121.429 | 364.044 | 18.376 | 0.753 | 1.403 | 215.291 | 522.640 | 0.255 | 6.554 | 25.220 |  |
| **25** | 1672 | 10342 | 6477 | 22859 | 138378 | 141.554 | 846.616 | 30.381 | 2.910 | 3.682 | 447.318 | 693.286 | 0.774 | 10.490 | 24.577 |  |
| **26** | 42 | 11158 | 6713 | 16675 | 51377 | 137.687 | 163.747 | 8.708 | 0.319 | 0.896 | 111.902 | 248.682 | 0.223 | 10.619 | 9.822 |  |
| **27** | 65 | 14907 | 5860 | 16850 | 71849 | 198.719 | 374.322 | 21.569 | 0.863 | 2.127 | 165.640 | 571.201 | 0.446 | 17.057 | 11.489 |  |
| **28** | 45 | 19348 | 4837 | 27604 | 49235 | 292.984 | 227.353 | 12.602 | 0.552 | 1.478 | 162.254 | 286.084 | 0.179 | 4.702 | 17.112 |  |
| **29** | 47 | 14639 | 4334 | 26620 | 42630 | 106.828 | 73.168 | 3.873 | 0.161 | 0.757 | 141.035 | 190.211 | 0.123 | 21.673 | 17.556 |  |
| **30** | 40 | 13551 | 4246 | 28983 | 57984 | 127.445 | 113.721 | 6.802 | 0.238 | 0.628 | 172.048 | 229.476 | 0.169 | 14.669 | 15.833 |  |
| **31** | 35 | 10288 | 4395 | 25269 | 39481 | 75.819 | 99.435 | 6.255 | 0.208 | 0.677 | 113.732 | 181.160 | 0.146 | 11.332 | 12.223 |  |
| **32** | 126 | 17478 | 12245 | 54393 | 81989 | 232.162 | 154.798 | 7.911 | 0.339 | 1.041 | 242.588 | 389.219 | 0.226 | 22.482 | 33.565 |  |
| **33** | 523 | 12787 | 4944 | 27713 | 56593 | 203.040 | 138.768 | 5.707 | 0.354 | 1.025 | 161.292 | 250.616 | 0.131 | 6.356 | 19.203 |  |
| **34** | 106 | 13328 | 4981 | 22938 | 36024 | 147.029 | 75.238 | 4.780 | 0.163 | 0.337 | 90.686 | 135.616 | 0.070 | 3.358 | 17.933 |  |
| **35** | 48 | 9975 | 4027 | 30803 | 64537 | 152.716 | 167.539 | 8.914 | 0.364 | 0.874 | 220.833 | 302.316 | 0.116 | 2.590 | 16.908 |  |
| **36** | 70 | 9880 | 3207 | 19205 | 54359 | 122.780 | 129.660 | 7.046 | 0.227 | 0.542 | 182.635 | 303.549 | 0.134 | 3.651 | 15.591 |  |
| **37** | 140 | 20202 | 8173 | 34039 | 57144 | 306.596 | 282.263 | 8.420 | 0.415 | 0.731 | 148.519 | 365.102 | 1.053 | 20.644 | 33.225 |  |
| **38** | 59 | 10013 | 4488 | 18572 | 45917 | 119.329 | 176.773 | 8.971 | 0.375 | 0.802 | 125.679 | 417.916 | 0.145 | 2.302 | 14.250 |  |
| **39** | 28 | 12301 | 3051 | 22534 | 74728 | 153.442 | 131.811 | 7.878 | 0.264 | 1.126 | 190.786 | 216.147 | 0.176 | 9.066 | 10.737 |  |
| **40** | 46 | 11951 | 4506 | 26149 | 83514 | 159.954 | 115.207 | 5.526 | 0.232 | 0.578 | 165.204 | 219.996 | 0.158 | 4.415 | 12.784 |  |
| **41** | 82 | 12420 | 3719 | 17509 | 40681 | 197.580 | 135.256 | 7.473 | 0.343 | 0.572 | 162.281 | 194.249 | 0.158 | 3.571 | 12.357 |  |
| **42** | 36 | 10012 | 2816 | 23568 | 59846 | 174.656 | 241.939 | 12.794 | 0.507 | 1.026 | 163.521 | 284.048 | 0.150 | 2.538 | 12.490 |  |
| **43** | 30 | 10512 | 5477 | 17009 | 35543 | 122.846 | 254.705 | 13.012 | 0.558 | 1.482 | 118.532 | 331.693 | 0.343 | 17.015 | 14.300 |  |
| **44** | 36 | 10681 | 7135 | 21570 | 47199 | 124.911 | 269.174 | 15.152 | 0.577 | 1.312 | 147.294 | 390.177 | 0.333 | 15.845 | 14.743 |  |
| **45** | 70 | 10124 | 2590 | 21318 | 67719 | 199.949 | 357.034 | 18.628 | 0.819 | 1.455 | 144.716 | 384.133 | 0.218 | 2.936 | 11.996 |  |
| **46** | 60 | 12515 | 3267 | 19053 | 69168 | 184.626 | 254.665 | 14.393 | 0.643 | 1.047 | 151.746 | 258.848 | 0.212 | 5.370 | 11.834 |  |
| **47** | 46 | 13623 | 4172 | 23500 | 54130 | 191.240 | 180.031 | 10.106 | 0.388 | 0.926 | 177.867 | 272.284 | 0.171 | 4.879 | 17.163 |  |
| **48** | 62 | 14302 | 4855 | 21183 | 73099 | 227.450 | 272.936 | 15.228 | 0.615 | 1.305 | 179.387 | 316.622 | 0.257 | 6.165 | 15.287 |  |
| **49** | 35 | 11230 | 2562 | 22675 | 57163 | 209.747 | 416.504 | 21.325 | 0.869 | 1.637 | 170.427 | 402.691 | 0.213 | 4.034 | 18.205 |  |
| **50** | 52 | 9916 | 7327 | 29935 | 54127 | 175.301 | 339.914 | 30.378 | 0.861 | 2.148 | 165.322 | 421.770 | 0.276 | 3.552 | 16.968 |  |
| **51** | 80 | 11996 | 5007 | 30244 | 62292 | 207.229 | 139.796 | 5.010 | 0.274 | 0.536 | 120.506 | 256.292 | 0.106 | 2.942 | 23.934 |  |
| **52** | 45 | 10760 | 6439 | 22726 | 65356 | 126.570 | 393.900 | 24.082 | 0.935 | 2.489 | 146.059 | 456.121 | 0.396 | 9.703 | 16.326 |  |
| **53** | 35 | 10409 | 7110 | 24090 | 50496 | 111.276 | 423.873 | 24.274 | 0.902 | 1.929 | 168.536 | 493.495 | 0.494 | 15.516 | 18.297 |  |
| **54** | 461 | 11074 | 7096 | 24094 | 93507 | 154.212 | 201.727 | 8.218 | 0.947 | 0.901 | 231.800 | 231.794 | 0.451 | 4.564 | 20.112 |  |
| **55** | 38 | 16510 | 3800 | 23685 | 88672 | 209.003 | 152.810 | 9.011 | 0.331 | 1.320 | 232.561 | 290.695 | 0.205 | 11.156 | 12.000 |  |
| **56** | 660 | 10858 | 3913 | 28380 | 87209 | 145.173 | 270.549 | 6.633 | 0.535 | 1.133 | 229.893 | 395.354 | 0.340 | 4.884 | 64.222 |  |
| **57** | 58 | 10318 | 2256 | 24050 | 61850 | 186.486 | 79.610 | 4.367 | 0.160 | 0.494 | 116.408 | 162.430 | 0.160 | 6.098 | 15.476 |  |
| **58** | 93 | 11682 | 4708 | 19096 | 69577 | 220.292 | 138.069 | 6.418 | 0.295 | 0.806 | 178.744 | 217.561 | 0.387 | 14.288 | 14.455 |  |
| **59** | 219 | 11394 | 3441 | 37565 | 115211 | 468.974 | 562.013 | 18.091 | 1.048 | 2.463 | 183.169 | 471.908 | 0.317 | 4.526 | 19.228 |  |
| **60** | 62 | 13614 | 6380 | 25895 | 53389 | 250.771 | 496.427 | 32.620 | 1.506 | 2.132 | 141.120 | 482.826 | 0.357 | 5.260 | 22.784 |  |
| **61** | 106 | 14682 | 7435 | 34921 | 123176 | 223.907 | 524.977 | 17.846 | 1.043 | 1.732 | 444.601 | 543.992 | 0.379 | 4.054 | 19.266 |  |
| **62** | 1001 | 10292 | 2366 | 16484 | 48382 | 145.457 | 400.296 | 23.089 | 0.909 | 3.281 | 110.398 | 457.568 | 0.461 | 11.131 | 8.900 |  |
| **63** | 879 | 9528 | 2252 | 15451 | 41132 | 143.647 | 360.298 | 19.716 | 0.837 | 2.725 | 91.453 | 397.605 | 0.408 | 8.524 | 8.470 |  |
| **64** | 149 | 15998 | 5467 | 20879 | 63234 | 316.722 | 361.477 | 19.274 | 1.045 | 2.608 | 184.379 | 388.071 | 0.366 | 5.425 | 33.202 |  |
| **65** | 82 | 14059 | 4941 | 17962 | 45647 | 230.829 | 179.263 | 9.201 | 0.656 | 1.274 | 129.981 | 271.633 | 0.194 | 4.213 | 26.316 |  |
| **66** | 106 | 11843 | 4344 | 16325 | 47086 | 228.864 | 143.494 | 7.017 | 0.619 | 1.043 | 128.480 | 234.640 | 0.193 | 4.880 | 45.012 |  |
| **67** | 118 | 14176 | 7163 | 26122 | 45221 | 154.096 | 353.356 | 17.327 | 0.758 | 1.509 | 120.781 | 399.606 | 0.547 | 24.397 | 17.718 |  |
| **68** | 78 | 7140 | 6014 | 17206 | 30086 | 71.389 | 467.474 | 23.536 | 0.982 | 1.860 | 78.138 | 422.801 | 0.386 | 9.711 | 14.706 |  |
| **69** | 83 | 10291 | 7162 | 19317 | 40259 | 152.288 | 370.405 | 21.146 | 0.871 | 1.576 | 89.469 | 373.063 | 0.440 | 11.360 | 11.951 |  |
| **70** | 143 | 11078 | 6793 | 19373 | 43640 | 142.182 | 926.245 | 53.364 | 2.183 | 5.050 | 139.957 | 879.845 | 1.073 | 31.071 | 14.497 |  |
| **71** | 113 | 15560 | 8807 | 26922 | 60922 | 200.521 | 1352.904 | 73.696 | 3.226 | 7.886 | 197.213 | 1338.452 | 1.608 | 48.958 | 20.503 |  |
| **72** | 60 | 10892 | 7475 | 27052 | 64548 | 164.328 | 316.471 | 16.977 | 0.680 | 1.780 | 124.015 | 382.692 | 0.504 | 12.590 | 25.097 |  |
| **73** | 114 | 13939 | 7149 | 17242 | 42785 | 177.467 | 467.749 | 22.569 | 1.051 | 2.563 | 247.284 | 455.895 | 0.466 | 8.321 | 20.297 |  |
| **74** | 88 | 11343 | 5924 | 21008 | 54694 | 155.832 | 438.352 | 14.707 | 0.923 | 3.009 | 148.084 | 404.065 | 0.480 | 7.726 | 12.368 |  |
| **75** | 165 | 9120 | 5640 | 14116 | 38241 | 102.923 | 328.849 | 13.095 | 0.623 | 0.861 | 88.932 | 314.027 | 0.169 | 4.104 | 14.477 |  |
| **76** | 170 | 10194 | 6214 | 15811 | 55329 | 142.888 | 363.558 | 14.270 | 0.706 | 0.943 | 110.821 | 370.270 | 0.188 | 11.192 | 13.032 |  |
| **77** | 701 | 18815 | 6454 | 20077 | 85927 | 379.661 | 554.310 | 23.532 | 1.282 | 2.858 | 300.278 | 533.370 | 0.560 | 8.109 | 26.424 |  |
| **78** | 122 | 11977 | 8383 | 21162 | 78338 | 214.290 | 430.276 | 14.717 | 0.795 | 1.281 | 269.084 | 484.189 | 0.277 | 7.473 | 20.644 |  |
| **79** | 140 | 12591 | 9433 | 23969 | 99851 | 245.549 | 627.650 | 24.254 | 1.196 | 1.696 | 277.615 | 627.948 | 0.325 | 6.073 | 23.407 |  |
| **80** | 146 | 12356 | 10608 | 26745 | 83155 | 256.093 | 481.403 | 19.771 | 0.979 | 1.349 | 300.793 | 505.352 | 0.296 | 7.254 | 19.095 |  |
| **81** | 366 | 14874 | 5255 | 23367 | 100500 | 162.342 | 366.086 | 8.477 | 0.686 | 1.247 | 76.876 | 436.722 | 0.242 | 3.557 | 19.554 |  |
| **82** | 117 | 16354 | 5998 | 18251 | 87300 | 197.494 | 472.074 | 22.747 | 1.043 | 2.141 | 221.863 | 394.093 | 0.667 | 7.454 | 14.683 |  |
| **83** | 171 | 17226 | 8750 | 24681 | 50541 | 195.790 | 408.799 | 20.241 | 0.900 | 1.916 | 361.607 | 434.109 | 0.551 | 9.508 | 22.460 |  |
| **84** | 248 | 11442 | 15088 | 28425 | 59840 | 157.836 | 377.059 | 13.449 | 1.010 | 1.266 | 393.219 | 400.854 | 0.260 | 1.759 | 18.504 |  |
| **85** | 82 | 11322 | 5032 | 15168 | 42779 | 102.562 | 380.104 | 10.133 | 0.818 | 1.296 | 251.901 | 388.131 | 0.201 | 4.072 | 30.758 |  |
| **86** | 65 | 10488 | 9320 | 33809 | 68029 | 88.825 | 41.836 | 1.823 | 0.068 | 0.364 | 275.674 | 247.241 | 0.107 | 2.320 | 14.929 |  |
| **87** | 126 | 14934 | 10153 | 25064 | 37775 | 233.567 | 919.778 | 40.297 | 2.051 | 4.747 | 433.455 | 954.364 | 0.662 | 6.216 | 21.793 |  |
| **88** | 126 | 19502 | 12107 | 29373 | 67258 | 146.357 | 91.115 | 4.120 | 0.207 | 1.318 | 445.927 | 365.285 | 0.091 | 3.897 | 10.967 |  |
| **89** | 88 | 14455 | 10833 | 23471 | 64894 | 290.776 | 385.396 | 23.695 | 1.195 | 1.968 | 262.912 | 423.848 | 0.423 | 5.313 | 38.722 |  |
| **90** | 582 | 12292 | 9258 | 23050 | 96433 | 144.974 | 256.086 | 10.189 | 1.114 | 2.080 | 363.022 | 276.487 | 0.536 | 5.421 | 26.582 |  |

| **trace elements**** | | | | | | | | | | | | | | |
| --- | --- | --- | --- | --- | --- | --- | --- | --- | --- | --- | --- | --- | --- | --- |
|  | **Zn** | **As** | **Se** | **Sr** | **Mo** | **Ag** | **Cd** | **Sn** | **Sb** | **Ba** | **Hg** | **Tl** | **Pb** | **U** |
| **1** | 42.880 | 0.175 | 0.082 | 159.382 | 2.471 | 0.142 | 0.054 | 0.082 | 0.019 | 52.902 | 0.013 | 0.016 | 0.401 | 0.021 |
| **2** | 82.379 | 0.742 | 0.089 | 251.225 | 4.483 | 0.079 | 0.019 | 0.116 | 0.107 | 53.372 | 0.020 | 0.013 | 1.290 | 0.029 |
| **3** | 47.542 | 0.249 | 0.091 | 189.568 | 2.267 | 0.054 | 0.043 | 0.060 | 0.028 | 58.716 | 0.011 | 0.020 | 0.652 | 0.025 |
| **4** | 98.819 | 0.161 | 0.099 | 348.403 | 3.468 | 0.051 | 0.036 | 0.059 | 0.009 | 30.281 | 0.015 | 0.069 | 0.437 | 0.034 |
| **5** | 50.721 | 0.232 | 0.075 | 171.524 | 2.831 | 0.022 | 0.028 | 0.065 | 0.030 | 54.362 | 0.008 | 0.018 | 0.597 | 0.022 |
| **6** | 49.583 | 0.195 | 0.049 | 93.402 | 1.907 | 0.026 | 0.019 | 0.059 | 0.013 | 31.297 | 0.010 | 0.026 | 0.627 | 0.023 |
| **7** | 51.801 | 0.105 | 0.082 | 59.911 | 1.780 | 0.026 | 0.023 | 0.041 | 0.004 | 16.145 | 0.022 | 0.024 | 0.190 | 0.007 |
| **8** | 51.050 | 0.132 | 0.086 | 179.440 | 1.051 | 0.032 | 0.093 | 0.045 | 0.013 | 76.675 | 0.013 | 0.439 | 0.305 | 0.020 |
| **9** | 93.746 | 0.252 | 0.405 | 222.302 | 1.392 | 0.080 | 0.072 | 0.065 | 0.017 | 223.648 | 0.067 | 0.104 | 0.658 | 0.014 |
| **10** | 58.789 | 0.135 | 0.071 | 173.367 | 1.020 | 0.021 | 0.025 | 0.056 | 0.012 | 29.201 | 0.020 | 0.106 | 0.453 | 0.016 |
| **11** | 83.461 | 0.117 | 0.117 | 349.477 | 2.679 | 0.040 | 0.058 | 0.046 | 0.019 | 82.189 | 0.021 | 0.362 | 0.324 | 0.025 |
| **12** | 50.246 | 0.124 | 0.052 | 119.363 | 6.268 | 0.131 | 0.079 | 0.045 | 0.008 | 60.314 | 0.016 | 0.014 | 0.316 | 0.015 |
| **13** | 56.898 | 0.132 | 0.247 | 89.198 | 3.471 | 0.045 | 0.015 | 0.030 | 0.011 | 17.443 | 0.008 | 0.018 | 0.154 | 0.005 |
| **14** | 109.118 | 0.145 | 0.108 | 434.426 | 12.947 | 0.063 | 0.065 | 0.071 | 0.018 | 25.836 | 0.035 | 0.322 | 0.398 | 0.021 |
| **15** | 51.024 | 0.291 | 0.489 | 91.672 | 0.231 | 0.053 | 0.076 | 0.047 | 0.026 | 107.837 | 0.014 | 0.911 | 0.387 | 0.010 |
| **16** | 63.503 | 0.085 | 0.064 | 292.270 | 1.039 | 0.027 | 0.018 | 0.041 | 0.009 | 30.402 | 0.011 | 0.044 | 0.240 | 0.023 |
| **17** | 113.589 | 0.104 | 0.060 | 366.579 | 3.487 | 0.046 | 0.019 | 0.063 | 0.016 | 16.565 | 0.021 | 0.181 | 0.323 | 0.035 |
| **18** | 62.700 | 0.090 | 0.062 | 366.683 | 1.121 | 0.034 | 0.026 | 0.043 | 0.011 | 36.822 | 0.015 | 0.031 | 0.244 | 0.017 |
| **19** | 75.950 | 0.072 | 0.052 | 180.381 | 1.105 | 0.074 | 0.031 | 0.050 | 0.017 | 42.924 | 0.080 | 0.130 | 0.735 | 0.016 |
| **20** | 61.569 | 0.052 | 0.050 | 124.965 | 0.400 | 0.070 | 0.034 | 0.049 | 0.014 | 29.040 | 0.085 | 0.092 | 0.243 | 0.009 |
| **21** | 96.487 | 0.439 | 0.108 | 107.944 | 1.622 | 0.060 | 0.113 | 0.086 | 0.033 | 32.320 | 0.068 | 0.141 | 0.637 | 0.024 |
| **22** | 34.169 | 0.379 | 0.126 | 145.558 | 0.411 | 0.026 | 0.048 | 0.415 | 0.061 | 70.648 | 0.011 | 0.147 | 1.019 | 0.045 |
| **23** | 79.933 | 0.125 | 0.052 | 51.463 | 0.253 | 0.033 | 0.013 | 0.039 | 0.013 | 11.617 | 0.011 | 0.042 | 0.233 | 0.010 |
| **24** | 96.466 | 0.120 | 0.046 | 70.878 | 1.092 | 0.043 | 0.057 | 0.052 | 0.016 | 20.640 | 0.018 | 0.045 | 0.425 | 0.011 |
| **25** | 86.454 | 0.692 | 0.113 | 181.983 | 2.160 | 0.064 | 0.037 | 0.110 | 0.068 | 42.243 | 0.014 | 0.059 | 0.754 | 0.365 |
| **26** | 62.546 | 0.059 | 0.038 | 111.706 | 0.476 | 0.045 | 0.034 | 0.035 | 0.014 | 27.844 | 0.007 | 0.068 | 0.240 | 0.010 |
| **27** | 125.379 | 0.113 | 0.053 | 151.794 | 0.316 | 0.090 | 0.062 | 0.063 | 0.024 | 40.822 | 0.010 | 0.079 | 0.570 | 0.020 |
| **28** | 52.520 | 0.112 | 0.191 | 384.384 | 3.243 | 0.201 | 0.020 | 0.050 | 0.028 | 72.157 | 0.015 | 0.033 | 0.664 | 0.029 |
| **29** | 62.135 | 0.032 | 0.085 | 90.541 | 0.322 | 0.061 | 0.029 | 0.017 | 0.007 | 16.695 | 0.010 | 0.153 | 0.261 | 0.005 |
| **30** | 46.089 | 0.048 | 0.072 | 120.390 | 0.405 | 0.147 | 0.021 | 0.038 | 0.009 | 19.842 | 0.013 | 0.165 | 0.135 | 0.006 |
| **31** | 39.419 | 0.031 | 0.044 | 71.511 | 0.529 | 0.041 | 0.019 | 0.032 | 0.007 | 11.662 | 0.006 | 0.145 | 0.095 | 0.004 |
| **32** | 102.172 | 0.089 | 0.201 | 117.947 | 1.949 | 0.127 | 0.018 | 0.038 | 0.012 | 37.562 | 0.021 | 0.101 | 0.143 | 0.030 |
| **33** | 44.259 | 0.077 | 0.089 | 297.274 | 2.277 | 0.026 | 0.017 | 0.038 | 0.015 | 67.426 | 0.015 | 0.039 | 0.212 | 0.023 |
| **34** | 30.602 | 0.063 | 0.035 | 201.632 | 2.550 | 0.037 | 0.007 | 0.014 | 0.004 | 21.097 | 0.010 | 0.013 | 0.113 | 0.009 |
| **35** | 53.866 | 0.059 | 0.088 | 123.647 | 1.789 | 0.048 | 0.012 | 0.037 | 0.011 | 25.757 | 0.013 | 0.007 | 0.235 | 0.008 |
| **36** | 44.681 | 0.040 | 0.054 | 111.970 | 0.582 | 0.033 | 0.022 | 0.025 | 0.009 | 22.061 | 0.008 | 0.057 | 0.183 | 0.004 |
| **37** | 87.728 | 0.242 | 0.157 | 203.108 | 1.656 | 0.221 | 0.129 | 0.041 | 0.015 | 38.131 | 0.020 | 0.191 | 0.287 | 0.012 |
| **38** | 44.985 | 0.067 | 0.078 | 171.238 | 1.912 | 0.025 | 0.015 | 0.094 | 0.019 | 16.787 | 0.009 | 0.073 | 0.231 | 0.007 |
| **39** | 31.645 | 0.057 | 0.070 | 129.331 | 0.314 | 0.043 | 0.014 | 0.034 | 0.015 | 19.212 | 0.011 | 0.160 | 0.164 | 0.007 |
| **40** | 31.480 | 0.047 | 0.117 | 220.651 | 1.064 | 0.179 | 0.025 | 0.032 | 0.009 | 28.082 | 0.021 | 0.033 | 0.550 | 0.007 |
| **41** | 23.616 | 0.075 | 0.059 | 310.018 | 2.467 | 0.122 | 0.019 | 0.029 | 0.018 | 40.105 | 0.013 | 0.040 | 0.207 | 0.012 |
| **42** | 25.297 | 0.077 | 0.058 | 270.660 | 1.328 | 0.042 | 0.021 | 0.031 | 0.017 | 52.955 | 0.011 | 0.028 | 0.244 | 0.008 |
| **43** | 67.793 | 0.061 | 0.043 | 64.679 | 0.272 | 0.153 | 0.012 | 0.044 | 0.016 | 12.622 | 0.027 | 0.080 | 0.293 | 0.010 |
| **44** | 73.978 | 0.070 | 0.064 | 95.766 | 0.430 | 0.037 | 0.022 | 0.053 | 0.024 | 21.248 | 0.010 | 0.072 | 0.330 | 0.012 |
| **45** | 54.954 | 0.117 | 0.058 | 114.479 | 1.001 | 0.113 | 0.014 | 0.064 | 0.015 | 63.233 | 0.016 | 0.083 | 0.346 | 0.014 |
| **46** | 63.713 | 0.105 | 0.055 | 373.881 | 1.180 | 0.154 | 0.021 | 0.038 | 0.010 | 98.889 | 0.010 | 0.072 | 0.293 | 0.011 |
| **47** | 23.131 | 0.079 | 1.244 | 267.156 | 2.626 | 0.066 | 0.020 | 0.038 | 0.015 | 37.097 | 0.013 | 0.039 | 0.285 | 0.018 |
| **48** | 66.660 | 0.101 | 0.043 | 333.410 | 1.083 | 0.050 | 0.019 | 0.065 | 0.028 | 104.594 | 0.019 | 0.036 | 0.392 | 0.014 |
| **49** | 54.335 | 0.095 | 0.028 | 193.635 | 0.373 | 0.042 | 0.017 | 0.038 | 0.012 | 41.397 | 0.010 | 0.031 | 0.271 | 0.014 |
| **50** | 68.326 | 0.157 | 0.108 | 111.090 | 1.639 | 0.160 | 0.018 | 0.081 | 0.009 | 13.296 | 0.018 | 0.011 | 0.259 | 0.014 |
| **51** | 91.804 | 0.066 | 0.203 | 243.784 | 2.511 | 0.045 | 0.019 | 0.049 | 0.017 | 9.562 | 0.022 | 0.175 | 0.143 | 0.008 |
| **52** | 74.445 | 0.101 | 0.145 | 90.223 | 0.515 | 0.205 | 0.021 | 0.090 | 0.017 | 17.205 | 0.020 | 0.060 | 0.442 | 0.019 |
| **53** | 85.352 | 0.096 | 0.057 | 108.588 | 0.226 | 0.097 | 0.026 | 0.049 | 0.042 | 31.980 | 0.016 | 0.061 | 0.391 | 0.017 |
| **54** | 53.115 | 0.554 | 0.051 | 186.212 | 2.588 | 0.040 | 0.031 | 0.060 | 0.029 | 32.785 | 0.088 | 0.018 | 0.359 | 0.045 |
| **55** | 42.064 | 0.067 | 0.146 | 180.588 | 0.344 | 0.035 | 0.025 | 0.048 | 0.019 | 29.359 | 0.013 | 0.158 | 0.233 | 0.008 |
| **56** | 106.625 | 0.098 | 0.117 | 192.773 | 0.595 | 0.024 | 0.029 | 0.037 | 0.063 | 31.097 | 0.013 | 0.043 | 0.405 | 0.032 |
| **57** | 36.578 | 0.070 | 0.044 | 44.306 | 0.162 | 0.089 | 0.012 | 0.021 | 0.008 | 37.151 | 0.011 | 0.046 | 0.145 | 0.003 |
| **58** | 37.851 | 0.091 | 0.052 | 53.759 | 0.183 | 0.048 | 0.057 | 0.028 | 0.011 | 48.542 | 0.012 | 0.077 | 0.219 | 0.005 |
| **59** | 78.364 | 0.208 | 0.096 | 332.532 | 0.703 | 0.189 | 0.043 | 0.060 | 0.016 | 38.033 | 0.023 | 0.091 | 0.356 | 0.017 |
| **60** | 78.788 | 0.106 | 0.278 | 110.232 | 2.551 | 0.303 | 0.028 | 0.060 | 0.016 | 23.808 | 0.017 | 0.041 | 0.522 | 0.014 |
| **61** | 73.821 | 0.285 | 0.042 | 266.236 | 2.706 | 0.097 | 0.077 | 0.078 | 0.033 | 25.861 | 0.016 | 0.096 | 1.143 | 0.037 |
| **62** | 51.443 | 0.187 | 0.085 | 37.763 | 0.345 | 0.070 | 0.021 | 0.057 | 0.018 | 9.335 | 0.036 | 0.067 | 0.409 | 0.016 |
| **63** | 49.548 | 0.167 | 0.054 | 35.948 | 0.436 | 0.043 | 0.011 | 0.040 | 0.018 | 9.509 | 0.017 | 0.047 | 0.332 | 0.015 |
| **64** | 80.284 | 0.192 | 0.022 | 195.011 | 1.088 | 0.024 | 0.031 | 0.086 | 0.024 | 35.690 | 0.018 | 0.058 | 0.543 | 0.017 |
| **65** | 56.688 | 0.125 | 0.030 | 133.529 | 1.174 | 0.036 | 0.015 | 0.044 | 0.019 | 22.047 | 0.009 | 0.041 | 0.370 | 0.010 |
| **66** | 55.421 | 0.092 | 0.055 | 156.876 | 0.697 | 0.029 | 0.032 | 0.041 | 0.013 | 25.725 | 0.008 | 0.045 | 0.338 | 0.012 |
| **67** | 61.988 | 0.108 | 0.047 | 74.696 | 0.382 | 0.051 | 0.036 | 0.051 | 0.026 | 21.860 | 0.013 | 0.038 | 0.387 | 0.013 |
| **68** | 51.315 | 0.126 | 0.047 | 56.206 | 0.412 | 0.094 | 0.025 | 0.069 | 0.028 | 15.841 | 0.015 | 0.031 | 0.387 | 0.021 |
| **69** | 52.970 | 0.097 | 0.059 | 81.453 | 0.472 | 0.011 | 0.058 | 0.062 | 0.026 | 22.391 | 0.011 | 0.064 | 0.355 | 0.022 |
| **70** | 61.321 | 0.187 | 0.083 | 71.096 | 0.320 | 0.225 | 0.025 | 0.074 | 0.031 | 17.787 | 0.014 | 0.059 | 0.666 | 0.036 |
| **71** | 79.705 | 0.258 | 0.184 | 101.021 | 0.499 | 0.083 | 0.030 | 0.112 | 0.052 | 22.808 | 0.016 | 0.080 | 1.067 | 0.051 |
| **72** | 71.334 | 0.104 | 0.028 | 133.440 | 0.557 | 0.144 | 0.020 | 0.059 | 0.019 | 28.539 | 0.017 | 0.024 | 1.195 | 0.020 |
| **73** | 42.544 | 0.172 | 0.096 | 348.963 | 1.473 | 0.066 | 0.068 | 0.101 | 0.033 | 47.981 | 0.015 | 0.023 | 0.430 | 0.025 |
| **74** | 62.261 | 0.147 | 0.035 | 171.835 | 0.959 | 0.061 | 0.019 | 0.063 | 0.022 | 77.345 | 0.013 | 0.062 | 0.620 | 0.018 |
| **75** | 50.779 | 0.140 | 0.119 | 70.276 | 0.797 | 0.145 | 0.039 | 0.041 | 0.017 | 16.983 | 0.009 | 0.064 | 0.470 | 0.017 |
| **76** | 64.632 | 0.149 | 0.183 | 102.627 | 0.721 | 0.037 | 0.046 | 0.044 | 0.017 | 27.149 | 0.010 | 0.095 | 0.425 | 0.019 |
| **77** | 102.256 | 0.327 | 0.099 | 100.972 | 0.551 | 0.045 | 0.431 | 0.076 | 0.038 | 48.711 | 0.024 | 0.118 | 0.825 | 0.028 |
| **78** | 81.527 | 0.262 | 0.193 | 93.219 | 5.988 | 0.052 | 0.126 | 0.054 | 0.013 | 49.479 | 0.022 | 0.035 | 0.479 | 0.027 |
| **79** | 78.770 | 0.271 | 0.126 | 111.261 | 5.907 | 0.073 | 0.137 | 0.060 | 0.018 | 52.806 | 0.017 | 0.049 | 0.515 | 0.035 |
| **80** | 78.817 | 0.268 | 0.133 | 94.956 | 8.503 | 0.062 | 0.243 | 0.062 | 0.019 | 61.049 | 0.019 | 0.031 | 0.378 | 0.027 |
| **81** | 65.120 | 0.097 | 0.082 | 1032.403 | 1.885 | 0.065 | 0.168 | 0.038 | 0.014 | 38.191 | 0.107 | 0.156 | 0.331 | 0.026 |
| **82** | 90.313 | 0.172 | 0.050 | 266.001 | 1.211 | 0.025 | 0.030 | 0.062 | 0.026 | 111.165 | 0.013 | 0.066 | 0.694 | 0.020 |
| **83** | 49.557 | 0.182 | 0.080 | 333.143 | 2.500 | 0.041 | 0.185 | 0.068 | 0.031 | 68.533 | 0.017 | 0.027 | 0.429 | 0.021 |
| **84** | 157.894 | 0.173 | 0.056 | 117.246 | 4.457 | 0.170 | 0.059 | 0.049 | 0.024 | 19.586 | 0.012 | 0.025 | 0.399 | 0.018 |
| **85** | 78.352 | 0.093 | 0.097 | 68.519 | 2.480 | 0.168 | 0.023 | 0.062 | 0.023 | 14.792 | 0.012 | 0.103 | 0.390 | 0.050 |
| **86** | 84.636 | 0.081 | 0.073 | 111.541 | 7.717 | 0.179 | 0.045 | 0.031 | 0.011 | 29.060 | 0.020 | 0.033 | 0.165 | 0.002 |
| **87** | 145.012 | 0.340 | 0.195 | 262.589 | 2.601 | 0.266 | 0.109 | 0.181 | 0.093 | 50.828 | 0.035 | 0.047 | 1.752 | 0.073 |
| **88** | 146.092 | 0.120 | 0.209 | 159.605 | 38.110 | 0.059 | 0.179 | 0.049 | 0.697 | 14.665 | 0.013 | 0.052 | 0.425 | 0.004 |
| **89** | 109.401 | 0.087 | 0.102 | 152.998 | 1.769 | 0.065 | 0.041 | 0.050 | 0.024 | 27.924 | 0.015 | 0.024 | 0.346 | 0.015 |
| **90** | 65.533 | 0.430 | 0.044 | 168.907 | 3.416 | 0.034 | 0.032 | 0.060 | 0.042 | 44.858 | 0.012 | 0.027 | 0.411 | 0.057 |

*: Concentrations of macro elements are given with no decimal digits

**: Concentrations of trace elements are given with three decimal digits

**Fig. S1:** Box plots of each element concentration among the sampling areas.


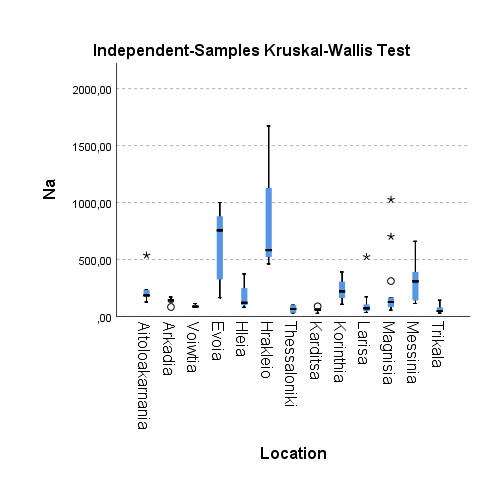

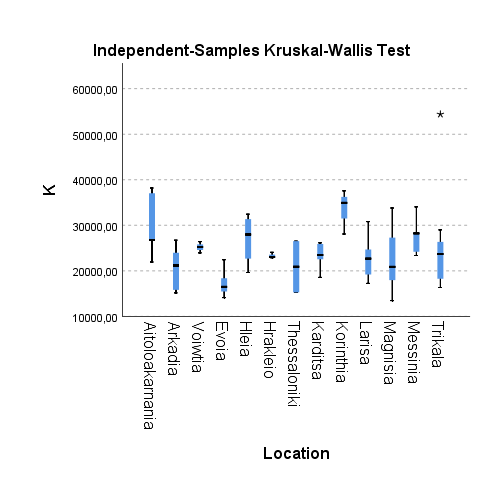

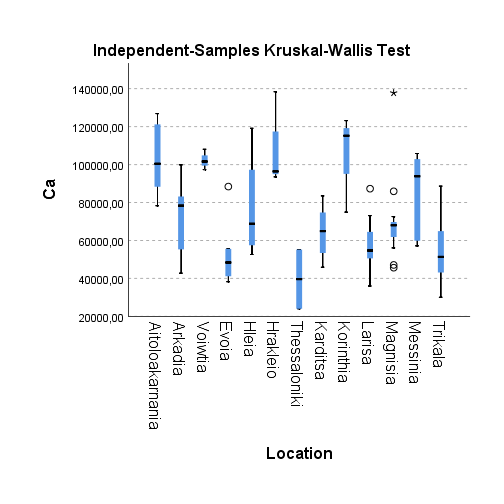


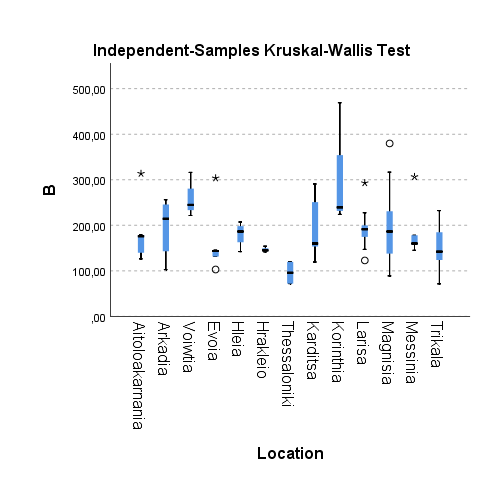

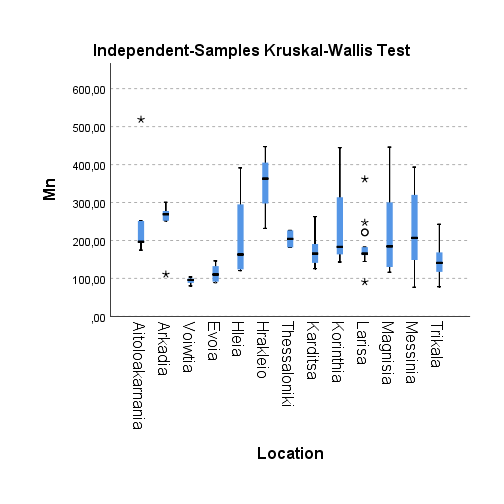

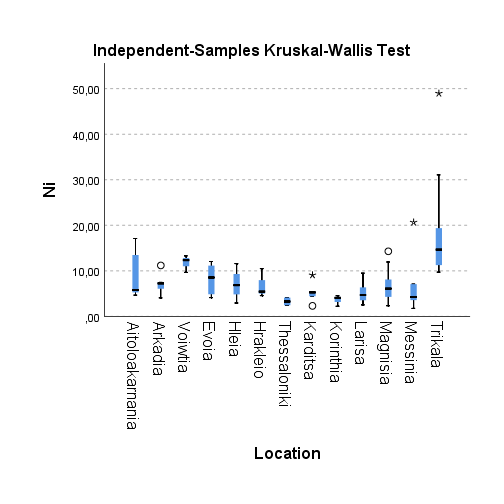


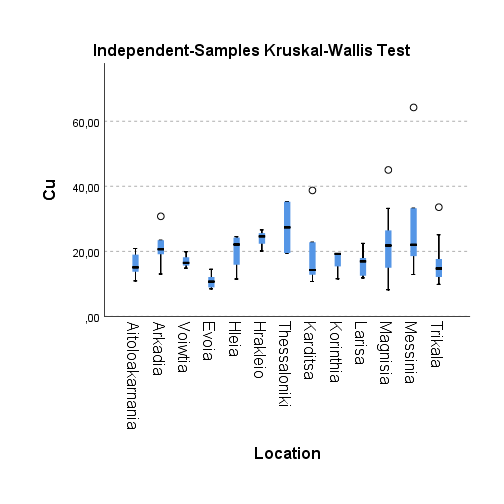

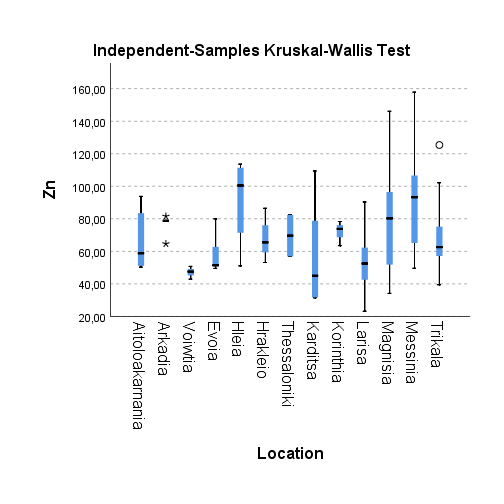

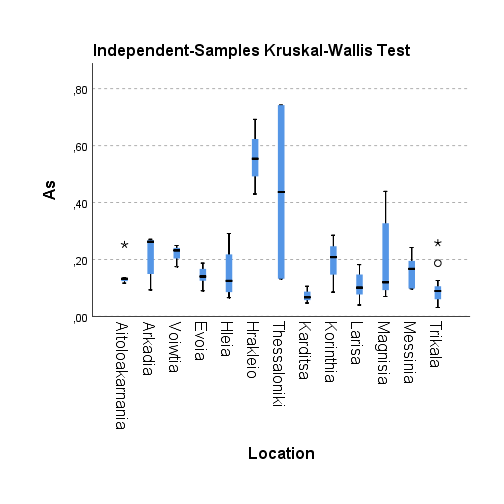

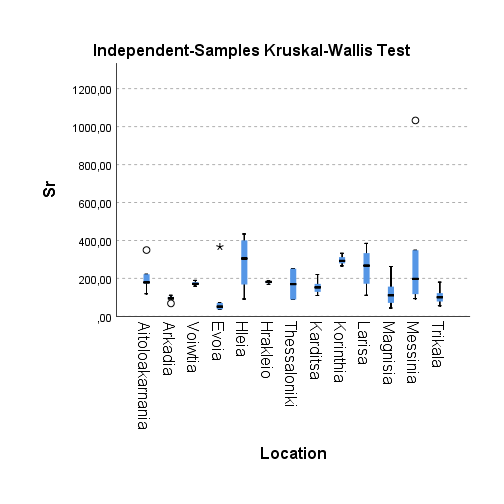

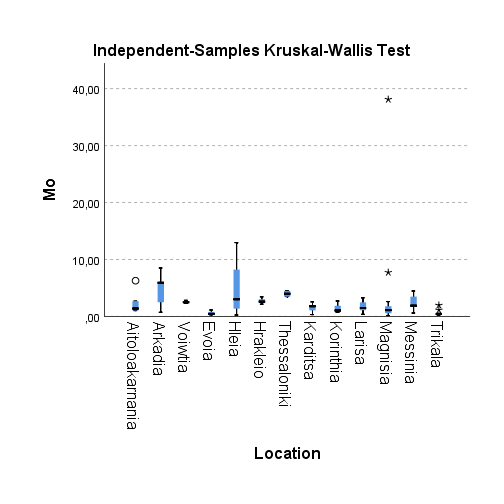

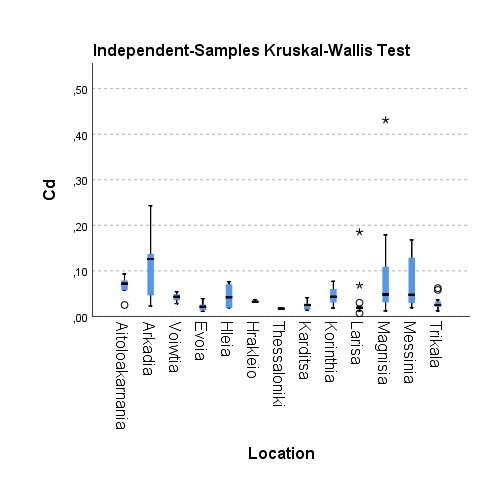


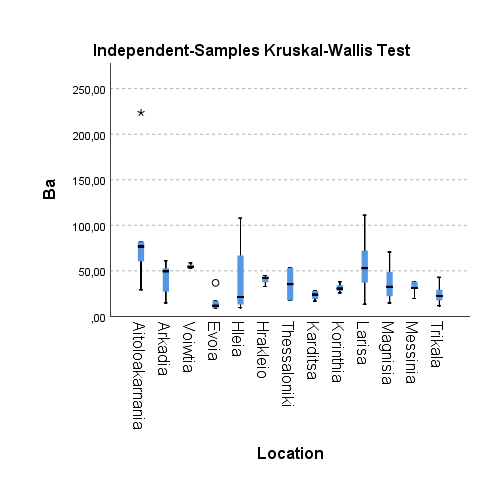

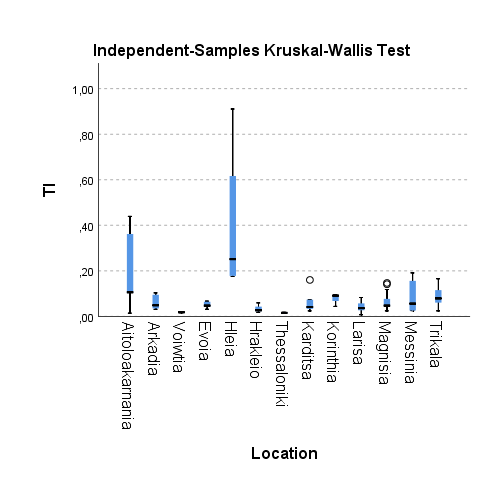

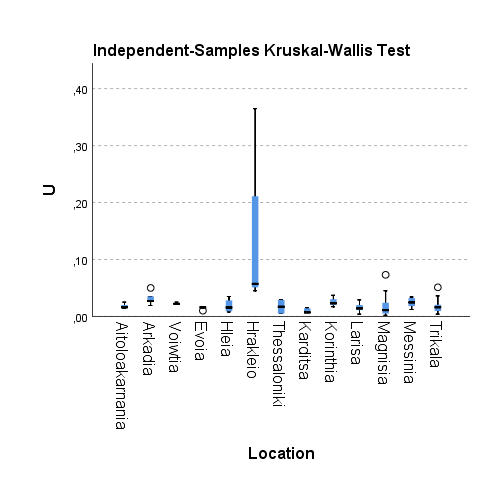


**Fig.S2**: Box plots of each element concentration among the different varieties of cannabis.


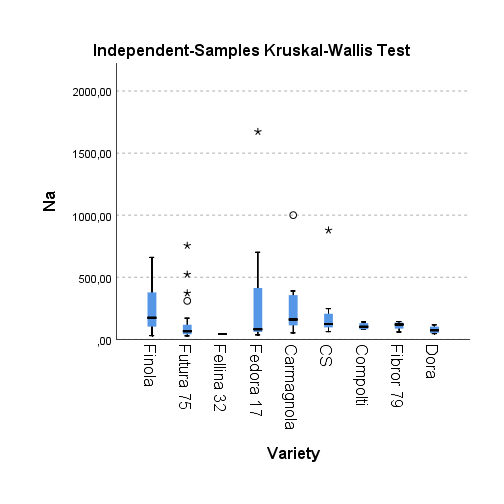

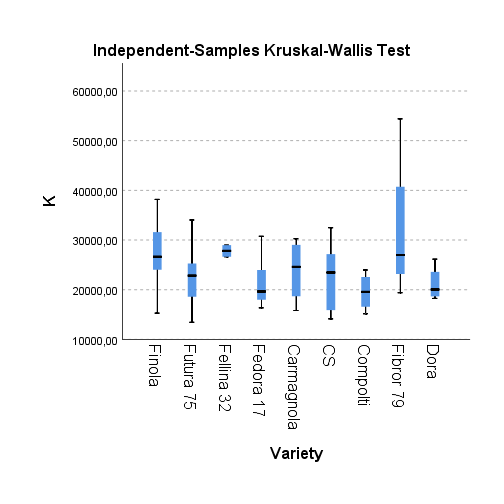

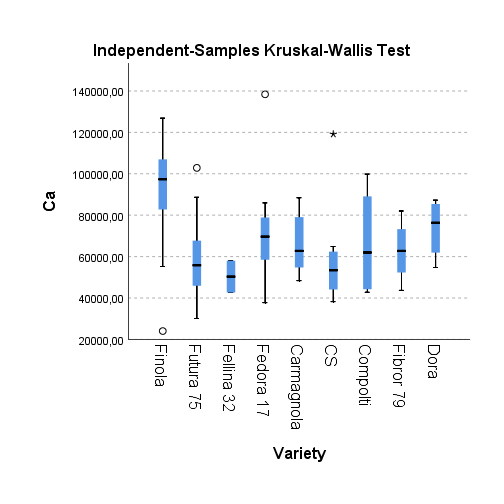


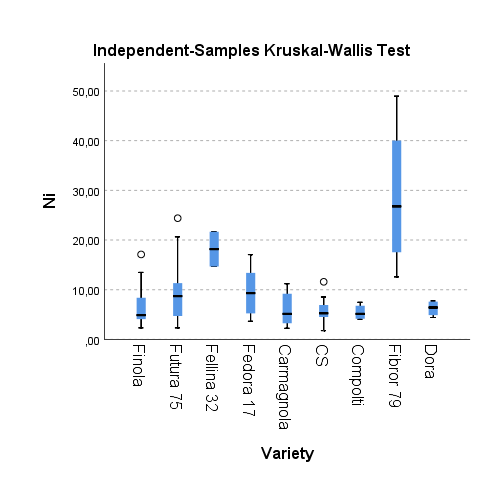

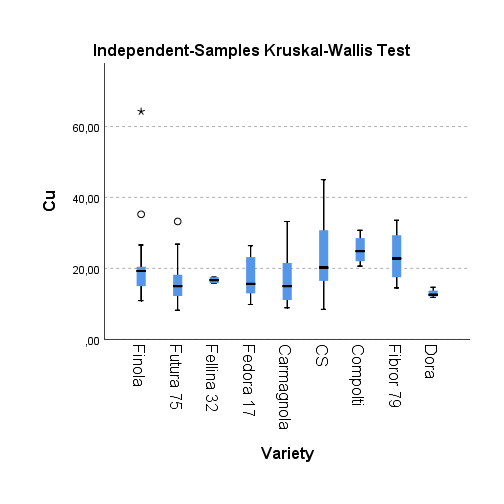

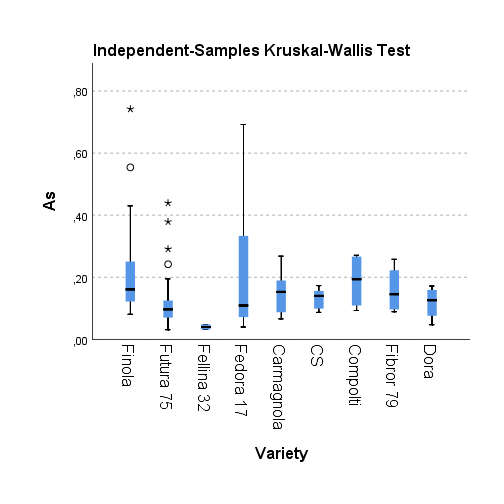


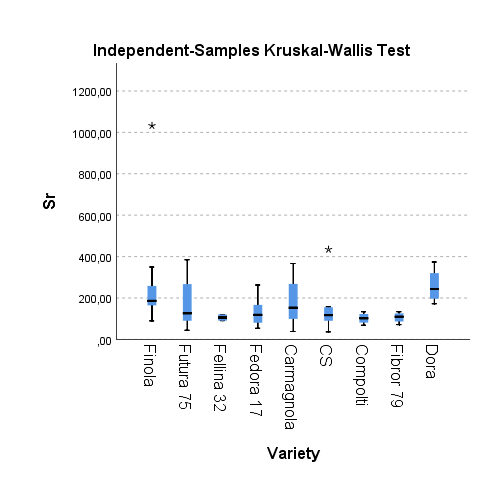

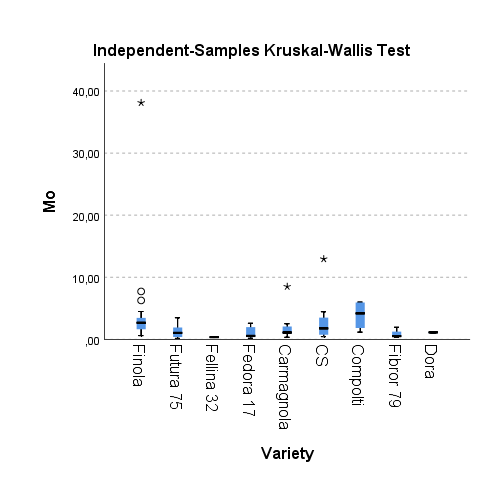

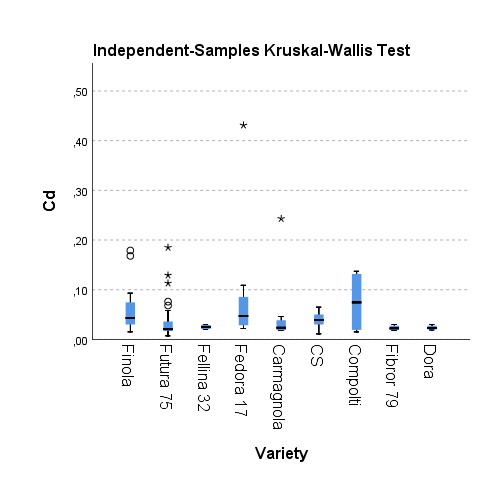


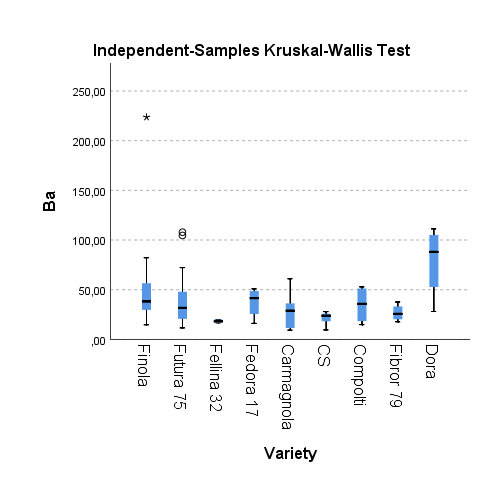

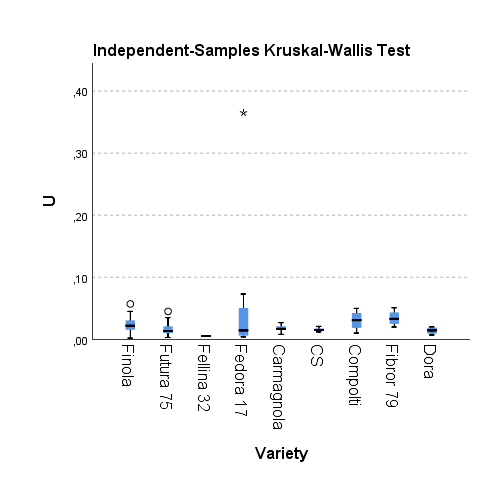


**Table S4:** Concentrations of elements in leaves/flowers and seeds of cannabis samples.

| **LEAVES** | **Na** | **Mg** | **P** | **K** | **Ca** | **B** | **Al** | **Ti** | **V** | **Cr** | **Mn** | **Fe** | **Co** | **Ni** | **Cu** |
| --- | --- | --- | --- | --- | --- | --- | --- | --- | --- | --- | --- | --- | --- | --- | --- |
| **1** | 112 | 13510 | 5366 | 23944 | 97351 | 221.568 | 469.658 | 16.058 | 0.840 | 2.275 | 80.418 | 423.758 | 0.343 | 13.276 | 14.843 |
| **4** | 391 | 17434 | 9174 | 28068 | 105853 | 178.164 | 326.815 | 8.725 | 0.543 | 0.726 | 320.130 | 399.733 | 0.473 | 7.076 | 24.492 |
| **12** | 184 | 9720 | 6574 | 38183 | 88333 | 175.906 | 301.555 | 11.545 | 0.521 | 0.982 | 194.267 | 404.709 | 0.201 | 5.279 | 15.069 |
| **18** | 116 | 11408 | 2018 | 19623 | 52637 | 142.275 | 291.751 | 8.380 | 0.487 | 0.603 | 391.206 | 270.394 | 0.120 | 7.024 | 11.477 |
| **31** | 35 | 10288 | 4395 | 25269 | 39481 | 75.819 | 99.435 | 6.255 | 0.208 | 0.677 | 113.732 | 181.160 | 0.146 | 11.332 | 12.223 |
| **35** | 48 | 9975 | 4027 | 30803 | 64537 | 152.716 | 167.539 | 8.914 | 0.364 | 0.874 | 220.833 | 302.316 | 0.116 | 2.590 | 16.908 |
| **36** | 70 | 9880 | 3207 | 19205 | 54359 | 122.780 | 129.660 | 7.046 | 0.227 | 0.542 | 182.635 | 303.549 | 0.134 | 3.651 | 15.591 |
| **38** | 59 | 10013 | 4488 | 18572 | 45917 | 119.329 | 176.773 | 8.971 | 0.375 | 0.802 | 125.679 | 417.916 | 0.145 | 2.302 | 14.250 |
| **39** | 28 | 12301 | 3051 | 22534 | 74728 | 153.442 | 131.811 | 7.878 | 0.264 | 1.126 | 190.786 | 216.147 | 0.176 | 9.066 | 10.737 |
| **40** | 46 | 11951 | 4506 | 26149 | 83514 | 159.954 | 115.207 | 5.526 | 0.232 | 0.578 | 165.204 | 219.996 | 0.158 | 4.415 | 12.784 |
| **41** | 82 | 12420 | 3719 | 17509 | 40681 | 197.580 | 135.256 | 7.473 | 0.343 | 0.572 | 162.281 | 194.249 | 0.158 | 3.571 | 12.357 |
| **42** | 36 | 10012 | 2816 | 23568 | 59846 | 174.656 | 241.939 | 12.794 | 0.507 | 1.026 | 163.521 | 284.048 | 0.150 | 2.538 | 12.490 |
| **45** | 70 | 10124 | 2590 | 21318 | 67719 | 199.949 | 357.034 | 18.628 | 0.819 | 1.455 | 144.716 | 384.133 | 0.218 | 2.936 | 11.996 |
| **46** | 60 | 12515 | 3267 | 19053 | 69168 | 184.626 | 254.665 | 14.393 | 0.643 | 1.047 | 151.746 | 258.848 | 0.212 | 5.370 | 11.834 |
| **47** | 46 | 13623 | 4172 | 23500 | 54130 | 191.240 | 180.031 | 10.106 | 0.388 | 0.926 | 177.867 | 272.284 | 0.171 | 4.879 | 17.163 |
| **48** | 62 | 14302 | 4855 | 21183 | 73099 | 227.450 | 272.936 | 15.228 | 0.615 | 1.305 | 179.387 | 316.622 | 0.257 | 6.165 | 15.287 |
| **49** | 35 | 11230 | 2562 | 22675 | 57163 | 209.747 | 416.504 | 21.325 | 0.869 | 1.637 | 170.427 | 402.691 | 0.213 | 4.034 | 18.205 |
| **56** | 660 | 10858 | 3913 | 28380 | 87209 | 145.173 | 270.549 | 6.633 | 0.535 | 1.133 | 229.893 | 395.354 | 0.340 | 4.884 | 64.222 |
| **57** | 58 | 10318 | 2255 | 24050 | 61850 | 186.486 | 79.610 | 4.367 | 0.160 | 0.494 | 116.408 | 162.430 | 0.160 | 6.098 | 15.476 |
| **58** | 93 | 11682 | 4708 | 19096 | 69577 | 220.292 | 138.069 | 6.418 | 0.295 | 0.806 | 178.744 | 217.561 | 0.387 | 14.288 | 14.455 |
| **61** | 106 | 14682 | 7435 | 34921 | 123176 | 223.907 | 524.977 | 17.846 | 1.043 | 1.732 | 444.601 | 543.992 | 0.379 | 4.054 | 19.266 |
| **SEEDS** | **Na** | **Mg** | **P** | **K** | **Ca** | **B** | **Al** | **Ti** | **V** | **Cr** | **Mn** | **Fe** | **Co** | **Ni** | **Cu** |
| **1** | 29 | 6538 | 8909 | 12776 | 7298 | 26.842 | 37.250 | 1.471 | 0.047 | 0.184 | 64.374 | 133.520 | 0.115 | 4.976 | 20.973 |
| **4** | 57 | 9671 | 14785 | 12547 | 5103 | 24.425 | 23.814 | 1.096 | 0.025 | 0.099 | 153.863 | 244.003 | 0.640 | 4.040 | 29.887 |
| **12** | 41 | 5004 | 8187 | 11233 | 2140 | 13.694 | 9.927 | 0.586 | 0.021 | 0.065 | 106.909 | 130.033 | 0.123 | 5.125 | 12.038 |
| **18** | 33 | 8515 | 9343 | 13725 | 5371 | 27.468 | 21.583 | 0.857 | 0.051 | 0.125 | 287.212 | 265.739 | 0.090 | 5.764 | 22.912 |
| **31** | 155 | 5683 | 6853 | 11944 | 4165 | 14.535 | 6.897 | 0.308 | 0.023 | 0.093 | 92.338 | 104.186 | 0.109 | 9.321 | 14.826 |
| **35** | 22 | 6297 | 7670 | 13866 | 5186 | 16.035 | 12.776 | 0.627 | 0.038 | 0.104 | 168.739 | 171.897 | 0.107 | 2.349 | 21.292 |
| **36** | 38 | 9304 | 11450 | 15534 | 10092 | 31.530 | 26.510 | 1.391 | 0.064 | 0.195 | 204.907 | 234.487 | 0.248 | 6.813 | 31.324 |
| **38** | 22 | 6655 | 9294 | 13496 | 5243 | 20.980 | 21.980 | 1.079 | 0.058 | 0.094 | 113.992 | 190.573 | 0.100 | 2.118 | 19.023 |
| **39** | 28 | 8758 | 10820 | 13130 | 5868 | 22.728 | 23.833 | 0.869 | 0.033 | 0.182 | 146.441 | 189.981 | 0.215 | 5.252 | 22.079 |
| **40** | 27 | 9388 | 12388 | 15748 | 6920 | 22.883 | 15.465 | 1.263 | 0.048 | 0.403 | 150.320 | 174.688 | 0.312 | 6.689 | 26.969 |
| **41** | 18 | 6410 | 6733 | 11764 | 5883 | 26.698 | 17.503 | 0.953 | 0.036 | 0.107 | 139.083 | 129.383 | 0.175 | 3.946 | 16.060 |
| **42** | 17 | 6529 | 7421 | 11906 | 4266 | 19.513 | 18.800 | 1.074 | 0.051 | 0.274 | 150.905 | 153.823 | 0.088 | 2.904 | 18.953 |
| **45** | 23 | 6519 | 7717 | 11592 | 6972 | 21.238 | 24.961 | 1.284 | 0.053 | 0.241 | 118.269 | 155.739 | 0.141 | 3.122 | 16.688 |
| **46** | 21 | 7489 | 8779 | 13336 | 6628 | 22.484 | 18.922 | 0.808 | 0.040 | 0.151 | 146.166 | 113.004 | 0.292 | 4.706 | 22.705 |
| **47** | 18 | 7264 | 8504 | 11926 | 5350 | 21.333 | 17.306 | 0.741 | 0.043 | 0.156 | 165.712 | 166.524 | 0.124 | 4.875 | 23.159 |
| **48** | 22 | 7328 | 7547 | 13892 | 10792 | 29.160 | 33.940 | 1.808 | 0.051 | 0.169 | 153.779 | 125.049 | 0.169 | 4.164 | 20.201 |
| **49** | 18 | 7937 | 8667 | 13313 | 7469 | 25.616 | 19.369 | 0.981 | 0.061 | 0.128 | 148.053 | 166.742 | 0.096 | 4.132 | 31.899 |
| **56** | 141 | 8790 | 10973 | 13282 | 7980 | 25.731 | 27.002 | 1.224 | 0.052 | 0.226 | 178.127 | 232.561 | 0.151 | 8.194 | 32.229 |
| **57** | 24 | 7828. | 8558 | 13135 | 3794 | 20.870 | 12.721 | 0.520 | 0.017 | 0.124 | 177.439 | 167.748 | 0.262 | 8.209 | 23.566 |
| **58** | 97 | 5944 | 7937 | 11535 | 4063 | 20.358 | 22.283 | 0.662 | 0.037 | 0.176 | 127.328 | 137.565 | 0.261 | 9.641 | 18.501 |
| **61** | 39 | 8641 | 12940 | 12080 | 4030 | 23.316 | 25.497 | 0.926 | 0.053 | 0.217 | 175.661 | 167.352 | 0.243 | 10.081 | 25.946 |

| **LEAVES** | **Zn** | **As** | **Se** | **Sr** | **Mo** | **Ag** | **Cd** | **Sn** | **Sb** | **Ba** | **Hg** | **Tl** | **Pb** | **U** |
| --- | --- | --- | --- | --- | --- | --- | --- | --- | --- | --- | --- | --- | --- | --- |
| **1** | 42.880 | 0.175 | 0.082 | 159.382 | 2.471 | 0.142 | 0.054 | 0.082 | 0.019 | 52.902 | 0.013 | 0.016 | 0.401 | 0.021 |
| **4** | 98.819 | 0.161 | 0.099 | 348.403 | 3.468 | 0.051 | 0.036 | 0.059 | 0.009 | 30.281 | 0.015 | 0.069 | 0.437 | 0.034 |
| **12** | 50.246 | 0.124 | 0.052 | 119.363 | 6.268 | 0.131 | 0.079 | 0.045 | 0.008 | 60.314 | 0.016 | 0.014 | 0.316 | 0.015 |
| **18** | 51.024 | 0.291 | 0.489 | 91.672 | 0.231 | 0.053 | 0.076 | 0.047 | 0.026 | 107.837 | 0.014 | 0.911 | 0.387 | 0.010 |
| **31** | 39.419 | 0.031 | 0.044 | 71.511 | 0.529 | 0.041 | 0.019 | 0.032 | 0.007 | 11.662 | 0.006 | 0.145 | 0.095 | 0.004 |
| **35** | 53.866 | 0.059 | 0.088 | 123.647 | 1.789 | 0.048 | 0.012 | 0.037 | 0.011 | 25.757 | 0.013 | 0.007 | 0.235 | 0.008 |
| **36** | 44.681 | 0.040 | 0.054 | 111.970 | 0.582 | 0.033 | 0.022 | 0.025 | 0.009 | 22.061 | 0.008 | 0.057 | 0.183 | 0.004 |
| **38** | 44.985 | 0.067 | 0.078 | 171.238 | 1.912 | 0.025 | 0.015 | 0.094 | 0.019 | 16.787 | 0.009 | 0.073 | 0.231 | 0.007 |
| **39** | 31.645 | 0.057 | 0.070 | 129.331 | 0.314 | 0.043 | 0.014 | 0.034 | 0.015 | 19.212 | 0.011 | 0.160 | 0.164 | 0.007 |
| **40** | 31.480 | 0.047 | 0.117 | 220.651 | 1.064 | 0.179 | 0.025 | 0.032 | 0.009 | 28.082 | 0.021 | 0.033 | 0.550 | 0.007 |
| **41** | 23.616 | 0.075 | 0.059 | 310.018 | 2.467 | 0.122 | 0.019 | 0.029 | 0.018 | 40.105 | 0.013 | 0.040 | 0.207 | 0.012 |
| **42** | 25.297 | 0.077 | 0.058 | 270.660 | 1.328 | 0.042 | 0.021 | 0.031 | 0.017 | 52.955 | 0.011 | 0.028 | 0.244 | 0.008 |
| **45** | 54.954 | 0.117 | 0.058 | 114.479 | 1.001 | 0.113 | 0.014 | 0.064 | 0.015 | 63.233 | 0.016 | 0.083 | 0.346 | 0.014 |
| **46** | 63.713 | 0.105 | 0.055 | 373.881 | 1.180 | 0.154 | 0.021 | 0.038 | 0.010 | 98.889 | 0.010 | 0.072 | 0.293 | 0.011 |
| **47** | 23.131 | 0.079 | 1.244 | 267.156 | 2.626 | 0.066 | 0.020 | 0.038 | 0.015 | 37.097 | 0.013 | 0.039 | 0.285 | 0.018 |
| **48** | 66.660 | 0.101 | 0.043 | 333.410 | 1.083 | 0.050 | 0.019 | 0.065 | 0.028 | 104.594 | 0.019 | 0.036 | 0.392 | 0.014 |
| **49** | 54.335 | 0.095 | 0.028 | 193.635 | 0.373 | 0.042 | 0.017 | 0.038 | 0.012 | 41.397 | 0.010 | 0.031 | 0.271 | 0.014 |
| **56** | 106.625 | 0.098 | 0.117 | 192.773 | 0.595 | 0.024 | 0.029 | 0.037 | 0.063 | 31.097 | 0.013 | 0.043 | 0.405 | 0.032 |
| **57** | 36.578 | 0.070 | 0.044 | 44.306 | 0.162 | 0.089 | 0.012 | 0.021 | 0.008 | 37.151 | 0.011 | 0.046 | 0.145 | 0.003 |
| **58** | 37.851 | 0.091 | 0.052 | 53.759 | 0.183 | 0.048 | 0.057 | 0.028 | 0.011 | 48.542 | 0.012 | 0.077 | 0.219 | 0.005 |
| **61** | 73.821 | 0.285 | 0.042 | 266.236 | 2.706 | 0.097 | 0.077 | 0.078 | 0.033 | 25.861 | 0.016 | 0.096 | 1.143 | 0.037 |
| **SEEDS** | **Zn** | **As** | **Se** | **Sr** | **Mo** | **Ag** | **Cd** | **Sn** | **Sb** | **Ba** | **Hg** | **Tl** | **Pb** | **U** |
| **1** | 53.598 | 0.027 | 0.030 | 13.392 | 1.237 | 0.043 | 0.024 | 0.027 | <LOQ | 7.124 | 0.002 | 0.002 | 0.041 | 0.002 |
| **4** | 98.701 | 0.031 | 0.033 | 21.541 | 1.901 | 0.079 | 0.039 | 0.024 | <LOQ | 5.154 | 0.003 | 0.020 | 0.047 | 0.001 |
| **12** | 53.053 | 0.010 | 0.008 | 3.774 | 1.474 | 0.384 | 0.047 | 0.008 | <LOQ | 4.286 | 0.003 | 0.001 | 0.027 | <LOQ |
| **18** | 103.819 | 0.016 | 0.087 | 22.687 | 0.482 | 0.040 | 0.050 | 0.031 | 0.026 | 20.042 | 0.002 | 0.384 | 0.462 | 0.001 |
| **31** | 48.814 | 0.007 | 0.015 | 9.878 | 0.310 | 0.159 | 0.027 | 0.011 | 0.000 | 4.068 | 0.003 | 0.053 | 0.020 | <LOQ |
| **35** | 68.269 | 0.011 | 0.037 | 12.674 | 0.728 | 0.141 | 0.008 | 0.012 | 0.000 | 5.403 | 0.004 | 0.001 | 0.019 | 0.000 |
| **36** | 97.621 | 0.010 | 0.035 | 21.999 | 0.903 | 0.242 | 0.028 | 0.019 | 0.002 | 7.999 | 0.005 | 0.023 | 0.039 | 0.000 |
| **38** | 68.557 | 0.013 | 0.080 | 23.724 | 0.855 | 0.141 | 0.011 | 0.015 | 0.001 | 4.810 | 0.002 | 0.027 | 0.023 | 0.000 |
| **39** | 74.749 | 0.014 | 0.061 | 21.926 | 0.897 | 0.193 | 0.022 | 0.023 | 0.000 | 6.648 | 0.004 | 0.009 | 0.031 | 0.000 |
| **40** | 75.224 | 0.008 | 0.058 | 22.756 | 0.616 | 0.190 | 0.038 | 0.020 | <LOQ | 7.004 | 0.006 | 0.008 | 0.029 | 0.001 |
| **41** | 51.442 | 0.016 | 0.024 | 48.466 | 0.852 | 0.144 | 0.025 | 0.014 | 0.000 | 8.769 | 0.002 | 0.014 | 0.047 | 0.002 |
| **42** | 61.207 | 0.011 | 0.032 | 28.601 | 1.019 | 0.019 | 0.012 | 0.014 | 0.002 | 7.242 | 0.002 | 0.006 | 0.027 | 0.000 |
| **45** | 71.362 | 0.016 | 0.027 | 16.353 | 0.533 | 0.046 | 0.011 | 0.019 | 0.000 | 12.912 | 0.003 | 0.024 | 0.041 | 0.004 |
| **46** | 71.398 | 0.017 | 0.032 | 37.417 | 0.866 | 0.018 | 0.011 | 0.015 | 0.002 | 14.956 | 0.001 | 0.017 | 0.025 | 0.000 |
| **47** | 50.084 | 0.011 | 0.889 | 32.181 | 1.033 | 0.019 | 0.019 | 0.014 | <LOQ | 6.684 | 0.001 | 0.009 | 0.032 | 0.002 |
| **48** | 67.918 | 0.017 | 0.007 | 52.151 | 0.520 | 0.059 | 0.016 | 0.014 | 0.002 | 24.753 | 0.002 | 0.006 | 0.041 | 0.001 |
| **49** | 89.557 | 0.008 | 0.038 | 33.150 | 0.563 | 0.018 | 0.007 | 0.011 | <LOQ | 8.698 | 0.003 | 0.009 | 0.026 | 0.000 |
| **56** | 112.283 | 0.014 | 0.106 | 21.032 | 0.609 | 0.155 | 0.113 | 0.032 | 0.002 | 7.344 | 0.004 | 0.009 | 0.139 | 0.003 |
| **57** | 79.857 | 0.014 | 0.034 | 3.287 | 0.170 | 0.082 | 0.064 | 0.094 | <LOQ | 6.113 | 0.002 | 0.008 | 0.059 | <LOQ |
| **58** | 59.160 | 0.012 | 0.042 | 3.937 | 0.156 | 0.078 | 0.035 | 0.029 | 0.001 | 7.376 | 0.006 | 0.014 | 0.373 | 0.000 |
| **61** | 86.532 | 0.024 | 0.000 | 11.314 | 1.136 | 0.120 | 0.074 | 0.056 | 0.007 | 4.728 | 0.003 | 0.016 | 0.138 | 0.001 |

**Figure S3:** Accumulation of elements in leaves and seeds. <LOQ values are set at 0 ppm.

**
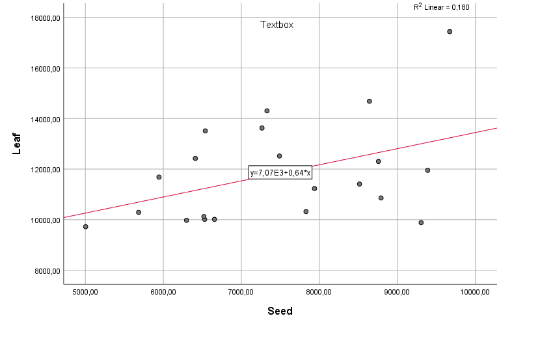

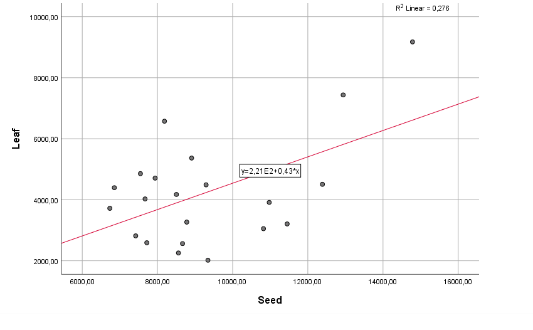

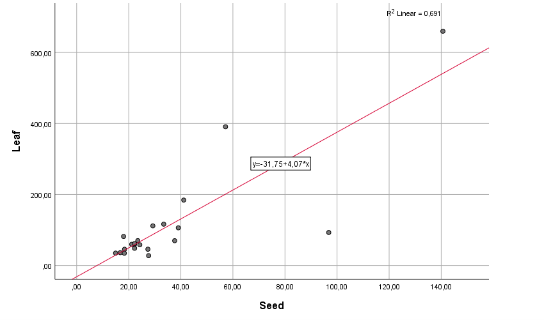
Na Mg P**

leaf:seed : y=31.75 + 4.07*x; p=0.000; r2=0.691

leaf:seed : y=2.21E2 + 0.43*x; p>0.05; r2=0.278

leaf:seed : y=7.07E3 + 0.64*x; p>0.05; r2=0.180

**
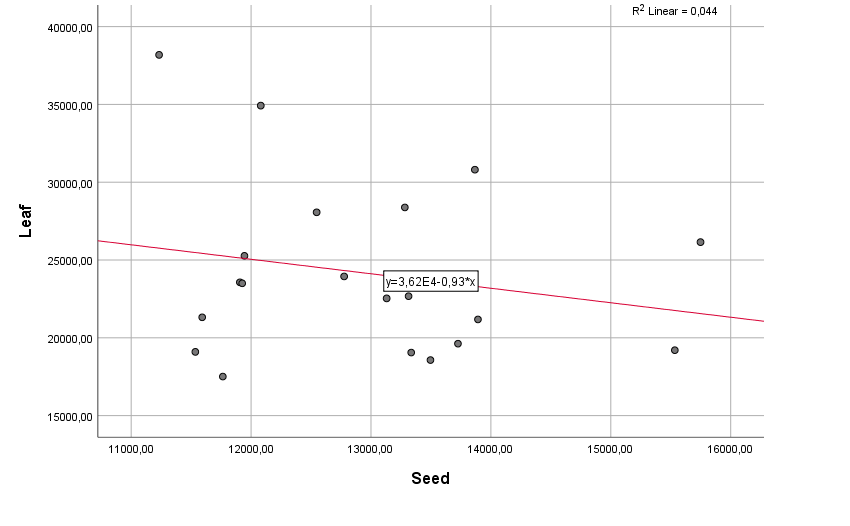
K Ca B**

**
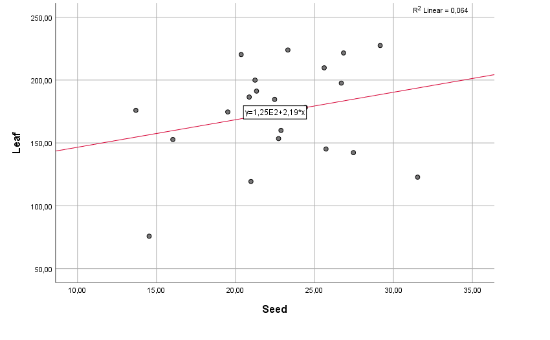

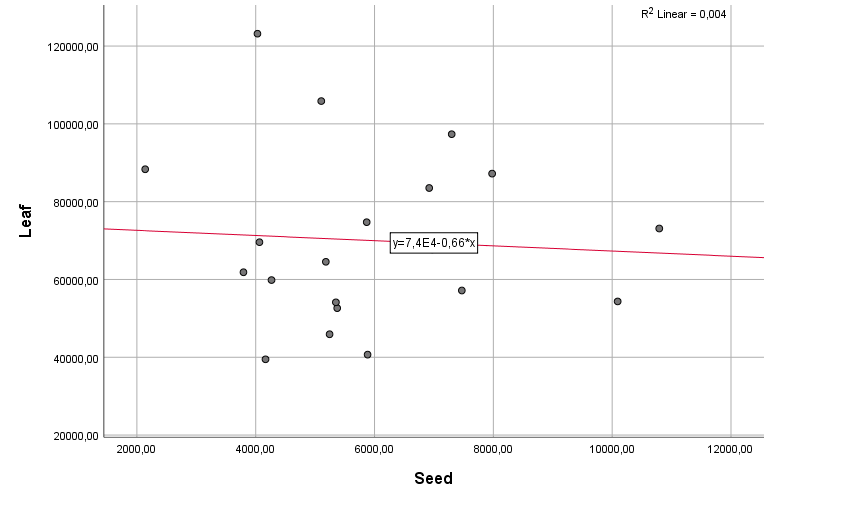
**

leaf:seed : y=3.62E4 – 0.93*x; p>0.05; r2=0.044

leaf:seed : y=1.25E2 + 2.19*x; p>0.05; r2=0.054

leaf:seed : y=7.4E4 + 0.66*x; p>0.05; r2=0.004

**
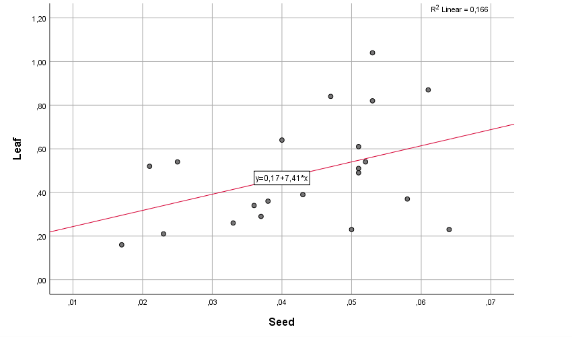

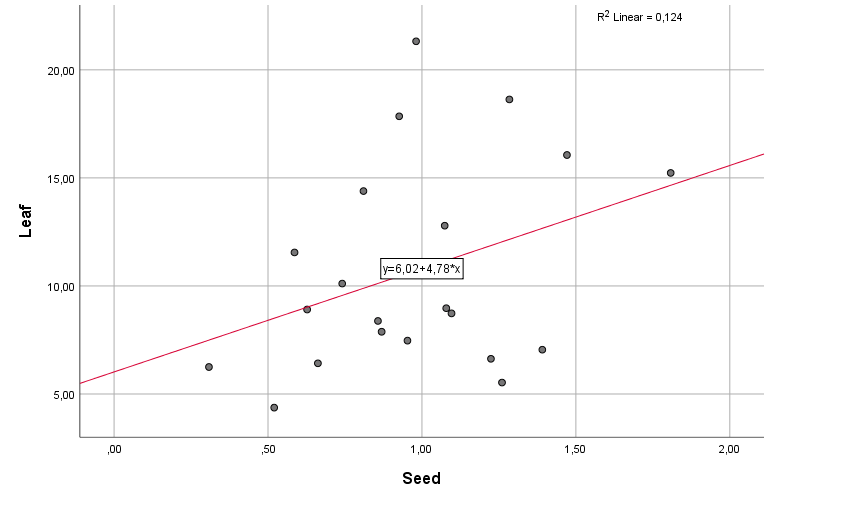

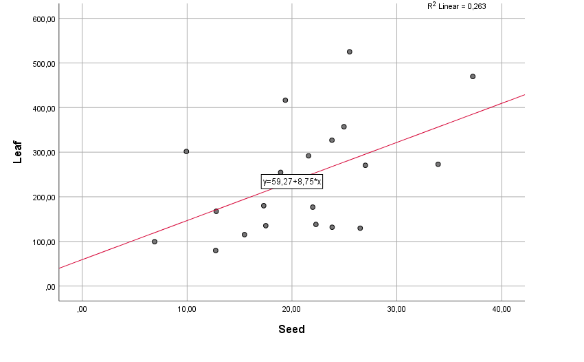
Al Ti V**

leaf:seed : y=0.17 + 7.41*x; p>0.05; r2=0.166

leaf:seed : y=6.02 + 4.78*x; p>0.05; r2=0.124

leaf:seed : y=59.27 + 8.75*x; p=0.017; r2=0.263

**
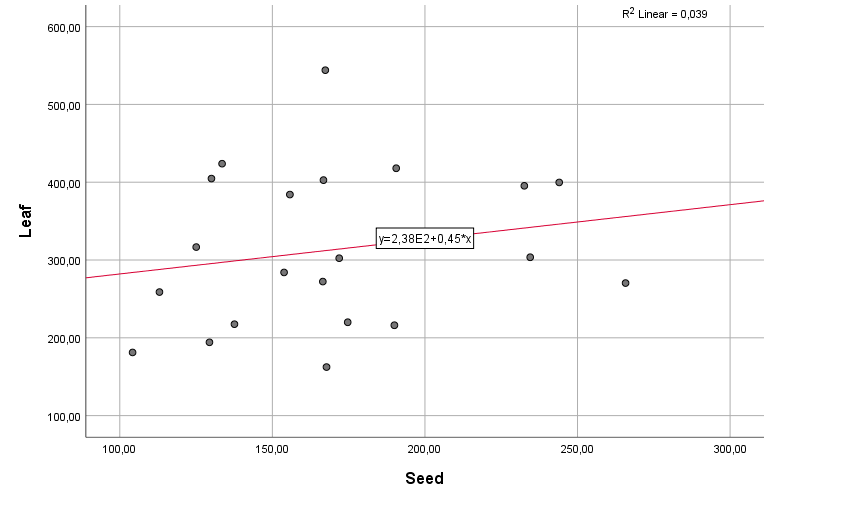

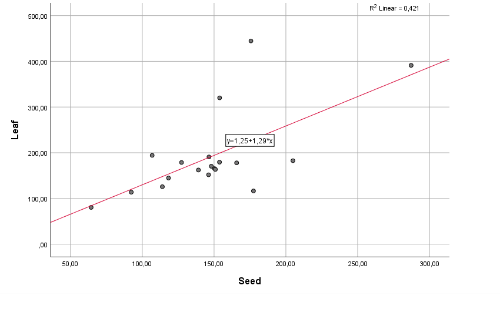

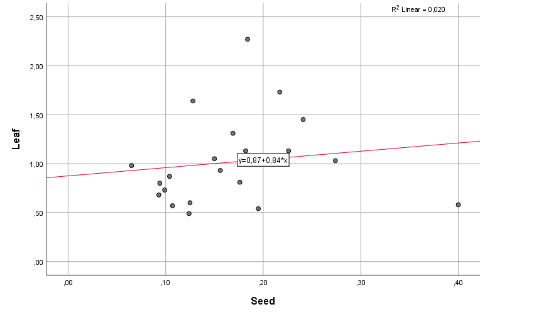
Cr Mn Fe**

leaf:seed : y=1.25 + 1.29*x; p=0.001; r2=0.421

leaf:seed : y=2.38E2 + 0.45*x; p>0.05; r2=0.039

leaf:seed : y=0.87 + 0.84*x; p>0.05; r2=0.020

**
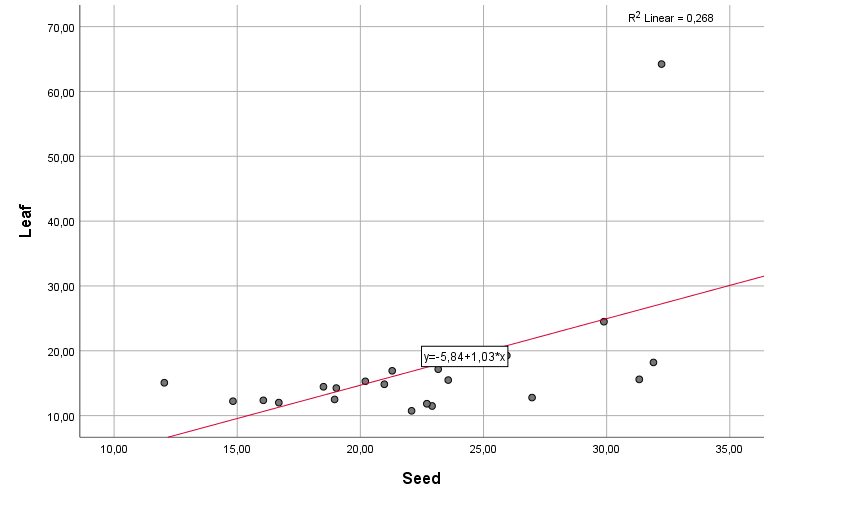

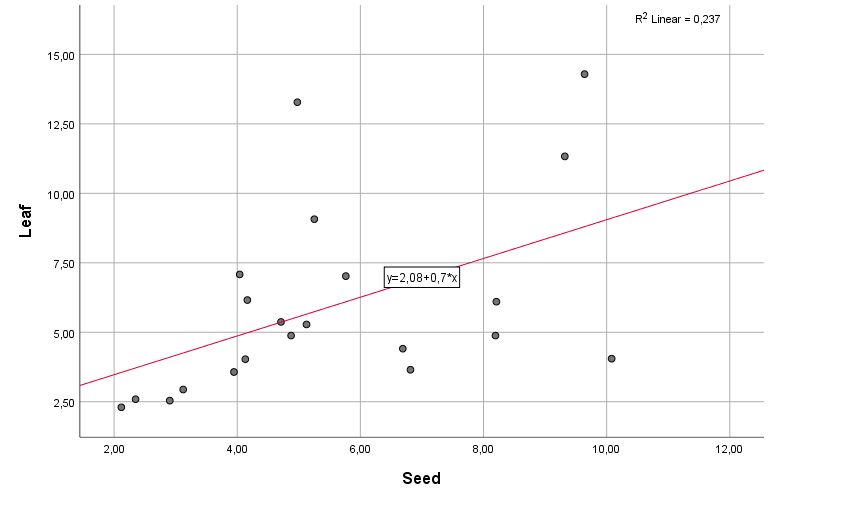

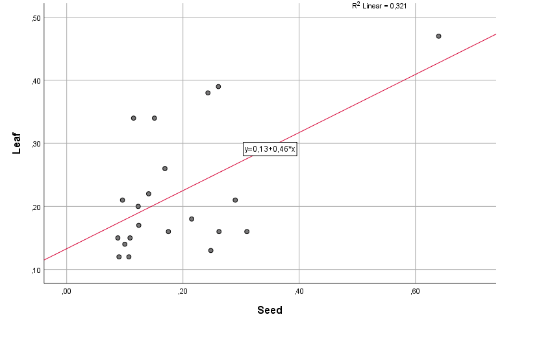
Co Ni Cu**

leaf:seed : y=-5.84 + 1.03*x; p=0.016; r2=0.268

leaf:seed : y=0.13 + 0.46*x; p=0.007; r2=0.321

leaf:seed : y=2.08 + 0.7*x; p=0.025; r2=0.237

**
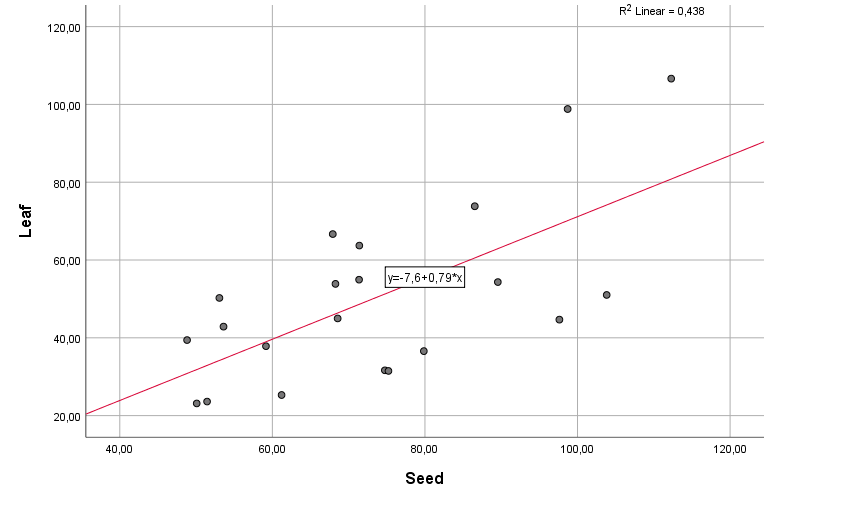
Zn As Se**

**
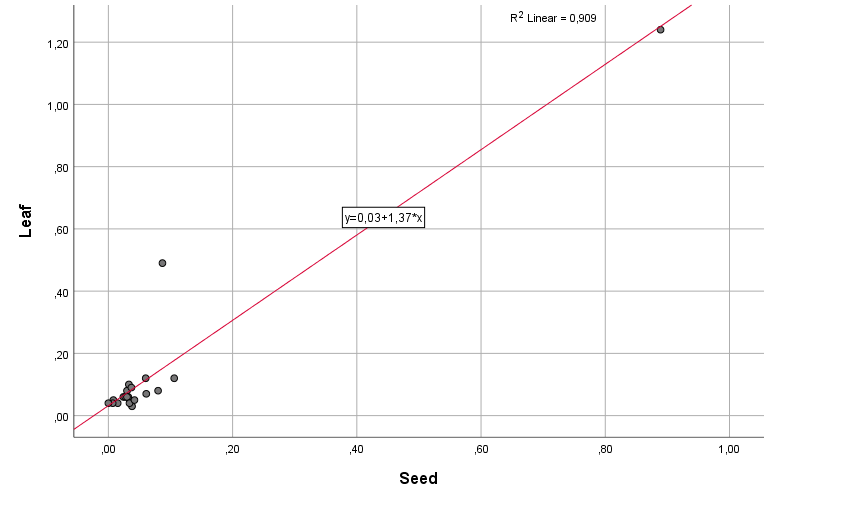

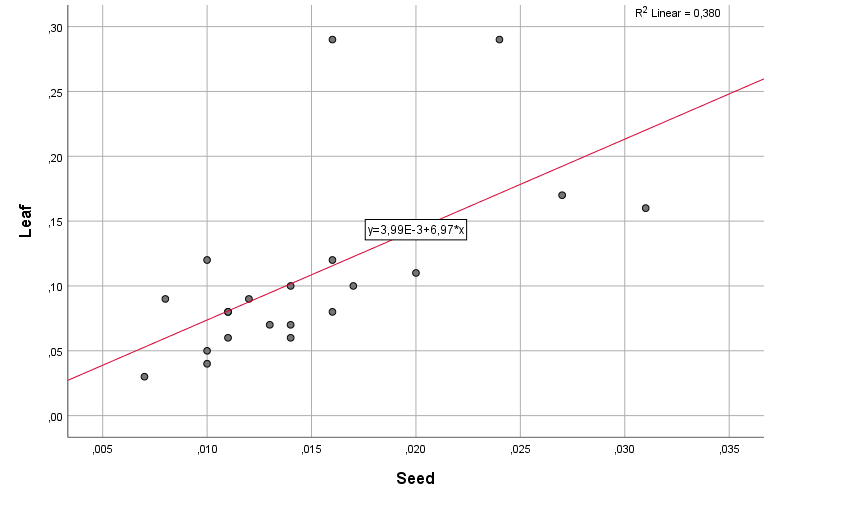
**

leaf:seed : y=3.99E-3 + 6.97*x; p=0.003; r2=0.380

leaf:seed : y=0.03 + 1.37*x; p=0.000; r2=0.909

leaf:seed : y=-7.6 + 0.79*x; p=0.001; r2=0.438

**
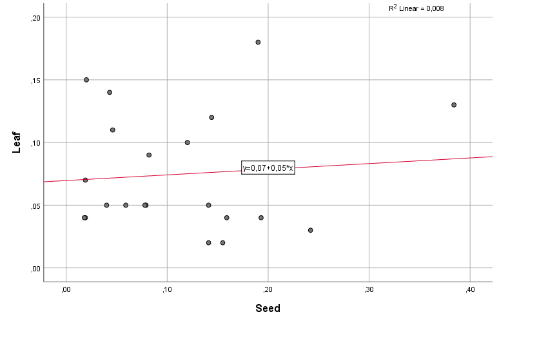

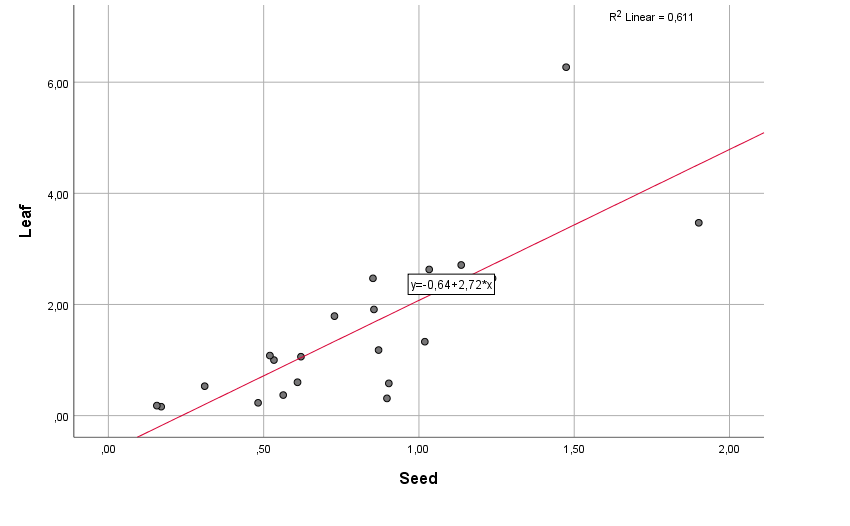

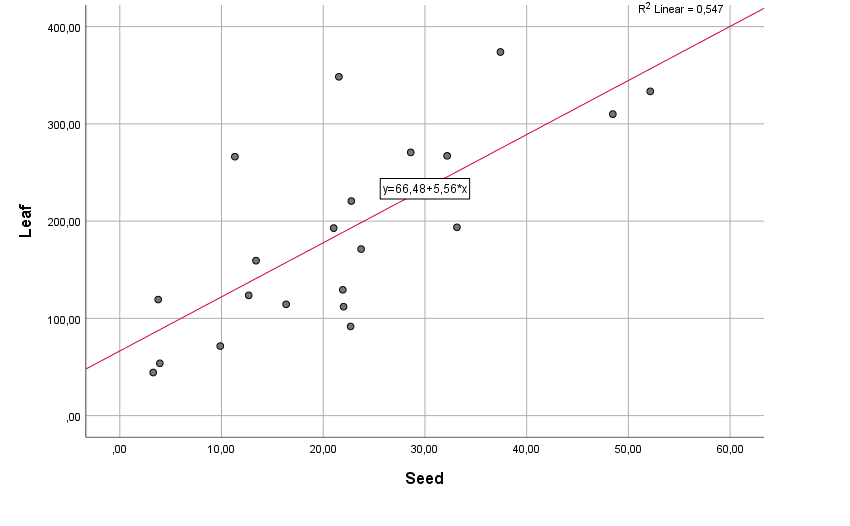
Sr Mo Ag**

leaf:seed : y=0.07 + 0.05*x; p>0.05; r2=0.008

leaf:seed : y=0.64 + 2.72*x; p=0.000; r2=0.611

leaf:seed : y=66.48 + 5.56*x; p=0.000; r2=0.547

**
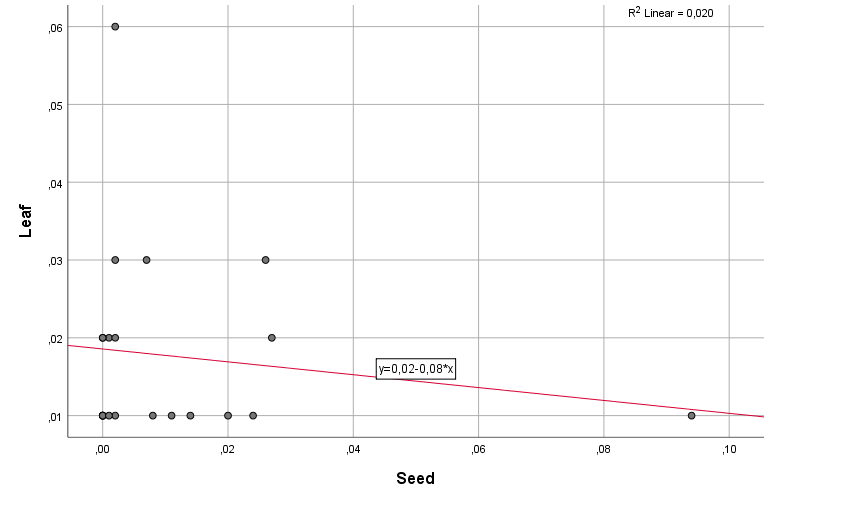

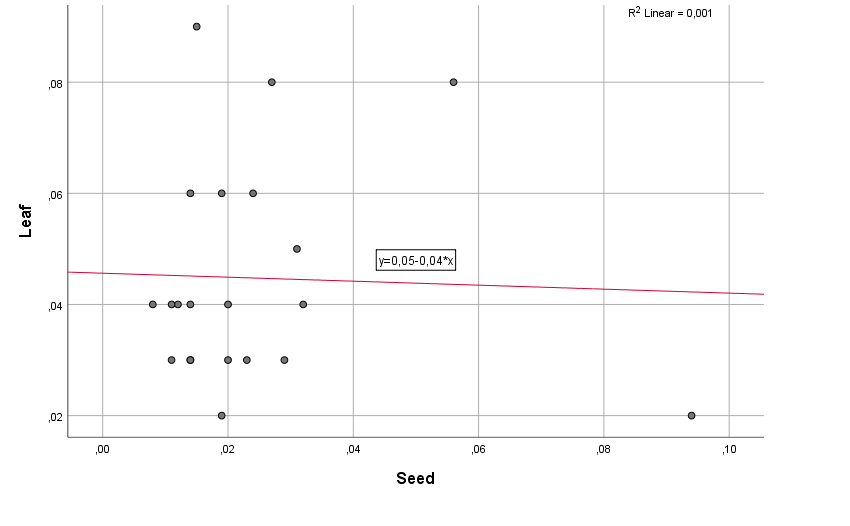

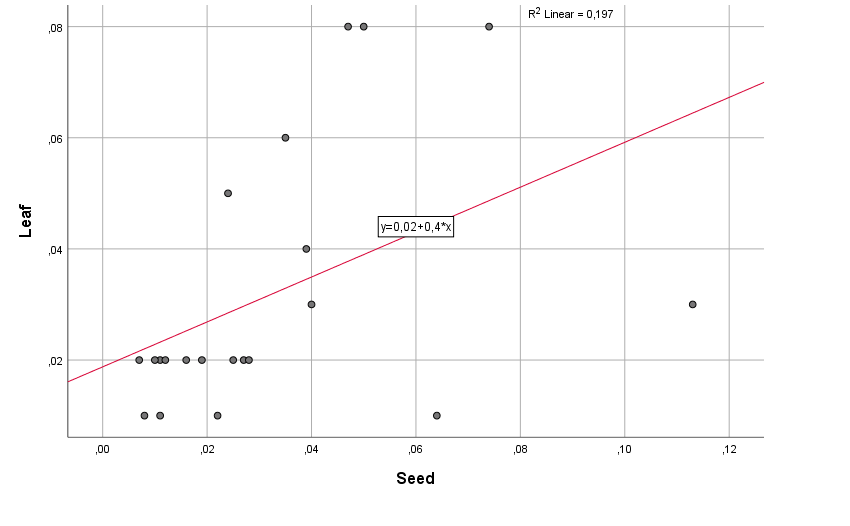
Cd Sn Sb**

leaf:seed : y=0.02 - 0.08*x; p>0.05; r2=0.020

leaf:seed : y=0.05 - 0.04*x; p>0.05; r2=0.001

leaf:seed : y=0.02 + 0.4*x; p=0.044; r2=0.197

**
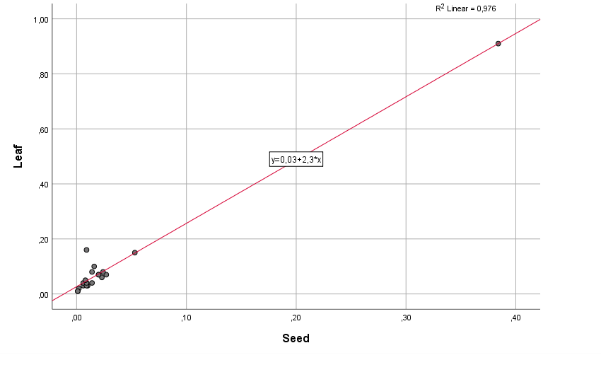

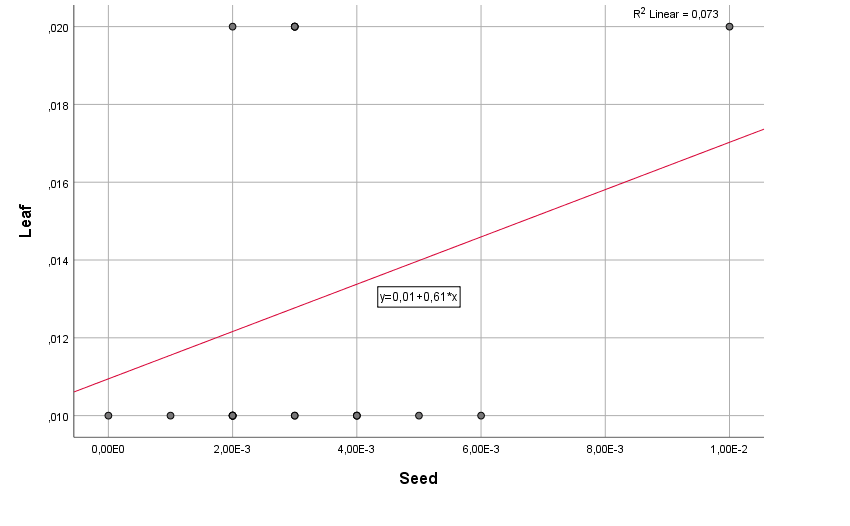

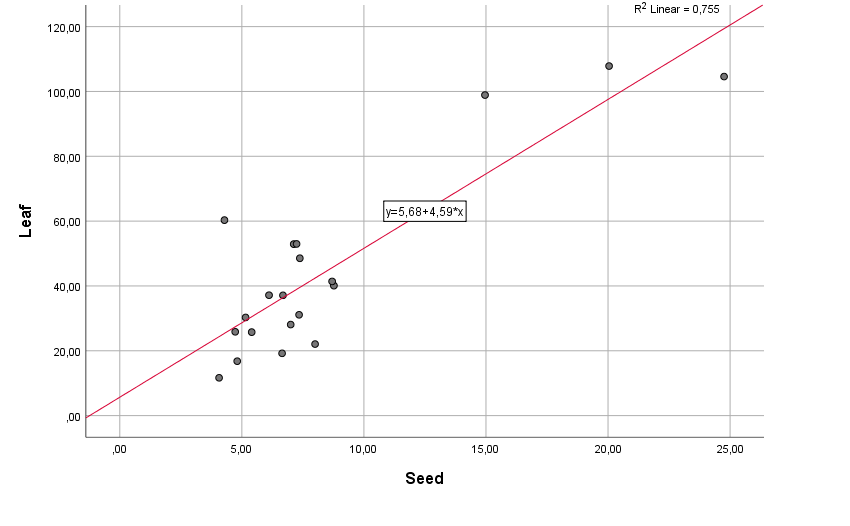
Ba Hg Tl**

leaf:seed : y=0.03 + 2.3*x; p=0.000; r2=0.976

leaf:seed : y=5.68 + 4.59*x; p=0.000; r2=0.755

leaf:seed : y=0.01 + 0.61*x; p>0.05; r2=0.073

**
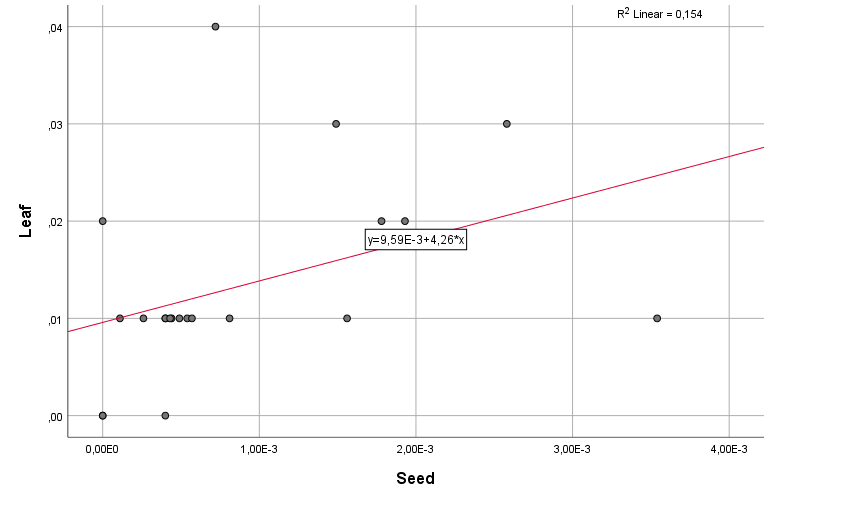

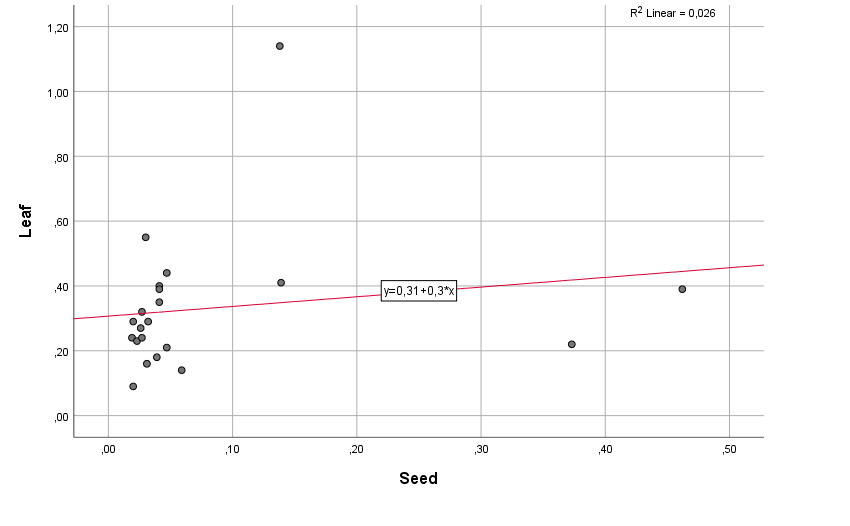
Pb U**

leaf:seed : y=9.59E-3 + 4.26*x; p=0.000; r2=0.154

leaf:seed : y=0.31 + 0.3*x; p>0.05; r2=0.026

**Table S5:** Calculated EDI and HQ for the toxic elements.

|  |  | **As** |  |  | **Cd** |  |  | **Hg** |  |  | **Pb** |  |  | **Cr** |  |  | **Ni** |  |
| --- | --- | --- | --- | --- | --- | --- | --- | --- | --- | --- | --- | --- | --- | --- | --- | --- | --- | --- |
| **No of samples** | **C (ppb)** | **EDI** | **HQ** | **C (ppb)** | **EDI** | **HQ** | **C (ppb)** | **EDI** | **HQ** | **C (ppb)** | **EDI** | **HQ** | **C (ppb)** | **EDI** | **HQ** | **C (ppb)** | **EDI** | **HQ** |
| 1 | 174.673 | 7.5E-09 | 0.025 | 54.178 | 2.3E-09 | 0.002 | 12.772 | 5.5E-10 | 0.005 | 401.230 | 1.7E-08 | 0.009 | 2.275 | 9.7E-08 | 0.032 | 13.276 | 5.7E-07 | 0.028 |
| 2 | 690.000 | 3.0E-08 | 0.099 | 19.105 | 8.2E-10 | 0.001 | 20.058 | 8.6E-10 | 0.009 | 1290.430 | 5.5E-08 | 0.028 | 1.503 | 6.4E-08 | 0.021 | 2.447 | 1.0E-07 | 0.005 |
| 3 | 249.280 | 1.1E-08 | 0.036 | 42.764 | 1.8E-09 | 0.002 | 11.400 | 4.9E-10 | 0.005 | 651.950 | 2.8E-08 | 0.014 | 4.268 | 1.8E-07 | 0.061 | 12.356 | 5.3E-07 | 0.026 |
| 4 | 161.248 | 6.9E-09 | 0.023 | 35.770 | 1.5E-09 | 0.002 | 15.275 | 6.5E-10 | 0.007 | 436.831 | 1.9E-08 | 0.009 | 0.726 | 3.1E-08 | 0.010 | 7.076 | 3.0E-07 | 0.015 |
| 5 | 231.755 | 9.9E-09 | 0.033 | 28.071 | 1.2E-09 | 0.001 | 8.200 | 3.5E-10 | 0.004 | 596.814 | 2.6E-08 | 0.013 | 4.098 | 1.8E-07 | 0.059 | 9.657 | 4.1E-07 | 0.021 |
| 6 | 195.203 | 8.4E-09 | 0.028 | 19.003 | 8.1E-10 | 0.001 | 9.704 | 4.2E-10 | 0.004 | 627.109 | 2.7E-08 | 0.013 | 1.052 | 4.5E-08 | 0.015 | 3.604 | 1.5E-07 | 0.008 |
| 7 | 104.562 | 4.5E-09 | 0.015 | 23.327 | 1.0E-09 | 0.001 | 22.163 | 9.5E-10 | 0.009 | 190.488 | 8.2E-09 | 0.004 | 0.710 | 3.0E-08 | 0.010 | 4.269 | 1.8E-07 | 0.009 |
| 8 | 132.479 | 5.7E-09 | 0.019 | 93.199 | 4.0E-09 | 0.004 | 12.585 | 5.4E-10 | 0.005 | 305.020 | 1.3E-08 | 0.007 | 0.868 | 3.7E-08 | 0.012 | 5.79 | 2.5E-07 | 0.012 |
| 9 | 252.029 | 1.1E-08 | 0.036 | 72.476 | 3.1E-09 | 0.003 | 67.297 | 2.9E-09 | 0.029 | 658.468 | 2.8E-08 | 0.014 | 0.906 | 3.9E-08 | 0.013 | 17.095 | 7.3E-07 | 0.037 |
| 10 | 134.710 | 5.8E-09 | 0.019 | 25.264 | 1.1E-09 | 0.001 | 19.785 | 8.5E-10 | 0.008 | 453.245 | 1.9E-08 | 0.010 | 2.586 | 1.1E-07 | 0.037 | 13.475 | 5.8E-07 | 0.029 |
| 11 | 117.026 | 5.0E-09 | 0.017 | 57.563 | 2.5E-09 | 0.002 | 20.918 | 9.0E-10 | 0.009 | 324.373 | 1.4E-08 | 0.007 | 2.008 | 8.6E-08 | 0.029 | 4.665 | 2.0E-07 | 0.010 |
| 12 | 124.157 | 5.3E-09 | 0.018 | 79.083 | 3.4E-09 | 0.003 | 16.284 | 7.0E-10 | 0.007 | 316.291 | 1.4E-08 | 0.007 | 0.982 | 4.2E-08 | 0.014 | 5.279 | 2.3E-07 | 0.011 |
| 13 | 131.956 | 5.7E-09 | 0.019 | 14.786 | 6.3E-10 | 0.001 | 7.646 | 3.3E-10 | 0.003 | 153.725 | 6.6E-09 | 0.003 | 0.824 | 3.5E-08 | 0.012 | 4.106 | 1.8E-07 | 0.009 |
| 14 | 144.824 | 6.2E-09 | 0.021 | 64.680 | 2.8E-09 | 0.003 | 34.930 | 1.5E-09 | 0.015 | 397.568 | 1.7E-08 | 0.009 | 1.278 | 5.5E-08 | 0.018 | 11.591 | 5.0E-07 | 0.025 |
| 15 | 291.168 | 1.2E-08 | 0.042 | 75.769 | 3.2E-09 | 0.003 | 14.493 | 6.2E-10 | 0.006 | 387.165 | 1.7E-08 | 0.008 | 0.603 | 2.6E-08 | 0.009 | 7.024 | 3.0E-07 | 0.015 |
| 16 | 85.320 | 3.7E-09 | 0.012 | 17.652 | 7.6E-10 | 0.001 | 11.363 | 4.9E-10 | 0.005 | 240.319 | 1.0E-08 | 0.005 | 0.549 | 2.4E-08 | 0.008 | 2.231 | 9.6E-08 | 0.005 |
| 17 | 104.237 | 4.5E-09 | 0.015 | 18.749 | 8.0E-10 | 0.001 | 21.267 | 9.1E-10 | 0.009 | 323.467 | 1.4E-08 | 0.007 | 1.117 | 4.8E-08 | 0.016 | 6.691 | 2.9E-07 | 0.014 |
| 18 | 90.410 | 3.9E-09 | 0.013 | 25.618 | 1.1E-09 | 0.001 | 15.401 | 6.6E-10 | 0.007 | 243.760 | 1.0E-08 | 0.005 | 0.757 | 3.2E-08 | 0.011 | 4.858 | 2.1E-07 | 0.010 |
| 19 | 71.580 | 3.1E-09 | 0.010 | 30.836 | 1.3E-09 | 0.001 | 79.937 | 3.4E-09 | 0.034 | 735.255 | 3.2E-08 | 0.016 | 1.403 | 6.0E-08 | 0.020 | 10.622 | 4.6E-07 | 0.023 |
| 20 | 51.873 | 2.2E-09 | 0.007 | 33.798 | 1.4E-09 | 0.001 | 85.189 | 3.7E-09 | 0.037 | 242.629 | 1.0E-08 | 0.005 | 0.869 | 3.7E-08 | 0.012 | 12.472 | 5.3E-07 | 0.027 |
| 21 | 439.235 | 1.9E-08 | 0.063 | 113.127 | 4.8E-09 | 0.005 | 68.047 | 2.9E-09 | 0.029 | 636.934 | 2.7E-08 | 0.014 | 4.206 | 1.8E-07 | 0.060 | 11.939 | 5.1E-07 | 0.026 |
| 22 | 379.006 | 1.6E-08 | 0.054 | 47.547 | 2.0E-09 | 0.002 | 10.504 | 4.5E-10 | 0.005 | 1019.421 | 4.4E-08 | 0.022 | 5.078 | 2.2E-07 | 0.073 | 10.654 | 4.6E-07 | 0.023 |
| 23 | 124.978 | 5.4E-09 | 0.018 | 13.300 | 5.7E-10 | 0.001 | 10.690 | 4.6E-10 | 0.005 | 233.205 | 1.0E-08 | 0.005 | 1.990 | 8.5E-08 | 0.028 | 12.048 | 5.2E-07 | 0.026 |
| 24 | 120.437 | 5.2E-09 | 0.017 | 56.933 | 2.4E-09 | 0.002 | 18.193 | 7.8E-10 | 0.008 | 425.259 | 1.8E-08 | 0.009 | 1.403 | 6.0E-08 | 0.020 | 6.554 | 2.8E-07 | 0.014 |
| 25 | 692.339 | 3.0E-08 | 0.099 | 36.639 | 1.6E-09 | 0.002 | 14.219 | 6.1E-10 | 0.006 | 754.400 | 3.2E-08 | 0.016 | 3.682 | 1.6E-07 | 0.053 | 10.49 | 4.5E-07 | 0.022 |
| 26 | 58.510 | 2.5E-09 | 0.008 | 33.892 | 1.5E-09 | 0.001 | 7.129 | 3.1E-10 | 0.003 | 239.806 | 1.0E-08 | 0.005 | 0.896 | 3.8E-08 | 0.013 | 10.619 | 4.6E-07 | 0.023 |
| 27 | 112.852 | 4.8E-09 | 0.016 | 61.893 | 2.7E-09 | 0.003 | 9.637 | 4.1E-10 | 0.004 | 570.244 | 2.4E-08 | 0.012 | 2.127 | 9.1E-08 | 0.030 | 17.057 | 7.3E-07 | 0.037 |
| 28 | 111.799 | 4.8E-09 | 0.016 | 19.704 | 8.4E-10 | 0.001 | 15.099 | 6.5E-10 | 0.006 | 664.151 | 2.8E-08 | 0.014 | 1.478 | 6.3E-08 | 0.021 | 4.702 | 2.0E-07 | 0.010 |
| 29 | 32.247 | 1.4E-09 | 0.005 | 28.680 | 1.2E-09 | 0.001 | 10.257 | 4.4E-10 | 0.004 | 260.945 | 1.1E-08 | 0.006 | 0.757 | 3.2E-08 | 0.011 | 21.673 | 9.3E-07 | 0.046 |
| 30 | 47.642 | 2.0E-09 | 0.007 | 20.647 | 8.8E-10 | 0.001 | 12.836 | 5.5E-10 | 0.006 | 134.858 | 5.8E-09 | 0.003 | 0.628 | 2.7E-08 | 0.009 | 14.669 | 6.3E-07 | 0.031 |
| 31 | 31.139 | 1.3E-09 | 0.004 | 18.569 | 8.0E-10 | 0.001 | 5.899 | 2.5E-10 | 0.003 | 94.907 | 4.1E-09 | 0.002 | 0.677 | 2.9E-08 | 0.010 | 11.332 | 4.9E-07 | 0.024 |
| 32 | 89.477 | 3.8E-09 | 0.013 | 18.003 | 7.7E-10 | 0.001 | 21.229 | 9.1E-10 | 0.009 | 143.122 | 6.1E-09 | 0.003 | 1.041 | 4.5E-08 | 0.015 | 22.482 | 9.6E-07 | 0.048 |
| 33 | 77.143 | 3.3E-09 | 0.011 | 17.343 | 7.4E-10 | 0.001 | 14.907 | 6.4E-10 | 0.006 | 211.894 | 9.1E-09 | 0.005 | 1.025 | 4.4E-08 | 0.015 | 6.356 | 2.7E-07 | 0.014 |
| 34 | 63.041 | 2.7E-09 | 0.009 | 7.482 | 3.2E-10 | 0.000 | 10.411 | 4.5E-10 | 0.004 | 112.510 | 4.8E-09 | 0.002 | 0.337 | 1.4E-08 | 0.005 | 3.358 | 1.4E-07 | 0.007 |
| 35 | 58.678 | 2.5E-09 | 0.008 | 12.108 | 5.2E-10 | 0.001 | 12.528 | 5.4E-10 | 0.005 | 235.252 | 1.0E-08 | 0.005 | 0.874 | 3.7E-08 | 0.012 | 2.59 | 1.1E-07 | 0.006 |
| 36 | 40.245 | 1.7E-09 | 0.006 | 21.592 | 9.3E-10 | 0.001 | 7.631 | 3.3E-10 | 0.003 | 183.142 | 7.8E-09 | 0.004 | 0.542 | 2.3E-08 | 0.008 | 3.651 | 1.6E-07 | 0.008 |
| 37 | 242.005 | 1.0E-08 | 0.035 | 129.445 | 5.5E-09 | 0.006 | 20.394 | 8.7E-10 | 0.009 | 286.934 | 1.2E-08 | 0.006 | 0.731 | 3.1E-08 | 0.010 | 20.644 | 8.8E-07 | 0.044 |
| 38 | 67.446 | 2.9E-09 | 0.010 | 15.139 | 6.5E-10 | 0.001 | 8.744 | 3.7E-10 | 0.004 | 231.022 | 9.9E-09 | 0.005 | 0.802 | 3.4E-08 | 0.011 | 2.302 | 9.9E-08 | 0.005 |
| 39 | 56.974 | 2.4E-09 | 0.008 | 14.119 | 6.1E-10 | 0.001 | 10.808 | 4.6E-10 | 0.005 | 163.799 | 7.0E-09 | 0.004 | 1.126 | 4.8E-08 | 0.016 | 9.066 | 3.9E-07 | 0.019 |
| 40 | 47.289 | 2.0E-09 | 0.007 | 25.500 | 1.1E-09 | 0.001 | 21.348 | 9.1E-10 | 0.009 | 550.034 | 2.4E-08 | 0.012 | 0.578 | 2.5E-08 | 0.008 | 4.415 | 1.9E-07 | 0.009 |
| 41 | 75.392 | 3.2E-09 | 0.011 | 19.485 | 8.4E-10 | 0.001 | 12.864 | 5.5E-10 | 0.006 | 206.898 | 8.9E-09 | 0.004 | 0.572 | 2.5E-08 | 0.008 | 3.571 | 1.5E-07 | 0.008 |
| 42 | 77.002 | 3.3E-09 | 0.011 | 20.955 | 9.0E-10 | 0.001 | 10.615 | 4.5E-10 | 0.005 | 244.065 | 1.0E-08 | 0.005 | 1.026 | 4.4E-08 | 0.015 | 2.538 | 1.1E-07 | 0.005 |
| 43 | 61.295 | 2.6E-09 | 0.009 | 12.010 | 5.1E-10 | 0.001 | 27.480 | 1.2E-09 | 0.012 | 293.447 | 1.3E-08 | 0.006 | 1.482 | 6.4E-08 | 0.021 | 17.015 | 7.3E-07 | 0.036 |
| 44 | 69.966 | 3.0E-09 | 0.010 | 21.504 | 9.2E-10 | 0.001 | 10.497 | 4.5E-10 | 0.004 | 329.942 | 1.4E-08 | 0.007 | 1.312 | 5.6E-08 | 0.019 | 15.845 | 6.8E-07 | 0.034 |
| 45 | 116.633 | 5.0E-09 | 0.017 | 14.387 | 6.2E-10 | 0.001 | 15.698 | 6.7E-10 | 0.007 | 345.756 | 1.5E-08 | 0.007 | 1.455 | 6.2E-08 | 0.021 | 2.936 | 1.3E-07 | 0.006 |
| 46 | 105.491 | 4.5E-09 | 0.015 | 20.910 | 9.0E-10 | 0.001 | 10.285 | 4.4E-10 | 0.004 | 293.081 | 1.3E-08 | 0.006 | 1.047 | 4.5E-08 | 0.015 | 5.37 | 2.3E-07 | 0.012 |
| 47 | 78.509 | 3.4E-09 | 0.011 | 19.891 | 8.5E-10 | 0.001 | 13.438 | 5.8E-10 | 0.006 | 285.277 | 1.2E-08 | 0.006 | 0.926 | 4.0E-08 | 0.013 | 4.879 | 2.1E-07 | 0.010 |
| 48 | 101.460 | 4.3E-09 | 0.014 | 19.342 | 8.3E-10 | 0.001 | 19.162 | 8.2E-10 | 0.008 | 392.111 | 1.7E-08 | 0.008 | 1.305 | 5.6E-08 | 0.019 | 6.165 | 2.6E-07 | 0.013 |
| 49 | 94.598 | 4.1E-09 | 0.014 | 16.700 | 7.2E-10 | 0.001 | 9.709 | 4.2E-10 | 0.004 | 271.274 | 1.2E-08 | 0.006 | 1.637 | 7.0E-08 | 0.023 | 4.034 | 1.7E-07 | 0.009 |
| 50 | 156.857 | 6.7E-09 | 0.022 | 18.482 | 7.9E-10 | 0.001 | 17.929 | 7.7E-10 | 0.008 | 258.651 | 1.1E-08 | 0.006 | 2.148 | 9.2E-08 | 0.031 | 3.552 | 1.5E-07 | 0.008 |
| 51 | 65.523 | 2.8E-09 | 0.009 | 18.987 | 8.1E-10 | 0.001 | 21.672 | 9.3E-10 | 0.009 | 142.806 | 6.1E-09 | 0.003 | 0.536 | 2.3E-08 | 0.008 | 2.942 | 1.3E-07 | 0.006 |
| 52 | 101.466 | 4.3E-09 | 0.014 | 21.218 | 9.1E-10 | 0.001 | 19.687 | 8.4E-10 | 0.008 | 442.216 | 1.9E-08 | 0.009 | 2.489 | 1.1E-07 | 0.036 | 9.703 | 4.2E-07 | 0.021 |
| 53 | 96.473 | 4.1E-09 | 0.014 | 26.385 | 1.1E-09 | 0.001 | 15.530 | 6.7E-10 | 0.007 | 390.520 | 1.7E-08 | 0.008 | 1.929 | 8.3E-08 | 0.028 | 15.516 | 6.6E-07 | 0.033 |
| 54 | 554.075 | 2.4E-08 | 0.079 | 31.430 | 1.3E-09 | 0.001 | 87.859 | 3.8E-09 | 0.038 | 358.598 | 1.5E-08 | 0.008 | 0.901 | 3.9E-08 | 0.013 | 4.564 | 2.0E-07 | 0.010 |
| 55 | 66.971 | 2.9E-09 | 0.010 | 25.486 | 1.1E-09 | 0.001 | 12.974 | 5.6E-10 | 0.006 | 232.986 | 1.0E-08 | 0.005 | 1.320 | 5.7E-08 | 0.019 | 11.156 | 4.8E-07 | 0.024 |
| 56 | 97.709 | 4.2E-09 | 0.014 | 28.784 | 1.2E-09 | 0.001 | 13.476 | 5.8E-10 | 0.006 | 405.014 | 1.7E-08 | 0.009 | 1.133 | 4.9E-08 | 0.016 | 4.884 | 2.1E-07 | 0.010 |
| 57 | 70.315 | 3.0E-09 | 0.010 | 12.290 | 5.3E-10 | 0.001 | 10.834 | 4.6E-10 | 0.005 | 144.702 | 6.2E-09 | 0.003 | 0.494 | 2.1E-08 | 0.007 | 6.098 | 2.6E-07 | 0.013 |
| 58 | 90.523 | 3.9E-09 | 0.013 | 56.826 | 2.4E-09 | 0.002 | 12.274 | 5.3E-10 | 0.005 | 219.241 | 9.4E-09 | 0.005 | 0.806 | 3.5E-08 | 0.012 | 14.288 | 6.1E-07 | 0.031 |
| 59 | 207.811 | 8.9E-09 | 0.030 | 43.398 | 1.9E-09 | 0.002 | 22.938 | 9.8E-10 | 0.010 | 355.902 | 1.5E-08 | 0.008 | 2.463 | 1.1E-07 | 0.035 | 4.526 | 1.9E-07 | 0.010 |
| 60 | 105.713 | 4.5E-09 | 0.015 | 28.497 | 1.2E-09 | 0.001 | 16.744 | 7.2E-10 | 0.007 | 521.961 | 2.2E-08 | 0.011 | 2.132 | 9.1E-08 | 0.030 | 5.26 | 2.3E-07 | 0.011 |
| 61 | 285.429 | 1.2E-08 | 0.041 | 77.319 | 3.3E-09 | 0.003 | 15.539 | 6.7E-10 | 0.007 | 1142.697 | 4.9E-08 | 0.024 | 1.732 | 7.4E-08 | 0.025 | 4.054 | 1.7E-07 | 0.009 |
| 62 | 187.064 | 8.0E-09 | 0.027 | 21.455 | 9.2E-10 | 0.001 | 36.262 | 1.6E-09 | 0.016 | 409.073 | 1.8E-08 | 0.009 | 3.281 | 1.4E-07 | 0.047 | 11.131 | 4.8E-07 | 0.024 |
| 63 | 166.621 | 7.1E-09 | 0.024 | 11.468 | 4.9E-10 | 0.000 | 17.280 | 7.4E-10 | 0.007 | 332.490 | 1.4E-08 | 0.007 | 2.725 | 1.2E-07 | 0.039 | 8.524 | 3.7E-07 | 0.018 |
| 64 | 192.430 | 8.2E-09 | 0.027 | 30.640 | 1.3E-09 | 0.001 | 17.997 | 7.7E-10 | 0.008 | 542.873 | 2.3E-08 | 0.012 | 2.608 | 1.1E-07 | 0.037 | 5.425 | 2.3E-07 | 0.012 |
| 65 | 125.394 | 5.4E-09 | 0.018 | 14.910 | 6.4E-10 | 0.001 | 9.391 | 4.0E-10 | 0.004 | 370.330 | 1.6E-08 | 0.008 | 1.274 | 5.5E-08 | 0.018 | 4.213 | 1.8E-07 | 0.009 |
| 66 | 92.102 | 3.9E-09 | 0.013 | 32.197 | 1.4E-09 | 0.001 | 7.750 | 3.3E-10 | 0.003 | 338.235 | 1.4E-08 | 0.007 | 1.043 | 4.5E-08 | 0.015 | 4.88 | 2.1E-07 | 0.010 |
| 67 | 108.159 | 4.6E-09 | 0.015 | 36.243 | 1.6E-09 | 0.002 | 12.823 | 5.5E-10 | 0.005 | 386.999 | 1.7E-08 | 0.008 | 1.509 | 6.5E-08 | 0.022 | 24.397 | 1.0E-06 | 0.052 |
| 68 | 126.290 | 5.4E-09 | 0.018 | 25.063 | 1.1E-09 | 0.001 | 15.105 | 6.5E-10 | 0.006 | 387.237 | 1.7E-08 | 0.008 | 1.860 | 8.0E-08 | 0.027 | 9.711 | 4.2E-07 | 0.021 |
| 69 | 97.325 | 4.2E-09 | 0.014 | 58.107 | 2.5E-09 | 0.002 | 11.395 | 4.9E-10 | 0.005 | 355.352 | 1.5E-08 | 0.008 | 1.576 | 6.8E-08 | 0.023 | 11.36 | 4.9E-07 | 0.024 |
| 70 | 187.000 | 8.0E-09 | 0.027 | 24.691 | 1.1E-09 | 0.001 | 13.619 | 5.8E-10 | 0.006 | 666.260 | 2.9E-08 | 0.014 | 5.050 | 2.2E-07 | 0.072 | 31.071 | 1.3E-06 | 0.067 |
| 71 | 258.088 | 1.1E-08 | 0.037 | 29.860 | 1.3E-09 | 0.001 | 16.180 | 6.9E-10 | 0.007 | 1067.189 | 4.6E-08 | 0.023 | 7.886 | 3.4E-07 | 0.113 | 48.958 | 2.1E-06 | 0.105 |
| 72 | 104.125 | 4.5E-09 | 0.015 | 19.984 | 8.6E-10 | 0.001 | 17.372 | 7.4E-10 | 0.007 | 1195.097 | 5.1E-08 | 0.026 | 1.780 | 7.6E-08 | 0.025 | 12.59 | 5.4E-07 | 0.027 |
| 73 | 171.936 | 7.4E-09 | 0.025 | 68.084 | 2.9E-09 | 0.003 | 15.164 | 6.5E-10 | 0.006 | 430.307 | 1.8E-08 | 0.009 | 2.563 | 1.1E-07 | 0.037 | 8.321 | 3.6E-07 | 0.018 |
| 74 | 146.864 | 6.3E-09 | 0.021 | 18.805 | 8.1E-10 | 0.001 | 13.364 | 5.7E-10 | 0.006 | 619.811 | 2.7E-08 | 0.013 | 3.009 | 1.3E-07 | 0.043 | 7.726 | 3.3E-07 | 0.017 |
| 75 | 139.949 | 6.0E-09 | 0.020 | 39.018 | 1.7E-09 | 0.002 | 9.018 | 3.9E-10 | 0.004 | 470.206 | 2.0E-08 | 0.010 | 0.861 | 3.7E-08 | 0.012 | 4.104 | 1.8E-07 | 0.009 |
| 76 | 149.200 | 6.4E-09 | 0.021 | 46.084 | 2.0E-09 | 0.002 | 10.312 | 4.4E-10 | 0.004 | 425.019 | 1.8E-08 | 0.009 | 0.943 | 4.0E-08 | 0.013 | 11.192 | 4.8E-07 | 0.024 |
| 77 | 327.081 | 1.4E-08 | 0.047 | 430.671 | 1.8E-08 | 0.018 | 23.893 | 1.0E-09 | 0.010 | 824.806 | 3.5E-08 | 0.018 | 2.858 | 1.2E-07 | 0.041 | 8.109 | 3.5E-07 | 0.017 |
| 78 | 262.030 | 1.1E-08 | 0.037 | 125.520 | 5.4E-09 | 0.005 | 22.105 | 9.5E-10 | 0.009 | 478.932 | 2.1E-08 | 0.010 | 1.281 | 5.5E-08 | 0.018 | 7.473 | 3.2E-07 | 0.016 |
| 79 | 270.635 | 1.2E-08 | 0.039 | 137.483 | 5.9E-09 | 0.006 | 17.455 | 7.5E-10 | 0.007 | 514.788 | 2.2E-08 | 0.011 | 1.696 | 7.3E-08 | 0.024 | 6.073 | 2.6E-07 | 0.013 |
| 80 | 268.334 | 1.2E-08 | 0.038 | 243.082 | 1.0E-08 | 0.010 | 18.816 | 8.1E-10 | 0.008 | 378.179 | 1.6E-08 | 0.008 | 1.349 | 5.8E-08 | 0.019 | 7.254 | 3.1E-07 | 0.016 |
| 81 | 96.940 | 4.2E-09 | 0.014 | 168.091 | 7.2E-09 | 0.007 | 106.574 | 4.6E-09 | 0.046 | 330.812 | 1.4E-08 | 0.007 | 1.247 | 5.3E-08 | 0.018 | 3.557 | 1.5E-07 | 0.008 |
| 82 | 171.607 | 7.4E-09 | 0.025 | 30.273 | 1.3E-09 | 0.001 | 13.041 | 5.6E-10 | 0.006 | 693.646 | 3.0E-08 | 0.015 | 2.141 | 9.2E-08 | 0.031 | 7.454 | 3.2E-07 | 0.016 |
| 83 | 182.051 | 7.8E-09 | 0.026 | 184.830 | 7.9E-09 | 0.008 | 17.430 | 7.5E-10 | 0.007 | 429.277 | 1.8E-08 | 0.009 | 1.916 | 8.2E-08 | 0.027 | 9.508 | 4.1E-07 | 0.020 |
| 84 | 173.153 | 7.4E-09 | 0.025 | 59.312 | 2.5E-09 | 0.003 | 12.490 | 5.4E-10 | 0.005 | 398.987 | 1.7E-08 | 0.009 | 1.266 | 5.4E-08 | 0.018 | 1.759 | 7.5E-08 | 0.004 |
| 85 | 93.155 | 4.0E-09 | 0.013 | 23.023 | 9.9E-10 | 0.001 | 11.963 | 5.1E-10 | 0.005 | 389.970 | 1.7E-08 | 0.008 | 1.296 | 5.6E-08 | 0.019 | 4.072 | 1.7E-07 | 0.009 |
| 86 | 80.898 | 3.5E-09 | 0.012 | 45.263 | 1.9E-09 | 0.002 | 20.292 | 8.7E-10 | 0.009 | 165.286 | 7.1E-09 | 0.004 | 0.364 | 1.6E-08 | 0.005 | 2.32 | 9.9E-08 | 0.005 |
| 87 | 339.889 | 1.5E-08 | 0.049 | 109.090 | 4.7E-09 | 0.005 | 35.134 | 1.5E-09 | 0.015 | 1751.811 | 7.5E-08 | 0.038 | 4.747 | 2.0E-07 | 0.068 | 6.216 | 2.7E-07 | 0.013 |
| 88 | 119.731 | 5.1E-09 | 0.017 | 179.369 | 7.7E-09 | 0.008 | 12.941 | 5.5E-10 | 0.006 | 424.656 | 1.8E-08 | 0.009 | 1.318 | 5.6E-08 | 0.019 | 3.897 | 1.7E-07 | 0.008 |
| 89 | 86.736 | 3.7E-09 | 0.012 | 41.308 | 1.8E-09 | 0.002 | 15.368 | 6.6E-10 | 0.007 | 345.597 | 1.5E-08 | 0.007 | 1.968 | 8.4E-08 | 0.028 | 5.313 | 2.3E-07 | 0.011 |
| 90 | 429.696 | 1.8E-08 | 0.061 | 32.159 | 1.4E-09 | 0.001 | 12.115 | 5.2E-10 | 0.005 | 410.593 | 1.8E-08 | 0.009 | 2.080 | 8.9E-08 | 0.030 | 5.421 | 2.3E-07 | 0.012 |

**Table S6:** Calculated EDI and CR for the toxic elements.

|  | **As** |  | **Pb** |  | **Cr** |  | **Ni** |  |
| --- | --- | --- | --- | --- | --- | --- | --- | --- |
| **No of samples** | **EDI** | **CR** | **EDI** | **CR** | **EDI** | **CR** | **EDI** | **CR** |
| 1 | 2.9E-09 | 4.3E-09 | 6.6E-09 | 5.6E-11 | 3.8E-08 | 1.9E-08 | 2.2E-07 | 2.0E-07 |
| 2 | 1.1E-08 | 1.7E-08 | 2.1E-08 | 1.8E-10 | 2.5E-08 | 1.2E-08 | 4.0E-08 | 3.7E-08 |
| 3 | 4.1E-09 | 6.2E-09 | 1.1E-08 | 9.2E-11 | 7.1E-08 | 3.5E-08 | 2.0E-07 | 1.9E-07 |
| 4 | 2.7E-09 | 4.0E-09 | 7.2E-09 | 6.1E-11 | 1.2E-08 | 6.0E-09 | 1.2E-07 | 1.1E-07 |
| 5 | 3.8E-09 | 5.7E-09 | 9.9E-09 | 8.4E-11 | 6.8E-08 | 3.4E-08 | 1.6E-07 | 1.5E-07 |
| 6 | 3.2E-09 | 4.8E-09 | 1.0E-08 | 8.8E-11 | 1.7E-08 | 8.7E-09 | 6.0E-08 | 5.4E-08 |
| 7 | 1.7E-09 | 2.6E-09 | 3.1E-09 | 2.7E-11 | 1.2E-08 | 5.9E-09 | 7.1E-08 | 6.4E-08 |
| 8 | 2.2E-09 | 3.3E-09 | 5.0E-09 | 4.3E-11 | 1.4E-08 | 7.2E-09 | 9.6E-08 | 8.7E-08 |
| 9 | 4.2E-09 | 6.2E-09 | 1.1E-08 | 9.3E-11 | 1.5E-08 | 7.5E-09 | 2.8E-07 | 2.6E-07 |
| 10 | 2.2E-09 | 3.3E-09 | 7.5E-09 | 6.4E-11 | 4.3E-08 | 2.1E-08 | 2.2E-07 | 2.0E-07 |
| 11 | 1.9E-09 | 2.9E-09 | 5.4E-09 | 4.6E-11 | 3.3E-08 | 1.7E-08 | 7.7E-08 | 7.0E-08 |
| 12 | 2.1E-09 | 3.1E-09 | 5.2E-09 | 4.4E-11 | 1.6E-08 | 8.1E-09 | 8.7E-08 | 7.9E-08 |
| 13 | 2.2E-09 | 3.3E-09 | 2.5E-09 | 2.2E-11 | 1.4E-08 | 6.8E-09 | 6.8E-08 | 6.2E-08 |
| 14 | 2.4E-09 | 3.6E-09 | 6.6E-09 | 5.6E-11 | 2.1E-08 | 1.1E-08 | 1.9E-07 | 1.7E-07 |
| 15 | 4.8E-09 | 7.2E-09 | 6.4E-09 | 5.4E-11 | 1.0E-08 | 5.0E-09 | 1.2E-07 | 1.1E-07 |
| 16 | 1.4E-09 | 2.1E-09 | 4.0E-09 | 3.4E-11 | 9.1E-09 | 4.5E-09 | 3.7E-08 | 3.4E-08 |
| 17 | 1.7E-09 | 2.6E-09 | 5.3E-09 | 4.5E-11 | 1.8E-08 | 9.2E-09 | 1.1E-07 | 1.0E-07 |
| 18 | 1.5E-09 | 2.2E-09 | 4.0E-09 | 3.4E-11 | 1.3E-08 | 6.3E-09 | 8.0E-08 | 7.3E-08 |
| 19 | 1.2E-09 | 1.8E-09 | 1.2E-08 | 1.0E-10 | 2.3E-08 | 1.2E-08 | 1.8E-07 | 1.6E-07 |
| 20 | 8.6E-10 | 1.3E-09 | 4.0E-09 | 3.4E-11 | 1.4E-08 | 7.2E-09 | 2.1E-07 | 1.9E-07 |
| 21 | 7.3E-09 | 1.1E-08 | 1.1E-08 | 8.9E-11 | 7.0E-08 | 3.5E-08 | 2.0E-07 | 1.8E-07 |
| 22 | 6.3E-09 | 9.4E-09 | 1.7E-08 | 1.4E-10 | 8.4E-08 | 4.2E-08 | 1.8E-07 | 1.6E-07 |
| 23 | 2.1E-09 | 3.1E-09 | 3.9E-09 | 3.3E-11 | 3.3E-08 | 1.6E-08 | 2.0E-07 | 1.8E-07 |
| 24 | 2.0E-09 | 3.0E-09 | 7.0E-09 | 6.0E-11 | 2.3E-08 | 1.2E-08 | 1.1E-07 | 9.9E-08 |
| 25 | 1.1E-08 | 1.7E-08 | 1.2E-08 | 1.1E-10 | 6.1E-08 | 3.0E-08 | 1.7E-07 | 1.6E-07 |
| 26 | 9.7E-10 | 1.5E-09 | 4.0E-09 | 3.4E-11 | 1.5E-08 | 7.4E-09 | 1.8E-07 | 1.6E-07 |
| 27 | 1.9E-09 | 2.8E-09 | 9.4E-09 | 8.0E-11 | 3.5E-08 | 1.8E-08 | 2.8E-07 | 2.6E-07 |
| 28 | 1.8E-09 | 2.8E-09 | 1.1E-08 | 9.3E-11 | 2.4E-08 | 1.2E-08 | 7.8E-08 | 7.1E-08 |
| 29 | 5.3E-10 | 8.0E-10 | 4.3E-09 | 3.7E-11 | 1.3E-08 | 6.3E-09 | 3.6E-07 | 3.3E-07 |
| 30 | 7.9E-10 | 1.2E-09 | 2.2E-09 | 1.9E-11 | 1.0E-08 | 5.2E-09 | 2.4E-07 | 2.2E-07 |
| 31 | 5.1E-10 | 7.7E-10 | 1.6E-09 | 1.3E-11 | 1.1E-08 | 5.6E-09 | 1.9E-07 | 1.7E-07 |
| 32 | 1.5E-09 | 2.2E-09 | 2.4E-09 | 2.0E-11 | 1.7E-08 | 8.6E-09 | 3.7E-07 | 3.4E-07 |
| 33 | 1.3E-09 | 1.9E-09 | 3.5E-09 | 3.0E-11 | 1.7E-08 | 8.5E-09 | 1.1E-07 | 9.6E-08 |
| 34 | 1.0E-09 | 1.6E-09 | 1.9E-09 | 1.6E-11 | 5.6E-09 | 2.8E-09 | 5.6E-08 | 5.1E-08 |
| 35 | 9.7E-10 | 1.5E-09 | 3.9E-09 | 3.3E-11 | 1.4E-08 | 7.2E-09 | 4.3E-08 | 3.9E-08 |
| 36 | 6.7E-10 | 1.0E-09 | 3.0E-09 | 2.6E-11 | 9.0E-09 | 4.5E-09 | 6.0E-08 | 5.5E-08 |
| 37 | 4.0E-09 | 6.0E-09 | 4.7E-09 | 4.0E-11 | 1.2E-08 | 6.0E-09 | 3.4E-07 | 3.1E-07 |
| 38 | 1.1E-09 | 1.7E-09 | 3.8E-09 | 3.2E-11 | 1.3E-08 | 6.6E-09 | 3.8E-08 | 3.5E-08 |
| 39 | 9.4E-10 | 1.4E-09 | 2.7E-09 | 2.3E-11 | 1.9E-08 | 9.3E-09 | 1.5E-07 | 1.4E-07 |
| 40 | 7.8E-10 | 1.2E-09 | 9.1E-09 | 7.7E-11 | 9.6E-09 | 4.8E-09 | 7.3E-08 | 6.6E-08 |
| 41 | 1.2E-09 | 1.9E-09 | 3.4E-09 | 2.9E-11 | 9.5E-09 | 4.7E-09 | 5.9E-08 | 5.4E-08 |
| 42 | 1.3E-09 | 1.9E-09 | 4.0E-09 | 3.4E-11 | 1.7E-08 | 8.5E-09 | 4.2E-08 | 3.8E-08 |
| 43 | 1.0E-09 | 1.5E-09 | 4.9E-09 | 4.1E-11 | 2.5E-08 | 1.2E-08 | 2.8E-07 | 2.6E-07 |
| 44 | 1.2E-09 | 1.7E-09 | 5.5E-09 | 4.6E-11 | 2.2E-08 | 1.1E-08 | 2.6E-07 | 2.4E-07 |
| 45 | 1.9E-09 | 2.9E-09 | 5.7E-09 | 4.9E-11 | 2.4E-08 | 1.2E-08 | 4.9E-08 | 4.4E-08 |
| 46 | 1.7E-09 | 2.6E-09 | 4.8E-09 | 4.1E-11 | 1.7E-08 | 8.7E-09 | 8.9E-08 | 8.1E-08 |
| 47 | 1.3E-09 | 1.9E-09 | 4.7E-09 | 4.0E-11 | 1.5E-08 | 7.7E-09 | 8.1E-08 | 7.3E-08 |
| 48 | 1.7E-09 | 2.5E-09 | 6.5E-09 | 5.5E-11 | 2.2E-08 | 1.1E-08 | 1.0E-07 | 9.3E-08 |
| 49 | 1.6E-09 | 2.3E-09 | 4.5E-09 | 3.8E-11 | 2.7E-08 | 1.4E-08 | 6.7E-08 | 6.1E-08 |
| 50 | 2.6E-09 | 3.9E-09 | 4.3E-09 | 3.6E-11 | 3.6E-08 | 1.8E-08 | 5.9E-08 | 5.3E-08 |
| 51 | 1.1E-09 | 1.6E-09 | 2.4E-09 | 2.0E-11 | 8.9E-09 | 4.4E-09 | 4.9E-08 | 4.4E-08 |
| 52 | 1.7E-09 | 2.5E-09 | 7.3E-09 | 6.2E-11 | 4.1E-08 | 2.1E-08 | 1.6E-07 | 1.5E-07 |
| 53 | 1.6E-09 | 2.4E-09 | 6.5E-09 | 5.5E-11 | 3.2E-08 | 1.6E-08 | 2.6E-07 | 2.3E-07 |
| 54 | 9.2E-09 | 1.4E-08 | 5.9E-09 | 5.0E-11 | 1.5E-08 | 7.4E-09 | 7.5E-08 | 6.9E-08 |
| 55 | 1.1E-09 | 1.7E-09 | 3.9E-09 | 3.3E-11 | 2.2E-08 | 1.1E-08 | 1.8E-07 | 1.7E-07 |
| 56 | 1.6E-09 | 2.4E-09 | 6.7E-09 | 5.7E-11 | 1.9E-08 | 9.4E-09 | 8.1E-08 | 7.3E-08 |
| 57 | 1.2E-09 | 1.7E-09 | 2.4E-09 | 2.0E-11 | 8.2E-09 | 4.1E-09 | 1.0E-07 | 9.2E-08 |
| 58 | 1.5E-09 | 2.2E-09 | 3.6E-09 | 3.1E-11 | 1.3E-08 | 6.7E-09 | 2.4E-07 | 2.1E-07 |
| 59 | 3.4E-09 | 5.2E-09 | 5.9E-09 | 5.0E-11 | 4.1E-08 | 2.0E-08 | 7.5E-08 | 6.8E-08 |
| 60 | 1.7E-09 | 2.6E-09 | 8.6E-09 | 7.3E-11 | 3.5E-08 | 1.8E-08 | 8.7E-08 | 7.9E-08 |
| 61 | 4.7E-09 | 7.1E-09 | 1.9E-08 | 1.6E-10 | 2.9E-08 | 1.4E-08 | 6.7E-08 | 6.1E-08 |
| 62 | 3.1E-09 | 4.6E-09 | 6.8E-09 | 5.7E-11 | 5.4E-08 | 2.7E-08 | 1.8E-07 | 1.7E-07 |
| 63 | 2.8E-09 | 4.1E-09 | 5.5E-09 | 4.7E-11 | 4.5E-08 | 2.3E-08 | 1.4E-07 | 1.3E-07 |
| 64 | 3.2E-09 | 4.8E-09 | 9.0E-09 | 7.6E-11 | 4.3E-08 | 2.2E-08 | 9.0E-08 | 8.2E-08 |
| 65 | 2.1E-09 | 3.1E-09 | 6.1E-09 | 5.2E-11 | 2.1E-08 | 1.1E-08 | 7.0E-08 | 6.3E-08 |
| 66 | 1.5E-09 | 2.3E-09 | 5.6E-09 | 4.8E-11 | 1.7E-08 | 8.6E-09 | 8.1E-08 | 7.3E-08 |
| 67 | 1.8E-09 | 2.7E-09 | 6.4E-09 | 5.4E-11 | 2.5E-08 | 1.2E-08 | 4.0E-07 | 3.7E-07 |
| 68 | 2.1E-09 | 3.1E-09 | 6.4E-09 | 5.4E-11 | 3.1E-08 | 1.5E-08 | 1.6E-07 | 1.5E-07 |
| 69 | 1.6E-09 | 2.4E-09 | 5.9E-09 | 5.0E-11 | 2.6E-08 | 1.3E-08 | 1.9E-07 | 1.7E-07 |
| 70 | 3.1E-09 | 4.6E-09 | 1.1E-08 | 9.4E-11 | 8.3E-08 | 4.2E-08 | 5.1E-07 | 4.7E-07 |
| 71 | 4.3E-09 | 6.4E-09 | 1.8E-08 | 1.5E-10 | 1.3E-07 | 6.5E-08 | 8.1E-07 | 7.4E-07 |
| 72 | 1.7E-09 | 2.6E-09 | 2.0E-08 | 1.7E-10 | 2.9E-08 | 1.5E-08 | 2.1E-07 | 1.9E-07 |
| 73 | 2.8E-09 | 4.3E-09 | 7.1E-09 | 6.0E-11 | 4.2E-08 | 2.1E-08 | 1.4E-07 | 1.3E-07 |
| 74 | 2.4E-09 | 3.6E-09 | 1.0E-08 | 8.7E-11 | 5.0E-08 | 2.5E-08 | 1.3E-07 | 1.2E-07 |
| 75 | 2.3E-09 | 3.5E-09 | 7.8E-09 | 6.6E-11 | 1.4E-08 | 7.1E-09 | 6.8E-08 | 6.2E-08 |
| 76 | 2.5E-09 | 3.7E-09 | 7.0E-09 | 6.0E-11 | 1.6E-08 | 7.8E-09 | 1.9E-07 | 1.7E-07 |
| 77 | 5.4E-09 | 8.1E-09 | 1.4E-08 | 1.2E-10 | 4.7E-08 | 2.4E-08 | 1.3E-07 | 1.2E-07 |
| 78 | 4.3E-09 | 6.5E-09 | 7.9E-09 | 6.7E-11 | 2.1E-08 | 1.1E-08 | 1.2E-07 | 1.1E-07 |
| 79 | 4.5E-09 | 6.7E-09 | 8.5E-09 | 7.2E-11 | 2.8E-08 | 1.4E-08 | 1.0E-07 | 9.1E-08 |
| 80 | 4.4E-09 | 6.7E-09 | 6.3E-09 | 5.3E-11 | 2.2E-08 | 1.1E-08 | 1.2E-07 | 1.1E-07 |
| 81 | 1.6E-09 | 2.4E-09 | 5.5E-09 | 4.6E-11 | 2.1E-08 | 1.0E-08 | 5.9E-08 | 5.4E-08 |
| 82 | 2.8E-09 | 4.3E-09 | 1.1E-08 | 9.7E-11 | 3.5E-08 | 1.8E-08 | 1.2E-07 | 1.1E-07 |
| 83 | 3.0E-09 | 4.5E-09 | 7.1E-09 | 6.0E-11 | 3.2E-08 | 1.6E-08 | 1.6E-07 | 1.4E-07 |
| 84 | 2.9E-09 | 4.3E-09 | 6.6E-09 | 5.6E-11 | 2.1E-08 | 1.0E-08 | 2.9E-08 | 2.6E-08 |
| 85 | 1.5E-09 | 2.3E-09 | 6.4E-09 | 5.5E-11 | 2.1E-08 | 1.1E-08 | 6.7E-08 | 6.1E-08 |
| 86 | 1.3E-09 | 2.0E-09 | 2.7E-09 | 2.3E-11 | 6.0E-09 | 3.0E-09 | 3.8E-08 | 3.5E-08 |
| 87 | 5.6E-09 | 8.4E-09 | 2.9E-08 | 2.5E-10 | 7.8E-08 | 3.9E-08 | 1.0E-07 | 9.4E-08 |
| 88 | 2.0E-09 | 3.0E-09 | 7.0E-09 | 6.0E-11 | 2.2E-08 | 1.1E-08 | 6.4E-08 | 5.9E-08 |
| 89 | 1.4E-09 | 2.2E-09 | 5.7E-09 | 4.9E-11 | 3.3E-08 | 1.6E-08 | 8.8E-08 | 8.0E-08 |
| 90 | 7.1E-09 | 1.1E-08 | 6.8E-09 | 5.8E-11 | 3.4E-08 | 1.7E-08 | 9.0E-08 | 8.2E-08 |

**Table S7.** Oral Cancer Potency Factors (CPFo)

| **Heavy metal** | **CPFo (mg/kg per day)** | **Reference** |
| --- | --- | --- |
| Pb | 8.5 x 10^-3^ | https://oehha.ca.gov/chemicals/lead-and-lead-compounds |
| As | 1.5 | https://iris.epa.gov/ChemicalLanding/&substance_nmbr=278 |
| Cr | 0.5 | Doabi et al., 2018 and references therein |
| Ni | 0.91 | Doabi et al., 2018 and references therein |
